# Supplementary material for: COVID-NURSE: evaluation of a fundamental nursing care protocol compared with care as usual on experience of care for noninvasively ventilated patients in hospital with the SARS-CoV-2 virus—protocol for a cluster randomised controlled trial
Source: BMJ Open. 2021 May 26;11(5):e046436. doi: 10.1136/bmjopen-2020-046436 (PMC8159671; doi:10.1136/bmjopen-2020-046436)
Supplement: Supplementary data [file bmjopen-2020-046436supp001.pdf]

UNIVERSITY OF EXETER

COLLEGE OF MEDICINE AND HEALTH

Research title: COVID-NURSE: evaluation of the effects of a COVID-specific fundamental nursing care protocol compared to care as usual on experience of care for non-invasively ventilated patients in hospital with the SARS-CoV-2 virus: a randomised controlled trial.

CONSENT FORM
FOR PATIENT PARTICIPANTS

Participant Identification Number: ☐☐☐☐☐☐☐☐☐

Name of Researcher: ______________________________

Please initial box

1. I confirm that I have read the information sheet dated [2021-01-25] version no [5] for the above project. I have had the opportunity to consider the information, ask questions and have had these answered satisfactorily. ☐

2. I understand that my participation is voluntary and that I am free to withdraw at any time without giving any reason and without my legal rights being affected. ☐

3. I understand that relevant sections of my medical record and the data collected during the study may be looked at by members of the research team, individuals from the University of Exeter, Exeter Clinical Trials Unit or the Health Research Authority, where it is relevant to my taking part in this research.
I give permission for these individuals to have access to my records. ☐

4. I understand that data I provide for the study will be stored on servers all of which are hosted within the Europe Economic Area ☐

5. I understand that taking part involves questionnaire responses to be used for the purposes of research ☐

6. I understand that taking part involves inclusion in an archive for a period of up to 10 years ☐

7. I understand that taking part involves anonymized data being shared with other researchers for use in future research projects; ☐

8. I understand that taking part involves anonymized data being used in reports published in academic publication, project website and media publication; ☐

9. I understand that taking part involves anonymized data being used in public engagement activities ☐

Please initial box

10. I understand that ward level data will be collected for the trial and may be looked at by individuals from the University of Exeter, the NHS Trust and from regulatory authorities where it is relevant to my taking part in the research. I give permission for these individuals to have access to my records. ☐

11. I understand the research team would like my contact details do they can Let me know the results of the study **[optional item]** ☐

12. I understand that there may be circumstances in which confidential information may need to be disclosed if information shared suggests a risk to myself or others. ☐

13. I agree to take part in the above project. ☐

.... (Printed name of participant)	……………………….. (Signature of participant)	.... (Date)
.... (Printed name of researcher taking consent)	……………………….. (Signature of researcher)	.... (Date)

When completed: 1 copy for participant; 1 copy for researcher/project file
