# Supplementary material for: COVID-NURSE: evaluation of a fundamental nursing care protocol compared with care as usual on experience of care for noninvasively ventilated patients in hospital with the SARS-CoV-2 virus—protocol for a cluster randomised controlled trial
Source: BMJ Open. 2021 May 26;11(5):e046436. doi: 10.1136/bmjopen-2020-046436 (PMC8159671; doi:10.1136/bmjopen-2020-046436)
Supplement: Supplementary data [file bmjopen-2020-046436supp002.pdf]

UNIVERSITY OF EXETER

COLLEGE OF MEDICINE AND HEALTH

Research title: COVID-NURSE: evaluation of the effects of a COVID-specific fundamental nursing care protocol compared to care as usual on experience of care for non-invasively ventilated patients in hospital with the SARS-CoV-2 virus: a randomised controlled trial.

CONSENT FORM
FOR NURSE PARTICIPANTS

Participant Identification Number: ☐☐☐☐☐☐☐☐☐

Name of Researcher: ____________________

Please initial box

1. I confirm that I have read the information sheet dated [2021- 01-13] version no [4] for the above project. I have had the opportunity to consider the information, ask questions and have had these answered satisfactorily. ☐

2. understand that my participation in providing my data is voluntary and that I am free to withdraw at any time without giving any reason and without my legal rights being affected. ☐

3. I understand that I may also be asked to take part in a short online interview with follow up questions, which will be recorded for later transcription, at the end of the 6 week trial period by a researcher. ☐

4. If interviewed, the transcription will be anonymized to protect participant confidentiality. ☐

5. I understand that relevant sections of the data collected during the study may be looked at by members of the research team, individuals from the University of Exeter, Exeter Clinical Trials Unit or regulatory authorities, where it is relevant to my taking part in this research.
I give permission for these individuals to have access to my data. ☐

6. I understand that data I provide for the study will be stored on servers all of which are hosted within the Europe Economic Area ☐

7. I understand that taking part involves questionnaire responses to be used for the purposes of research ☐

8. I understand that taking part involves inclusion in an archive for a period of up to 10 years. Data will be stored securely at the University of Exeter. ☐

9. I understand that taking part involves anonymized data being shared with other researchers for use in future research projects; ☐

10. I understand that if my Trust has been allocated to the experimental intervention condition I will be asked to undertake some online training on the FutureLearn platform. If I choose to undertake this training, I will consent to the General Data Protection Regulation privacy policies of FutureLearn, which I can read on the FutureLearn course website. I understand that a specific course 'Learning Manager' at the University of Exeter may support and interact with me about the course. I understand that the Learning Manager will remove my email, name and any other personally identifiable information from my course records, before collating and sending anonymised records of training to be analysed by the research team. I understand that no records of my training will be seen by a researcher at my hospital.

11. I understand the research team would like my contact details so they may contact me to participate in an interview and let me know the results of the study **[optional item]** ☐

12. I understand that taking part involves anonymized data being used in reports published in academic publication, project website and media publication; ☐

13. I understand that taking part involves anonymized data being used in public engagement activities. ☐

14. I understand that there may be circumstances in which confidential information may need to be disclosed if information shared suggests a risk to myself or others. ☐

15. I agree to take part in the above project. ☐

....	…………………………..	....
(Printed name of participant)	(Signature of participant)	(Date)
....	…………………………..	....
(Printed name of researcher taking consent)	(Signature of researcher)	(Date)

When completed: 1 copy for participant; 1 copy for researcher/project file
