# Supplementary material for: COVID-NURSE: evaluation of a fundamental nursing care protocol compared with care as usual on experience of care for noninvasively ventilated patients in hospital with the SARS-CoV-2 virus—protocol for a cluster randomised controlled trial
Source: BMJ Open. 2021 May 26;11(5):e046436. doi: 10.1136/bmjopen-2020-046436 (PMC8159671; doi:10.1136/bmjopen-2020-046436)
Supplement: Author's manuscript [file bmjopen-2020-046436.draft_revisions.pdf]

BMJ Open is committed to open peer review. As part of this commitment we make the peer review history of every article we publish publicly available.

When an article is published we post the peer reviewers' comments and the authors' responses online. We also post the versions of the paper that were used during peer review. These are the versions that the peer review comments apply to.

The versions of the paper that follow are the versions that were submitted during the peer review process. They are not the versions of record or the final published versions. They should not be cited or distributed as the published version of this manuscript.

BMJ Open is an open access journal and the full, final, typeset and author-corrected version of record of the manuscript is available on our site with no access controls, subscription charges or pay-per-view fees (http://bmjopen.bmj.com).

If you have any questions on BMJ Open's open peer review process please email
info.bmjopen@bmj.com

BMJ Open

COVID-NURSE: development and evaluation of the effects of a COVID-specific fundamental nursing care protocol compared to care as usual on experience of care for non-invasively ventilated patients in hospital with the SARS-CoV-2 virus: protocol for a cluster randomised controlled trial

Journal:	*BMJ Open*
Manuscript ID	bmjopen-2020-046436
Article Type:	Protocol
Date Submitted by the Author:	29-Oct-2020

Complete List of Authors:	Richards, David; University of Exeter, Institute for Health Research; Western Norway University of Applied Sciences Department of Health and Caring Sciences<br>Rose Sugg, Holly; University of Exeter Medical School, University of Exeter Medical School<br>Cockcroft, Emma; University of Exeter, Institute for Health Research<br>Cooper, Joanne; Notttingham University Hospitals NHS Trust, Corporate; University of Nottingham, School of Health Sciences<br>Cruickshank, Susanne; Royal Marsden NHS Foundation Trust<br>Doris, Faye; University of Exeter, Institute for Health Research<br>Hulme, Claire; University of Exeter, Health Economics Group<br>Logan, Phillipa; The University of Nottingham, Community Health Sciences<br>Iles-Smith, Heather; University of Salford<br>Melendez-Torres, G.J; University of Exeter,<br>Rafferty, Anne Marie; King's College London, Florence Nightingale School of Nursing and Midwifery<br>Reed, Nigel; University of Exeter, Institute for Health Research<br>Russell, Anne-Marie; University of Exeter, School of Nursing<br>Shepherd, Maggie; University of Exeter, NIHR Clinical Research Facility<br>Singh, Sally; University Hospitals of Leicester NHS Trust, Cardiac/Pulmonary Rehabilitation<br>Thompson Coon, Jo; University of Exeter Medical School, NIHR CLAHRC South West Peninsula<br>Tooze, Susannah; University of Exeter, Academy of Nursing<br>Wootton, Stephen; University of Southampton, Insitute of Human Nutrition<br>Abbott, Rebecca; University of Exeter Medical School, NIHR CLAHRC South West Peninsula<br>Bethel, Alison; University of Exeter, PenCLAHRC<br>Creanor, Siobhan; University of Exeter<br>Quinn, Lynne; University of Exeter, Clinical Trials Unit<br>Tripp, Harry; University of Exeter, Clinical Trials Unit<br>Warren, Fiona; University of Exeter Medical School, Institute of Health Research

	Whear, Rebecca; University of Exeter<br>Bollen, Jessica; University of Exeter, Clinical Trials Unit<br>Hunt, Harriet; University of Exeter Medical School, Exeter Medical School<br>Kent, Merryn; University of Exeter, Institute for Health Research<br>Morgan, Leila; Royal Devon and Exeter NHS Foundation Trust<br>Morley, Naomi; University of Exeter,<br>Romanczuk, Lidia; University of Exeter, Institute for Health Research

Keywords:	COVID-19, INFECTIOUS DISEASES, Protocols & guidelines < HEALTH SERVICES ADMINISTRATION & MANAGEMENT, Clinical trials < THERAPEUTICS

SCHOLARONE™
Manuscripts

I, the Submitting Author has the right to grant and does grant on behalf of all authors of the Work (as defined in the below author licence), an exclusive licence and/or a non-exclusive licence for contributions from authors who are: i) UK Crown employees; ii) where BMJ has agreed a CC-BY licence shall apply, and/or iii) in accordance with the terms applicable for US Federal Government officers or employees acting as part of their official duties; on a worldwide, perpetual, irrevocable, royalty-free basis to BMJ Publishing Group Ltd ("BMJ") its licensees and where the relevant Journal is co-owned by BMJ to the co-owners of the Journal, to publish the Work in this journal and any other BMJ products and to exploit all rights, as set out in our licence.

The Submitting Author accepts and understands that any supply made under these terms is made by BMJ to the Submitting Author unless you are acting as an employee on behalf of your employer or a postgraduate student of an affiliated institution which is paying any applicable article publishing charge ("APC") for Open Access articles. Where the Submitting Author wishes to make the Work available on an Open Access basis (and intends to pay the relevant APC), the terms of reuse of such Open Access shall be governed by a Creative Commons licence – details of these licences and which Creative Commons licence will apply to this Work are set out in our licence referred to above.

Other than as permitted in any relevant BMJ Author's Self Archiving Policies, I confirm this Work has not been accepted for publication elsewhere, is not being considered for publication elsewhere and does not duplicate material already published. I confirm all authors consent to publication of this Work and authorise the granting of this licence.

For peer review only

COVID-NURSE: development and evaluation of the effects of a COVID-specific fundamental nursing care protocol compared to care as usual on experience of care for non-invasively ventilated patients in hospital with the SARS-CoV-2 virus: protocol for a cluster randomised controlled trial

David A. Richards, Holly V.R. Sugg, Emma Cockcroft, Joanne Cooper, Susan Cruickshank, Faye Doris, Claire Hulme, Phillipa Logan, Heather Iles-Smith, G.J. Melendez-Torres, Anne Marie Rafferty, Nigel Reed, Anne-Marie Russell, Maggie Shepherd, Sally J. Singh, Jo Thompson Coon, Susannah Tooze, Stephen Wootton, Rebecca Abbott, Alison Bethel, Siobhan Creanor, Lynne Quinn, Harry Tripp, Fiona C. Warren, Rebecca Whear, Jessica Bollen, Harriet A. Hunt, Merryn Kent, Leila Morgan, Naomi Morley, Lidia Romanczuk

Corresponding author

David A. Richards
University of Exeter
St Luke's Campus
Heavitree Road
Exeter EX1 2LU
United Kingdom

David A. Richards, University of Exeter, Exeter, UK; and Western Norway University of Applied Sciences, Bergen, Norway

Holly V. R. Sugg, University of Exeter, Exeter. UK

Emma Cockcroft, University of Exeter, Exeter, UK

Joanne Cooper, Nottingham University Hospitals NHS Trust, Nottingham, UK

Susan Cruickshank, Royal Marsden NHS Foundation Trust, London, UK

Faye Doris, University of Exeter, Exeter, UK

Claire Hulme, University of Exeter, Exeter, UK

Phillipa Logan, University of Nottingham, Nottingham, UK

Heather Iles-Smith, University of Salford, Salford, UK

G.J. Melendez-Torres, University of Exeter, Exeter, UK

Anne Marie Rafferty, King's College, London, UK

Nigel Reed, University of Exeter, Exeter, UK

Anne-Marie Russell, University of Exeter, Exeter, UK

Maggie Shepherd, University of Exeter, Exeter, UK

For peer review only

Sally J. Singh, University Hospitals of Leicester NHS Trust, Leicester, UK

Jo Thompson Coon, University of Exeter, Exeter, UK

Susannah Tooze, University of Exeter, Exeter, UK

Stephen Wotton, University of Southampton, Southampton. UK

Rebecca Abbott, University of Exeter, Exeter, UK

Alison Bethel, University of Exeter, Exeter, UK

Siobhan Creanor, University of Exeter, Exeter, UK

Lynne Quinn, University of Exeter, Exeter, UK

Harry Tripp, University of Exeter, Exeter, UK

Fiona C. Warren, University of Exeter, Exeter, UK

Rebecca Whear, University of Exeter, Exeter, UK

Jessica Bollen, University of Exeter, Exeter, UK

Harriet A. Hunt, University of Exeter, Exeter, UK

Merryn Kent, University of Exeter, Exeter, UK

Leila Morgan, Royal Devon and Exeter NHS Foundation Trust, Exeter, UK

Naomi Morley, University of Exeter, Exeter, UK

Lidia Romanczuk, University of Exeter, Exeter, UK

Keywords: Nursing, Covid-19, Infectious Diseases, Protocols and Guidelines (Health Services Administration & Management), Clinical Trials (Therapeutics)

Word count 3340

For peer review only

COVID-NURSE: development and evaluation of the effects of a COVID-specific fundamental nursing care protocol compared to care as usual on experience of care for non-invasively ventilated patients in hospital with the SARS-CoV-2 virus: protocol for a cluster randomised controlled trial

Protocol Version 2 12/10/2020

Funder: NIHR/UKRI/MRC

Study Grant Ref: MR/V02776X/1

Trial Registration: ISRCTN13177364

Ethics approval: IRAS ID 288479; REC reference: 20/NE/0253

Abstract:

Introduction; Patient experience of nursing care is correlated with safety, clinical effectiveness, care quality, treatment outcomes and service use. Effective nursing care includes nurse-patient relationships and transactional behaviours to meet patients' fundamental care needs. The high risk of transmission of the SARS-CoV-2 virus compromises nurses' relational and transactional care. No evidence-based nursing guidelines exist for patients with SARS-CoV-2, leading to potential variations in patient experience, outcomes, quality and costs.

Methods and analysis; we aim to recruit 1080 in-patient participants treated for the SARS-CoV-2 virus from 18 UK hospitals, to a three wave, rapid-cycle cluster randomised controlled trial, with embedded process and economic evaluations, of a fundamental nursing care protocol co-designed by nurses and patients, using data from a systematic review and survey of nursing innovations, compared to care as usual. Our co-primary outcomes are patient-reported experience (Quality from the Patients' Perspective; Relational Aspects of Care Questionnaire); secondary outcomes include care quality (pressure sores, falls, medication errors); functional ability (Barthell Index); treatment outcomes (WHO Clinical Progression Scale); depression (PHQ-2), anxiety (GAD-2), health utility (EQ5D), and nurse-reported outcomes (Measure of Moral Distress for Health Care Professionals). We will estimate mean differences or risk ratios comparing intervention and control hospitals using paired-tests on cluster-level summaries weighted by the number of observations in each cluster, also estimating intervention effectiveness in an exploratory unpaired analysis using generalised linear mixed-effects models to model time trends, including time from implementation. We will undertake a planned sub-group analysis to compare the impact of ethnicity on outcomes.

For peer review only - http://bmjopen.bmj.com/site/about/guidelines.xhtml

Ethics and dissemination; research governance and ethical approvals are from the UK National Health Service Health Research Authority and Research Ethics Service. Dissemination will be open access through peer reviewed scientific journals, study website, press and online media, including free online training materials on the Open University's FutureLearn web platform.

Strengths and limitations of this study

- This study is the first randomised controlled trial of a fundamental care clinical nursing protocol for patients with the SARS-CoV-2 virus
- The intervention relates specifically to patients with the SARS-CoV-2 virus admitted to inpatient wards who are not invasively ventilated
- The trial will be conducted in the UK only
- The intervention programme theory will enable generalisation of our findings to other environments such as care homes, patients with other conditions requiring isolation and global health systems

Background

Patient experience of nursing care is correlated with safety, clinical effectiveness, care quality, treatment outcomes including mortality and overall service use.(1-7) Nursing care is a key determinant of patient experience(8, 9) and satisfaction.(10) Effective nursing care requires the establishment of compassionate nurse-patient relationships known as 'relational nursing care'. This facilitates nurses' 'transactional care' behaviours (the physical work undertaken by nurses) to meet the fundamental care needs of patients such as nutrition, hydration, skin integrity, personal care, mobility, hygiene, breathing, elimination and mental wellbeing.(11, 12)

Measuring patient experience is analogous to collecting a patient-reported outcome measure (PROM)(13) in that both are "*a measurement based on a report that comes directly from the patient (i.e., study subject*)".(14) However, whereas a PROM captures "*the status of a patient's health condition without amendment or interpretation of the patient's response by a clinician or anyone else*",(14) a patient-reported *experience* measure (PREM) is a measure of a patient's perception of their personal experience of the healthcare they have received.(15) Two PREMs for evaluating patients' experiences of transactional and relational nursing have been developed and tested; for transactional nursing, the Relational Aspects of Care Questionnaire – RACQ),(8, 16) and for relational nursing the Quality from the Patient's Perspective – QPP.(17, 18)

The combination of COVID-19 symptoms and high risk of transmission of the SARS-CoV-2 virus poses unique challenges for both transactional and relational nursing care. Previously, the 2003 SARS

For peer review only - http://bmjopen.bmj.com/site/about/guidelines.xhtml

outbreak demonstrated that nurses' ability to provide compassionate fundamental care was compromised: "*The establishment/maintenance of therapeutic nurse-client relationship required additional time given the barriers of mask, gloves and gowns*".p4(19) "*Restrictions on visitors were difficult for staff because family members are usually involved in the social, psychological and, to some extent, physical care of patients*".p6(20) "*Interaction time decreased, and patients began to feel more abandoned*".p28(21)

Currently, no international evidence-based guidelines exist for nursing hospitalised patients with SARS-CoV-2, the majority of whom are not invasively mechanically ventilated, leading to potential variations in patient experience, treatment outcomes, care quality and costs. Although individual nurses, teams and organisations have rapidly adapted their procedures to nurse these patients, compared to usual nursing care, we do not know how to optimise nursing care for the specific challenges of nursing COVID-19 patients, including mitigating the impact of wearing personal protective equipment, nor how effective these adaptations are in terms of these important outcomes.

Between July and October 2020, we developed a clinical nursing protocol through: 1) a rapid review(22) of published literature to identify the evidence for the effectiveness of and barriers to fundamental nursing care procedures in patients with SARS-CoV-2 virus or other conditions requiring isolation, in terms of overall patient experience, care quality, functional ability and treatment outcomes – PROSPERO registration number: CRD42020200914;(23) 2) a UK-wide survey of nurses and non-registered care staff working with hospitalised patients with the SARS-CoV-2 virus who are not invasively ventilated to identify respondents' views on missed care,(24, 25) barriers to care, and innovation strategies respondents have adopted to meet the fundamental care needs of such patient, governance approval from the Health Research Authority (IRAS project ID: 287288, protocol number 1920/31) and ethical approval from the University of Exeter research ethics committee (no: 20/07/256); 3) consensus development panels(26-28) of nurses, and patients with experience of hospitalisation with the SARS-CoV-2 virus, to agree the content and design of the clinical nursing protocol.

In the COVID-NURSE trial, our objective is to compare this evidence-based nursing protocol for patients with the SARS-CoV-2 virus against care as usual, in terms of patients' reported experience of transactional and relational nursing care, care quality, treatment outcomes and costs.

Methods: Participants, Interventions, and Outcomes

For peer review only - http://bmjopen.bmj.com/site/about/guidelines.xhtml

Design: we will undertake a rapid-cycle cluster randomised controlled superiority trial(29-31) with three waves of analysis, and embedded process(32) and economic evaluations.(33) (02/11/2020 – 29/03/2021)

Setting: we will recruit 18 UK district-general and teaching hospital NHS Trusts (clusters), representing a spread of hospital type, size and ethnic diversity of admitted patients.

Participant eligibility criteria: patients who are not invasively ventilated, aged ≥18 years, currently hospitalised and treated for infection with the SARS-CoV-2 virus and who have received nursing care for at least 72 hours during their current admission; registered nurses and nursing care workers caring for patients hospitalised and being treated for infection with the SARS-CoV-2 virus. Participants must be able to give informed consent. We will provide translation facilities for participants unable to understand and speak English.

Interventions:

Experimental Intervention: care as usual plus the clinical protocol developed as described above. The protocol consists of four elements adapted from the methods used successfully in a previous cluster randomised controlled trial to significantly increase handwashing by nurses and care staff(34): 1) a guideline outlining strategies to overcome the barriers to care identified in our review, survey and consensus panels; 2) a staff education programme hosted on the Open University's 'FutureLearn' platform,(35) consisting of videos, written information and exercises to address barriers and nursing strategies; 3) reminders in the form of posters with key information on the same strategies to overcome barriers to care; 4) leadership from ward managers and senior nurses, to address the organisation of care, education for nursing teams and support for nurses caring for patients hospitalised with the SARS-CoV-2 virus, information on this delivered via the 'FutureLearn' platform.(35)

After each wave of implementation and drawing on process evaluation findings, we will modify our clinical protocol accordingly and embed the new version of the protocol in the subsequent experimental intervention cycle site. We will monitor clinical protocol adherence and fidelity as part of the process evaluation, amending our guideline, educational and leadership strategies accordingly before each subsequent rapid cycle wave.

Control: care as usual only. We will record staffing staff skill mix details alongside a description of clinical nursing procedures and organisation in place in the usual care clusters.

Outcomes: we will collect participant-level outcome data. Our co-primary outcomes will be patient-reported experience measures addressing transactional nursing care using the QPP(17, 18) and

For peer review only - http://bmjopen.bmj.com/site/about/guidelines.xhtml

relational nursing care using the RACQ.(8, 16) Our secondary outcomes will be: three elements of ward based care quality collected by the United Kingdom National Health Service Ward to Board dashboard(36) – prevalence of level 3 and 4 pressure sores, frequency of patient falls, frequency of medication errors; functional ability by the Barthel Index;(37, 38) treatment outcomes by the WHO Clinical Progression Scale;(39) depression by the PHQ-2,(40), anxiety by the GAD-2,(41) and nurse outcomes by the Measure of Moral Distress for Health Care Professionals.(42, 43)

Participant timeline: we will undertake the trial during 20 weeks of intervention and rolling data collection, with data collected from patient participants admitted for treatment of SARS-CoV-2 infection and staff caring for them. The cluster sites will participate in one of three cycles; we will initiate the cycles at six weekly intervals. Each cycle will run for six weeks.

Target sample size: using published formulae,(30) we have calculated our sample size based on an estimate of the minimum clinically important difference for the QPP,(18) and the typical within-unit standard deviation (0.6) supplied by measure developers.(44) We have examined a number of potential scenarios taking into account different cluster sizes and different between-cluster standard deviation values informed by between-country differences on measures,(18) estimated to provide 90% and 80% power (Table 1).

We have selected 90% power and a participant cluster size of 60 based on feasibility conversations with NHS clinical leaders. As such, we will need to recruit a total of nine clusters per allocated arm (18 in total) to detect a between-group standard deviation of 0.082 in the QPP with a two-sided significance level of 0.05. Were we to have a recruitment shortfall of 33% per cluster, a total of 10.2 clusters per arm would be required for 90% power. However, with the originally planned number of clusters, we will still attain 80% power.

Recruitment: we will recruit cluster sites via the NHS chief nurse and clinical research networks (CRN). During stage 1 of the study, we will monitor available data from Public Health England on case and hospitalisation rates by NHS Trust in order to identify recruitment sites where there are sufficient potential participants. We will use this data to prepare sites for involvement. In order to maximise participant numbers, we will recruit sites as near as possible in time to the randomisation point for that rapid cycle, when sufficient participants are likely to be available for our sample. Clinical research nurses will recruit patient participants from admitted patients according to the eligibility criteria above.

Allocation: we will pair the 18 sites according to hospital type (2 categories: research intensive vs not research intensive, where research intensive is defined as ≥0.1 unique recruiting studies in the 2019-2020 Q4 open data from NIHR divided by number of inpatient elective admissions in Jan-Mar

For peer review only

2020, and non-research intensive is <0.1) and patient ethnic diversity according to the latest census data for the local authority in which a hospital is located (Low (<68%) vs Medium (68% to 74%) vs High (>74%) non-BAME categories based around an overall mean of 71% non-BAME admissions, due to these variables potential impact on outcomes.(45) For each of the nine pairs we will randomly allocate one site to the intervention group and one site to the control group. We will conduct allocation over three cycles of six sites (three pairs) each. To ensure allocation concealment, we will undertake randomisation of matched pairs through the use of an externally administered, password-protected randomisation website independently developed and maintained by the UKCRC-registered University of Exeter Clinical Trials Unit.

Data collection: we will collect outcome data from patient participants after they have received nursing care for a minimum of 72 hours. All outcome measures will be applied to all participants equally and collected by clinical research nurses. It will not be possible to blind research nurses, clinicians or patient participants to allocation. Statistical analysts will be blind to group allocation when receiving data to analyse. We will collect outcome data either face to face or by video/audio technology according to the infection control procedures in place and participant preference. We will collect outcome data from nurses either face to face in their hospital, or over the phone, according to their preference at the end of each rapid cycle wave. Site level *Ward to Board* data will be collected from routine hospital statistics.

Data management: participants will be identified by a unique study ID**.** Personal identifiable data including date of birth and NHS number, may be collected but will be stored separately to research data and will be destroyed as per applicable regulations when the project is concluded. Data will be managed by the UKCRC registered Exeter Clinical Trials Unit (ExeCTU) following GDPR and data protection guidelines and all relevant Clinical Trial Regulations. All data will be anonymised prior to publication. Data will be collected and stored electronically in accordance with the Data Protection Act 2018 and ICH GCP E6 R2. ExeCTU will use Redcap Cloud Electronic Data Capture System to collect all case report data. This system is validated to ISO27001 standards, backed up and maintained in Europe. This system is fully compliant to GDPR regulations and managed by ExeCTU. Any additional study data will be stored and backed up on the secure ExeCTU servers, maintained by ExeCTU. Data will be cleaned and validated appropriately and a full Data Protection Impact Assessment (DPIA) will be undertaken along with the development of a comprehensive Data Management Plan (DMP) before the first participant is recruited.

Where data are disseminated (e.g. via report, presentation or publication), they will be anonymised. We will align all confidentiality and data handling with the Caldicott Principles. Anonymised data will be stored indefinitely on a research data storage system provided by the University of Exeter called

For peer review only - http://bmjopen.bmj.com/site/about/guidelines.xhtml

Open access Research Exeter (ORE) for archiving (http://www.exeter.ac.uk/research/openresearch/policies/ore/).

Statistical data analysis: we will analyse numerical data in Stata v.16 (46). Given the small cluster numbers, our primary analysis will estimate mean differences or risk ratios as appropriate for the outcome under consideration, using paired-tests on cluster-level summaries weighted by the number of observations in each cluster, to compare intervention and control groups. Secondly, and focusing on patient-level outcomes, we will estimate intervention effectiveness in an exploratory unpaired analysis. This analysis will use generalised linear mixed-effects models to model time trends. Analysis will include time from implementation, reflecting growing adaptation and expertise, as well as calendar time, reflecting the course of the SARS-COV-2 epidemic. We will also undertake a planned sub-group analysis to compare the impact of ethnicity on our primary and secondary outcomes. Because this trial was a combined effectiveness/implementation design, we have not specified a hierarchy of outcomes, or propose to correct for multiple comparisons. All analyses are pre-specified and use a nominal significance level of 0.05 with two-sided tests.

Process evaluation: we will evaluate the impact of nurses' and care staff members' fidelity to the clinical protocol, intervention mechanisms and context of care delivery on outcomes using: a bespoke instrument for capturing fidelity to reflect the protocol components; the Culture of Care Barometer Questionnaire;(47) the NoMAD instrument for measuring implementation readiness and acceptability;(48, 49) contextual data on patient acuity, staffing ratios, nurses/care staff education levels, seniority and year of qualification, and other contextual variables. During cycles 1 and 2, we will conduct an interim analysis of process evaluation data on fidelity to the protocol, and protocol utilisation to evaluate intervention component fidelity and implementation. We will subsequently amend the clinical protocol based on this data before embedding the new protocol in intervention sites.

We will conduct semi-structured qualitative interviews with a purposive sample of nurses and care staff, using a topic guide derived from the content of the clinical protocol and our analysis of quantitative data, to explore their views on the mechanisms, impact and acceptability of the clinical protocol. This method will enable us to investigate the meaning of participants' responses, both exploring views on our predefined topics of interest and eliciting more detail on any emerging themes.(50) Interviews will be conducted using telephone or online audio-conference methods, unless face to face interviews are risk assessed as being safe and are preferred by participants. With participants' consent, we will audio-record interviews and transcribe interviews verbatim. We will use NVivo10(51) to organise the data and analyse data using Framework analysis to allow for the combination of inductive and deductive approaches in our development of themes.(52) We will use

For peer review only - http://bmjopen.bmj.com/site/about/guidelines.xhtml

joint displays(53) to integrate the qualitative data with the quantitative outcome data in a mixed methods analysis(54) to explore and explain variation in the outcome data.

Economic evaluation: we will conduct within trial cost effectiveness analysis by adopting the NHS perspective with the main outcome measure the Quality-adjusted life year (QALY) derived from the EQ-5D-5L(33, 55) (baseline and discharge). We will apply the NICE threshold of £20,000 per QALY. Cost data will include staff training, length of stay, including ICU costs and COVID-specific interventions from baseline to discharge. We will apply unit costs derived from national sources including Personal Social Services Research Unit (56) and NHS Reference Costs.(57) Non-parametric bootstrapping will be used to quantify uncertainty. We will present outputs as ICERs, cost effectiveness acceptability curves and expected net benefit.

Monitoring: day to day management of the trial and study will be by the chief-investigator, other co-investigators and a research manager. We will set up an independent combined Trial Steering and Data Monitoring Committee (TSC/DMC)(58) and a Patient and Public Involvement group.(59) We will undertake a risk assessment of the trial and produce a monitoring plan that will be commensurate with the risk identified to particularly ensure that we have input from people of colour, including from Black, Asian and other minority ethnic groups, who are at greater risk from infection with the SARS-CoV-2 virus.

Safety reports of serious adverse events (SAE)

Fatal and life-threatening events (otherwise known as Serious Adverse Events) experienced by participants while participating in the study are unlikely to be caused by participation in this trial. The recording and reporting of non-serious AEs in this study is not required. Each serious adverse event will be reviewed by the CI on a case-by-case basis, and only reported as an SAE if deemed relevant.

Patient and Public Involvement: The proposed study has been informed by ongoing collaborations between patients, clinicians and researchers, including in a previous NIHR-funded project (ESSENCE) on fundamental nursing care. A version of the lay summary was reviewed by six members of the 'Peninsula Patient Involvement Group', giving feedback on the broad idea of the research as well as specific points on the clarity of the summary. A patient co-applicant has been involved with the development of the proposal. We will ensure there is a patient voice in all discussions and decisions, and will ensure the integration of broader PPI activity in these decisions. We have worked with a wider group of patients with experience of hospitalisation for COVID-19 to inform the co-production of the nursing protocol, development of all patient facing materials, finalising the nursing protocol for rapid-cycle testing, adaptions to the nursing protocol after each cycle of testing, dissemination of

For peer review only

findings, including the production of an online training module for nurses on the FutureLearn platform. The involvement of patients will be informed by what is possible within the ongoing pandemic, and to suit the situation of interested patients. Involvement will be flexible and responsive to needs. We will adopt high standards of equality, diversity and inclusivity(60) throughout.

Ethical issues: we will conduct this trial in such a way as to protect the human rights and dignity of the participants, as reflected in the Helsinki Declaration.(61) We have obtained governance and ethical approval from the Health Research Authority National Research Ethics Service (IRAS project ID 288479; REC reference: 20/NE/0253). Participants will not receive any financial inducement to participate. We will invite eligible patients and nurses to participate in data collection and will only collect data from participants that individually consent. Participants may withdraw from the study at any time without prejudicing their treatment, care or employment. We will conform to Good Clinical Practice Guidelines, data protection and freedom of information acts. All participant and research data will be stored securely and anonymised wherever possible. All identifiable participant information will be stored separately to questionnaire data which will be coded by an ID number only. No published material will contain identifiable participant information.

Outputs and dissemination: we will disseminate our results through publication in peer reviewed scientific journals, our study website and other press and online media. Manuscript authors will be those considered to have made a substantive intellectual contribution to the study. The investigators and relevant authorities will have access to the trial dataset. Furthermore, we will store anonymised research data and outputs in the University of Exeter's Open Research Exeter repository (https://ore.exeter.ac.uk/repository/) in order to facilitate open access to, and the impact of, our research.

The main output from this study will be evidence on whether our COVID-specific clinical nursing care protocol delivers additional benefits in overall patient experience, care quality, functional ability and treatment outcomes, with cost-effectiveness estimates, for hospitalised patients with the SARS-CoV-2 virus not invasively ventilated, compared to care as usual in hospital wards. We will have a programme theory allowing us to potentially generalise any such benefits to environments like care homes, patients with other conditions requiring isolation and to global health systems. If effective, our guidelines, education materials and strategies will be made accessible via Health Education England, the Open University's FutureLearn(35) web platform, and through experienced NHS sites acting as training and dissemination hubs for other health Trusts, environments such as care homes, and global health systems.

For peer review only - http://bmjopen.bmj.com/site/about/guidelines.xhtml

Discussion

Our research aligns with the WHO COVID-19 R&D Roadmap priorities and research gaps, viz: 'to determine optimal clinical practice strategies to improve the processes of care'. It includes 'rapid approaches to capture healthcare worker views (surveys, interviews)' and a rapid ethnograph[y] in healthcare'.(62). It will, therefore, provide guidance to nurses attempting to overcome significant barriers to providing fundamental transactional and relational care to patients; specifically those with the SARS-CoV-2 virus not invasively ventilated but with a generalisable programme theory suitable for adaptation to other care environments, other pandemics and other nations globally.

Authors' contributions: DR wrote the protocol with input from co-investigators HVRS, EC, JC, SC, CH, PL, HI-S, GJM-T, NR, AMRa, AMRu, MS, SJS, J, T-C, ST and SW. All other authors (RA, AB, SC, FD, LQ, HT, FW, RW, JB, HAH, KM, LM, NM and LR) reviewed the manuscript and made editorial suggestions. FD and NR are independent patient and public Involvement members of the team, GJM-T is the senior trial statistician and CH the trial economist.

Funding statement: 'This work is supported by National Institute for Health Research and UK Research and Innovation, administered by the MRC: grant number MR/V02776X/1. The Trial Sponsor is the University of Exeter, ref: 1920/ Research Ethics and Governance Office, Lafrowda House, St Germans Road, Exeter, Devon, EX4 6TL. Neither the funder nor sponsor will have any role or ultimate authority in study design; collection, management, analysis, and interpretation of data; writing of the report; and the decision to submit the report for publication.

Competing interests: the authors confirm that they have no conflict of interests to declare

For peer review only

		90% power			80% power		
		Cluster size and number per trial arm			Cluster size and number per trial arm		
		40	60	80	40	60	80
Between-group standard deviation	0.06	8.6	7.0	6.3	6.9	5.8	5.2
	0.082	10.2	8.7	7.9	8.2	7.0	6.4
	0.10	12.00	10.4	9.6	9.5	8.3	7.7

Table 1 Sample size calculation

For peer review only

1. Darzi A. Quality and the NHS next stage review. Lancet. 2008;371(9624):1563-4.
2. Vincent C, Amalberti R. Safer Healthcare: Strategies for the Real World. Cham (CH)2016.
3. Black N, Varaganum M, Hutchings A. Relationship between patient reported experience (PREMs) and patient reported outcomes (PROMs) in elective surgery. Bmj Qual Saf. 2014;23(7):534-42.
4. Doyle C, Lennox L, Bell D. A systematic review of evidence on the links between patient experience and clinical safety and effectiveness. BMJ Open. 2013;3(1).
5. Department of Health. Report of the Mid Staffordshire NHS Foundation Trust Public Enquiry. London: Department of Health; 2013.
6. @BorisJohnson. It is hard to find the words to express my debt to the NHS for saving my life. The efforts of millions of people across this country to stay home are worth it. Together we will overcome this challenge, as we have overcome so many challenges in the past. #StayHomeSaveLives [twitter post]12/04/2020 10/05/2020.
7. Aiken LH, Sloane DM, Bruyneel L, Van den Heede K, Griffiths P, Busse R, et al. Nurse staffing and education and hospital mortality in nine European countries: a retrospective observational study. Lancet. 2014;383(9931):1824-30.
8. Graham C, Kasbauer S, Cooper R, King J, Sizmur S, Jenkinson C, et al. An evaluation of a near real-time survey for improving patients' experiences of the relational aspects of care: a mixed-methods evaluation. Health Services and Delivery Research. Southampton (UK)2018.
9. Murrells T, Robert G, Adams M, Morrow E, Maben J. Measuring relational aspects of hospital care in England with the 'Patient Evaluation of Emotional Care during Hospitalisation' (PEECH) survey questionnaire. BMJ Open. 2013;3(1).
10. Aiken LH, Sloane D, Griffiths P, Rafferty AM, Bruyneel L, McHugh M, et al. Nursing skill mix in European hospitals: cross-sectional study of the association with mortality, patient ratings, and quality of care. Bmj Qual Saf. 2017;26(7):559-68.

11. Feo R, Conroy T, Jangland E, Muntlin Athlin A, Brovall M, Parr J, et al. Towards a standardised definition for fundamental care: A modified Delphi study. J Clin Nurs. 2018;27(11-12):2285-99.
12. Kitson A, Conroy T, Wengstrom Y, Profetto-McGrath J, Robertson-Malt S. Defining the fundamentals of care. Int J Nurs Pract. 2010;16(4):423-34.
13. Calvert MJ, Freemantle N. Use of health-related quality of life in prescribing research. Part 1: why evaluate health-related quality of life? J Clin Pharm Ther. 2003;28(6):513-21.
14. FDA-NIH Biomarker Working Group. BEST (Biomarkers, EndpointS, and other Tools) Resource Silver Spring and Bethesda (MD): Food and Drug Administration (US) and National Institutes of Health (US); 2016 [updated 2020. Available from: https://www.ncbi.nlm.nih.gov/books/NBK326791/.
15. Coulter A. Measuring what matters to patients. BMJ. 2017;356:j816.
16. Kelly L, Sizmur S, Kasbauer S, King J, Cooper R, Jenkinson C, et al. The Relational Aspects of Care Questionnaire: item reduction and scoring using inpatient and accident and emergency data in England. Patient Relat Outcome Meas. 2018;9:173-81.
17. Wilde Larsson B, Larsson G. Development of a short form of the Quality from the Patient's Perspective (QPP) questionnaire. J Clin Nurs. 2002;11(5):681-7.
18. Larsson B, Larsson G, Chantereau M, von Holstein K. International comparisons of patients' views on quality of care. Int J Health Care Qual Assur Inc Leadersh Health Serv. 2005;18(1):62-73.
19. Canadian Nurses Association. CNA Brief to the National Advisory Committee on SARS and Public Health: Lessons learned and recommendations. Ottawa, Canada: Canadian Nurses Association; 2003.
20. Baumann A, Blythe, J., Underwood, J., Dzuiba, J.,. Capacity, casualization, and continuity: the impact of SARS Toronto, Canada: University of Toronto, Faculty of Nursing and McMaster University, School of Nursing; 2003.
21. Registered Nurses Association of Ontario. SARS Unmasked: Celebrating Resilience, Exposing Vulnerability. A Report on the Nursing Experience with SARS in Ontario. Toronto: Registered Nurses Association of Ontario; 2003.
22. Dobbins M. Rapid review guidebook. Hamilton, ON: National Collaborating Centre for Methods and Tools; 2017.
23. Effectiveness of and barriers to fundamental nursing care procedures in patients with SARS-COV-2 virus: a rapid systematic review. [Internet]. PROSPERO 2020 CRD42020200914. 2020 [cited 30/07/2020]. Available from: https://www.crd.york.ac.uk/prospero/display_record.php?ID=CRD42020200914.
24. Ball JE, Bruyneel L, Aiken LH, Sermeus W, Sloane DM, Rafferty AM, et al. Post-operative mortality, missed care and nurse staffing in nine countries: A cross-sectional study. Int J Nurs Stud. 2018;78:10-5.
25. Ball JE, Murrells T, Rafferty AM, Morrow E, Griffiths P. 'Care left undone' during nursing shifts: associations with workload and perceived quality of care. Bmj Qual Saf. 2014;23(2):116-25.
26. Halcomb E, Davidson P, Hardaker L. Using the consensus development conference method in healthcare research. Nurse Res. 2008;16(1):56-71.
27. Nair R, Aggarwal R, Khanna D. Methods of formal consensus in classification/diagnostic criteria and guideline development. Semin Arthritis Rheum. 2011;41(2):95-105.

For peer review only

28. Waggoner J, Carline JD, Durning SJ. Is There a Consensus on Consensus Methodology? Descriptions and Recommendations for Future Consensus Research. Acad Med. 2016;91(5):663-8.
29. Johnson K, Gustafson D, Ewigman B, Provost L, Roper R. Using Rapid-Cycle Research to Reach Goals: Awareness, Assessment, Adaptation, Acceleration. AHRQ Publication No. 15-0036. Rockville (MD): Agency for Healthcare Research and Quality; 2015.
30. Hayes RJ, Moulton LH. Cluster Randomised Trials 2nd Edition. Florida: Chapman and Hall/CRC; 2017.
31. Campbell MK, Elbourne DR, Altman DG, Consort Group. CONSORT statement: extension to cluster randomised trials. British Medical Journal. 2004;328(7441):702-8.
32. Moore G, Audrey S, Barker M, Bond L, Bonell C, Cooper C, et al. Process evaluation in complex public health intervention studies: the need for guidance. Journal of Epidemiology and Community Health. 2014;68(2):101-2.
33. National Institute for Health and Care Excellence. Guide to the methods of technology appraisal. London: National Institute for Health and Care Excellence; 2013.
34. Huis A, Schoonhoven L, Grol R, Donders R, Hulscher M, van Achterberg T. Impact of a team and leaders-directed strategy to improve nurses' adherence to hand hygiene guidelines: a cluster randomised trial. Int J Nurs Stud. 2013;50(4):464-74.
35. FutureLearn. London: The Open University and The SEEK Group; [Available from: https://www.futurelearn.com.
36. NHS Improvement. Guide to developing and implementing ward and unit accreditation programmes, IG 09/19. London: NHS Improvement; 2019.
37. Mahoney FI, Barthel DW. Functional Evaluation: The Barthel Index. Md State Med J. 1965;14:61-5.
38. Collin C, Wade DT, Davies S, Horne V. The Barthel ADL Index: a reliability study. Int Disabil Stud. 1988;10(2):61-3.
39. Marshall J, On Behalf Of The Who Working Group On The Clinical Characteristics Of Covid-19 Infection,. A Core Outcome Measure Set for Studies of SARS-CoV2/COVID-19 infection Toronto, Canada: University of Toronto; 2020 [Available from: http://www.comet-initiative.org/Studies/Details/1528.
40. Kroenke K, Spitzer RL, Williams JB. The Patient Health Questionnaire-2: validity of a two-item depression screener. Med Care. 2003;41(11):1284-92.
41. Kroenke K, Spitzer RL, Williams JB, Monahan PO, Lowe B. Anxiety disorders in primary care: prevalence, impairment, comorbidity, and detection. Ann Intern Med. 2007;146(5):317-25.
42. Epstein EG, Whitehead PB, Prompahakul C, Thacker LR, Hamric AB. Enhancing Understanding of Moral Distress: The Measure of Moral Distress for Health Care Professionals. AJOB Empir Bioeth. 2019;10(2):113-24.
43. Corley MC, Elswick RK, Gorman M, Clor T. Development and evaluation of a moral distress scale. J Adv Nurs. 2001;33(2):250-6.
44. Larsson B. estimate of the minimum clinically important difference for the QPP. In: Melendez-Torres G, editor.
45. Downing A, Morris EJ, Corrigan N, Sebag-Montefiore D, Finan PJ, Thomas JD, et al. High hospital research participation and improved colorectal cancer survival outcomes: a population-based study. Gut. 2017;66(1):89-96.
46. Stata Corp. Stata Statistical Software: Release 16. College Station, Texas, USA: Stata Corp LLC; 2019.

For peer review only

47. Rafferty AM, Philippou J, Fitzpatrick JM, Pike G, Ball J. Development and testing of the 'Culture of Care Barometer' (CoCB) in healthcare organisations: a mixed methods study. BMJ Open. 2017;7(8):e016677.
48. Finch TL, Girling M, May CR, Mair FS, Murray E, Treweek S, et al. Improving the normalization of complex interventions: part 2 - validation of the NoMAD instrument for assessing implementation work based on normalization process theory (NPT). BMC Med Res Methodol. 2018;18(1):135.
49. Rapley T, Girling M, Mair FS, Murray E, Treweek S, McColl E, et al. Improving the normalization of complex interventions: part 1 - development of the NoMAD instrument for assessing implementation work based on normalization process theory (NPT). BMC Med Res Methodol. 2018;18(1):133.
50. Gale NK, Heath G, Cameron E, Rashid S, Redwood S. Using the framework method for the analysis of qualitative data in multi-disciplinary health research. BMC Med Res Methodol. 2013;13:117.
51. QSR International. NVivo 10 for windows 2017 [Available from: www.qsrinternational.com/products_nvivo.aspx. .
52. Ritchie J, Spencer L, W OC. Qualitative research practice: A guide for social science students and researchers. London: Sage; 2003.
53. Creswell JW, Plano-Clark VL. Designing and conducting mixed methods research. Thousand Oaks: Sage; 2007.
54. Richards DA, Bazeley P, Borglin G, Craig P, Emsley R, Frost J, et al. Integrating quantitative and qualitative data and findings when undertaking randomised controlled trials. BMJ Open. 2019;9(11):e032081.
55. EuroQol Research Foundation. EQ-5D-5L User Guide. Available from: https://euroqol.org/publications/user-guides2019.
56. Curtis L, Burns A. Unit Costs of Health and Social Care 2019. University of Kent, Canterbury: Personal Social Services Research Unit; 2019.
57. Department of Health. NHS reference costs 2018-2019. London: Department of Health; 2019.
58. Medical Research Council. MRC Guidelines for Good Clinical Practice in Clinical Trials. London: Medical Research Council; 1998.
59. INVOLVE. Briefing notes for researchers: involving the public in NHS, public health and social care research. Eastleigh: INVOLVE; 2012.
60. National Institute for Health Research. Strengthening our commitment to equality, diversity, inclusion and patient and public involvement and engagement (PPIE) 2020 [updated 01/05/2020. Available from: https://www.nihr.ac.uk/documents/strengthening-our-commitment-to-equality-diversity-inclusion-and-patient-and-public-involvement-and-engagement-ppie/24697.
61. World Medical Association. World Medical Association Declaration of Helsinki Ethical Principles for Medical Research Involving Human Subjects. Journal of the American Medical Association. 2013;310(20):2191-4.
62. World Health Organisation. 2019 novel Coronavirus Global research and innovation forum: towards a research roadmap. Geneva: World Health Organisation; 2020.

For peer review only - http://bmjopen.bmj.com/site/about/guidelines.xhtml

SPIRIT

STANDARD PROTOCOL ITEMS: RECOMMENDATIONS FOR INTERVENTIONAL TRIALS

SPIRIT 2013 Checklist: Recommended items to address in a clinical trial protocol and related documents*

**Section/item**	**Item No**	**Description**
**Administrative information**		
Title	1	Descriptive title identifying the study design, population, interventions, and, if applicable, trial acronym P1
Trial registration	2a	Trial identifier and registry name. If not yet registered, name of intended registry P3
	2b	All items from the World Health Organization Trial Registration Data Set Yes
Protocol version	3	Date and version identifier P3
Funding	4	Sources and types of financial, material, and other support P3
Roles and responsibilities	5a	Names, affiliations, and roles of protocol contributors P12
	5b	Name and contact information for the trial sponsor P12
	5c	Role of study sponsor and funders, if any, in study design; collection, management, analysis, and interpretation of data; writing of the report; and the decision to submit the report for publication, including whether they will have ultimate authority over any of these activities P12
	5d	Composition, roles, and responsibilities of the coordinating centre, steering committee, endpoint adjudication committee, data management team, and other individuals or groups overseeing the trial, if applicable (see Item 21a for data monitoring committee) PP10-12
**Introduction**		
Background and rationale	6a	Description of research question and justification for undertaking the trial, including summary of relevant studies (published and unpublished) examining benefits and harms for each intervention P4-5
	6b	Explanation for choice of comparators P6
Objectives	7	Specific objectives or hypotheses P5

For peer review only

Trial design	8	Description of trial design including type of trial (eg, parallel group, crossover, factorial, single group), allocation ratio, and framework (eg, superiority, equivalence, noninferiority, exploratory) P6

Methods: Participants, interventions, and outcomes

Study setting	9	Description of study settings (eg, community clinic, academic hospital) and list of countries where data will be collected. Reference to where list of study sites can be obtained P6
Eligibility criteria	10	Inclusion and exclusion criteria for participants. If applicable, eligibility criteria for study centres and individuals who will perform the interventions (eg, surgeons, psychotherapists) P6
Interventions	11a	Interventions for each group with sufficient detail to allow replication, including how and when they will be administered P6
	11b	Criteria for discontinuing or modifying allocated interventions for a given trial participant (eg, drug dose change in response to harms, participant request, or improving/worsening disease) P10
	11c	Strategies to improve adherence to intervention protocols, and any procedures for monitoring adherence (eg, drug tablet return, laboratory tests) P6
	11d	Relevant concomitant care and interventions that are permitted or prohibited during the trial No restrictions (P6)
Outcomes	12	Primary, secondary, and other outcomes, including the specific measurement variable (eg, systolic blood pressure), analysis metric (eg, change from baseline, final value, time to event), method of aggregation (eg, median, proportion), and time point for each outcome. Explanation of the clinical relevance of chosen efficacy and harm outcomes is strongly recommended P6-7
Participant timeline	13	Time schedule of enrolment, interventions (including any run-ins and washouts), assessments, and visits for participants. A schematic diagram is highly recommended (see Figure) P7
Sample size	14	Estimated number of participants needed to achieve study objectives and how it was determined, including clinical and statistical assumptions supporting any sample size calculations P7
Recruitment	15	Strategies for achieving adequate participant enrolment to reach target sample size P7

Methods: Assignment of interventions (for controlled trials)

Allocation:

For peer review only - http://bmjopen.bmj.com/site/about/guidelines.xhtml

Sequence generation	16a	Method of generating the allocation sequence (eg, computer-generated random numbers), and list of any factors for stratification. To reduce predictability of a random sequence, details of any planned restriction (eg, blocking) should be provided in a separate document that is unavailable to those who enrol participants or assign interventions P7-8
Allocation concealment mechanism	16b	Mechanism of implementing the allocation sequence (eg, central telephone; sequentially numbered, opaque, sealed envelopes), describing any steps to conceal the sequence until interventions are assigned P7-8
Implementation	16c	Who will generate the allocation sequence, who will enrol participants, and who will assign participants to interventions P7-8
Blinding (masking)	17a	Who will be blinded after assignment to interventions (eg, trial participants, care providers, outcome assessors, data analysts), and how P8
	17b	If blinded, circumstances under which unblinding is permissible, and procedure for revealing a participant's allocated intervention during the trial P8
**Methods: Data collection, management, and analysis**		
Data collection methods	18a	Plans for assessment and collection of outcome, baseline, and other trial data, including any related processes to promote data quality (eg, duplicate measurements, training of assessors) and a description of study instruments (eg, questionnaires, laboratory tests) along with their reliability and validity, if known. Reference to where data collection forms can be found, if not in the protocol P8
	18b	Plans to promote participant retention and complete follow-up, including list of any outcome data to be collected for participants who discontinue or deviate from intervention protocols P8
Data management	19	Plans for data entry, coding, security, and storage, including any related processes to promote data quality (eg, double data entry; range checks for data values). Reference to where details of data management procedures can be found, if not in the protocol P8
Statistical methods	20a	Statistical methods for analysing primary and secondary outcomes. Reference to where other details of the statistical analysis plan can be found, if not in the protocol P9
	20b	Methods for any additional analyses (eg, subgroup and adjusted analyses) P9
	20c	Definition of analysis population relating to protocol non-adherence (eg, as randomised analysis), and any statistical methods to handle missing data (eg, multiple imputation) P9

For peer review only - http://bmjopen.bmj.com/site/about/guidelines.xhtml

Methods: Monitoring

Data monitoring	21a	Composition of data monitoring committee (DMC); summary of its role and reporting structure; statement of whether it is independent from the sponsor and competing interests; and reference to where further details about its charter can be found, if not in the protocol. Alternatively, an explanation of why a DMC is not needed P10
	21b	Description of any interim analyses and stopping guidelines, including who will have access to these interim results and make the final decision to terminate the trial PP9-10
Harms	22	Plans for collecting, assessing, reporting, and managing solicited and spontaneously reported adverse events and other unintended effects of trial interventions or trial conduct P10
Auditing	23	Frequency and procedures for auditing trial conduct, if any, and whether the process will be independent from investigators and the sponsor P10

Ethics and dissemination

Research ethics approval	24	Plans for seeking research ethics committee/institutional review board (REC/IRB) approval P11
Protocol amendments	25	Plans for communicating important protocol modifications (eg, changes to eligibility criteria, outcomes, analyses) to relevant parties (eg, investigators, REC/IRBs, trial participants, trial registries, journals, regulators) n/a
Consent or assent	26a	Who will obtain informed consent or assent from potential trial participants or authorised surrogates, and how (see Item 32) P11
	26b	Additional consent provisions for collection and use of participant data and biological specimens in ancillary studies, if applicable n/a
Confidentiality	27	How personal information about potential and enrolled participants will be collected, shared, and maintained in order to protect confidentiality before, during, and after the trial P11
Declaration of interests	28	Financial and other competing interests for principal investigators for the overall trial and each study site P12
Access to data	29	Statement of who will have access to the final trial dataset, and disclosure of contractual agreements that limit such access for investigators P11
Ancillary and post-trial care	30	Provisions, if any, for ancillary and post-trial care, and for compensation to those who suffer harm from trial participation n/a

For peer review only

Dissemination policy	31a	Plans for investigators and sponsor to communicate trial results to participants, healthcare professionals, the public, and other relevant groups (eg, via publication, reporting in results databases, or other data sharing arrangements), including any publication restrictions PP11
	31b	Authorship eligibility guidelines and any intended use of professional writers PP11-12
	31c	Plans, if any, for granting public access to the full protocol, participant-level dataset, and statistical code P11
**Appendices**		
Informed consent materials	32	Model consent form and other related documentation given to participants and authorised surrogates n/a
Biological specimens	33	Plans for collection, laboratory evaluation, and storage of biological specimens for genetic or molecular analysis in the current trial and for future use in ancillary studies, if applicable n/a

*It is strongly recommended that this checklist be read in conjunction with the SPIRIT 2013 Explanation & Elaboration for important clarification on the items. Amendments to the protocol should be tracked and dated. The SPIRIT checklist is copyrighted by the SPIRIT Group under the Creative Commons "Attribution-NonCommercial-NoDerivs 3.0 Unported" license.

For peer review only

BMJ Open

COVID-NURSE: development and evaluation of the effects of a COVID-specific fundamental nursing care protocol compared to care as usual on experience of care for non-invasively ventilated patients in hospital with the SARS-CoV-2 virus: protocol for a cluster randomised controlled trial

Journal:	*BMJ Open*
Manuscript ID	bmjopen-2020-046436.R1
Article Type:	Protocol
Date Submitted by the Author:	03-Mar-2021

Complete List of Authors:	Richards, David; University of Exeter, Institute for Health Research; Western Norway University of Applied Sciences Department of Health and Caring Sciences<br>Rose Sugg, Holly; University of Exeter, Institute for Health Research<br>Cockcroft, Emma; University of Exeter, Institute for Health Research<br>Cooper, Joanne; Nottingham University Hospitals NHS Trust, Corporate; University of Nottingham, School of Health Sciences<br>Cruickshank, Susanne; Royal Marsden NHS Foundation Trust<br>Doris, Faye; University of Exeter, Institute for Health Research<br>Hulme, Claire; University of Exeter, Health Economics Group<br>Logan, Phillipa; University of Nottingham, Community Health Sciences<br>Iles-Smith, Heather; University of Salford<br>Melendez-Torres, G.J; University of Exeter,<br>Rafferty, Anne Marie; King's College London, Florence Nightingale School of Nursing and Midwifery<br>Reed, Nigel; University of Exeter, Institute for Health Research<br>Russell, Anne-Marie; University of Exeter, School of Nursing<br>Shepherd, Maggie; University of Exeter, NIHR Clinical Research Facility<br>Singh, Sally; Leicester Royal Infirmary, Cardiac/Pulmonary Rehabilitation<br>Thompson Coon, Jo; University of Exeter, NIHR CLAHRC South West Peninsula<br>Tooze, Susannah; University of Exeter, Academy of Nursing<br>Wootton, Stephen; University of Southampton, Insitute of Human Nutrition<br>Abbott, Rebecca; University of Exeter, NIHR CLAHRC South West Peninsula<br>Bethel, Alison; University of Exeter, PenARC<br>Creanor, Siobhan; University of Exeter<br>Quinn, Lynne; University of Exeter, Clinical Trials Unit<br>Tripp, Harry; University of Exeter, Clinical Trials Unit<br>Warren, Fiona; University of Exeter, Institute of Health Research<br>Whear, Rebecca; University of Exeter<br>Bollen, Jessica; University of Exeter, Clinical Trials Unit<br>Hunt, Harriet; University of Exeter Medical School, Exeter Medical School<br>Kent, Merryn; University of Exeter, Institute for Health Research

	Morgan, Leila; Royal Devon and Exeter NHS Foundation Trust<br>Morley, Naomi; University of Exeter,<br>Romanczuk, Lidia; University of Exeter, Institute for Health Research
**Primary Subject Heading**:	Nursing
Secondary Subject Heading:	Health services research, Infectious diseases, Respiratory medicine
Keywords:	COVID-19, INFECTIOUS DISEASES, Protocols & guidelines < HEALTH SERVICES ADMINISTRATION & MANAGEMENT, Clinical trials < THERAPEUTICS

SCHOLARONE™ Manuscripts

I, the Submitting Author has the right to grant and does grant on behalf of all authors of the Work (as defined in the below author licence), an exclusive licence and/or a non-exclusive licence for contributions from authors who are: i) UK Crown employees; ii) where BMJ has agreed a CC-BY licence shall apply, and/or iii) in accordance with the terms applicable for US Federal Government officers or employees acting as part of their official duties; on a worldwide, perpetual, irrevocable, royalty-free basis to BMJ Publishing Group Ltd ("BMJ") its licensees and where the relevant Journal is co-owned by BMJ to the co-owners of the Journal, to publish the Work in this journal and any other BMJ products and to exploit all rights, as set out in our licence.

The Submitting Author accepts and understands that any supply made under these terms is made by BMJ to the Submitting Author unless you are acting as an employee on behalf of your employer or a postgraduate student of an affiliated institution which is paying any applicable article publishing charge ("APC") for Open Access articles. Where the Submitting Author wishes to make the Work available on an Open Access basis (and intends to pay the relevant APC), the terms of reuse of such Open Access shall be governed by a Creative Commons licence – details of these licences and which Creative Commons licence will apply to this Work are set out in our licence referred to above.

Other than as permitted in any relevant BMJ Author's Self Archiving Policies, I confirm this Work has not been accepted for publication elsewhere, is not being considered for publication elsewhere and does not duplicate material already published. I confirm all authors consent to publication of this Work and authorise the granting of this licence.

For peer review only

COVID-NURSE: development and evaluation of the effects of a COVID-specific fundamental nursing care protocol compared to care as usual on experience of care for non-invasively ventilated patients in hospital with the SARS-CoV-2 virus: protocol for a cluster randomised controlled trial

David A. Richards, Holly V.R. Sugg, Emma Cockcroft, Joanne Cooper, Susan Cruickshank, Faye Doris, Claire Hulme, Phillipa Logan, Heather Iles-Smith, G.J. Melendez-Torres, Anne Marie Rafferty, Nigel Reed, Anne-Marie Russell, Maggie Shepherd, Sally J. Singh, Jo Thompson Coon, Susannah Tooze, Stephen Wootton, Rebecca Abbott, Alison Bethel, Siobhan Creanor, Lynne Quinn, Harry Tripp, Fiona C. Warren, Rebecca Whear, Jessica Bollen, Harriet A. Hunt, Merryn Kent, Leila Morgan, Naomi Morley, Lidia Romanczuk

Corresponding author

David A. Richards
University of Exeter
St Luke's Campus
Heavitree Road
Exeter EX1 2LU
United Kingdom

d.a.richards@exeter.ac.uk

David A. Richards, University of Exeter, Exeter, UK; and Western Norway University of Applied Sciences, Bergen, Norway

Holly V. R. Sugg, University of Exeter, Exeter. UK

Emma Cockcroft, University of Exeter, Exeter, UK

Joanne Cooper, Nottingham University Hospitals NHS Trust, Nottingham, UK

Susan Cruickshank, Royal Marsden NHS Foundation Trust, London, UK

Faye Doris, University of Exeter, Exeter, UK

Claire Hulme, University of Exeter, Exeter, UK

Phillipa Logan, University of Nottingham, Nottingham, UK

Heather Iles-Smith, University of Salford, Salford, UK

G.J. Melendez-Torres, University of Exeter, Exeter, UK

Anne Marie Rafferty, King's College, London, UK

Nigel Reed, University of Exeter, Exeter, UK

Anne-Marie Russell, University of Exeter, Exeter, UK

For peer review only - http://bmjopen.bmj.com/site/about/guidelines.xhtml

Maggie Shepherd, University of Exeter, Exeter, UK

Sally J. Singh, University Hospitals of Leicester NHS Trust, Leicester, UK

Jo Thompson Coon, University of Exeter, Exeter, UK

Susannah Tooze, University of Exeter, Exeter, UK

Stephen Wotton, University of Southampton, Southampton. UK

Rebecca Abbott, University of Exeter, Exeter, UK

Alison Bethel, University of Exeter, Exeter, UK

Siobhan Creanor, University of Exeter, Exeter, UK

Lynne Quinn, University of Exeter, Exeter, UK

Harry Tripp, University of Exeter, Exeter, UK

Fiona C. Warren, University of Exeter, Exeter, UK

Rebecca Whear, University of Exeter, Exeter, UK

Jessica Bollen, University of Exeter, Exeter, UK

Harriet A. Hunt, University of Exeter, Exeter, UK

Merryn Kent, University of Exeter, Exeter, UK

Leila Morgan, Royal Devon and Exeter NHS Foundation Trust, Exeter, UK

Naomi Morley, University of Exeter, Exeter, UK

Lidia Romanczuk, University of Exeter, Exeter, UK

Keywords: Nursing, Covid-19, Infectious Diseases, Protocols and Guidelines (Health Services Administration & Management), Clinical Trials (Therapeutics)

Word count 4351

For peer review only

COVID-NURSE: development and evaluation of the effects of a COVID-specific fundamental nursing care protocol compared to care as usual on experience of care for non-invasively ventilated patients in hospital with the SARS-CoV-2 virus: protocol for a cluster randomised controlled trial

Protocol Version 5.0 05-02-2021

Funder: NIHR/UKRI/MRC

Study Grant Ref: MR/V02776X/1

Trial Registration: ISRCTN13177364

Ethics approval: IRAS ID 288479; REC reference: 20/NE/0253

Abstract:

Introduction; Patient experience of nursing care is correlated with safety, clinical effectiveness, care quality, treatment outcomes and service use. Effective nursing care includes nurse-patient relationships and transactional behaviours to meet patients' fundamental care needs. The high risk of transmission of the SARS-CoV-2 virus compromises nurses' relational and transactional care. No evidence-based nursing guidelines exist for patients with SARS-CoV-2, leading to potential variations in patient experience, outcomes, quality and costs.

Methods and analysis; we aim to recruit 840 in-patient participants treated for the SARS-CoV-2 virus from 14 UK hospitals, to cluster randomised controlled trial, with embedded process and economic evaluations, of a fundamental nursing care protocol co-designed by nurses and patients, using systematic review and survey data of nursing innovations, compared to care as usual. Our co-primary outcomes are patient-reported experience (Quality from the Patients' Perspective; Relational Aspects of Care Questionnaire); secondary outcomes include care quality (pressure injuries, falls, medication errors); functional ability (Barthell Index); treatment outcomes (WHO Clinical Progression Scale); depression (PHQ-2), anxiety (GAD-2), health utility (EQ5D), and nurse-reported outcomes (Measure of Moral Distress for Health Care Professionals). For our primary analysis we will use a standard generalised linear mixed-effect model adjusting for ethnicity of the patient sample and research intensity at cluster level. We will also undertake a planned sub-group analysis to compare the impact of patient-level ethnicity on our primary and secondary outcomes and will undertake process and economic evaluations.

For peer review only - http://bmjopen.bmj.com/site/about/guidelines.xhtml

Ethics and dissemination; research governance and ethical approvals are from the UK National Health Service Health Research Authority and Research Ethics Service. Dissemination will be open access through peer reviewed scientific journals, study website, press and online media, including free online training materials on the Open University's FutureLearn web platform.

Strengths and limitations of this study

- This study is the first randomised controlled trial of a fundamental care clinical nursing protocol for patients with the SARS-CoV-2 virus
- The intervention relates specifically to patients with the SARS-CoV-2 virus admitted to inpatient wards who are not invasively ventilated
- The intervention programme theory will enable generalisation of our findings to other environments such as care homes, patients with other conditions requiring isolation and global health systems
- The trial co-primary outcomes require patient participants to have capacity to consent and report their experience of care, and therefore we will be unable to collect and report data from patients who lack this capacity
- In common with other cluster randomised controlled trials of behaviour change interventions we are unable to blind nurses, participants or data collectors to trial arm allocation

Background

Patient experience of nursing care is correlated with safety, clinical effectiveness, care quality, treatment outcomes including mortality and overall service use.(1-7) Nursing care is a key determinant of patient experience(8, 9) and satisfaction.(10) Effective nursing care includes the establishment of compassionate nurse-patient relationships known as 'relational nursing care' in order to facilitate nurses' 'transactional care' behaviours (the physical work undertaken by nurses) to meet the fundamental care needs of patients such as nutrition, hydration, skin integrity, personal care, mobility, hygiene, breathing, elimination and mental wellbeing.(11, 12)

Measuring patient experience is analogous to collecting a patient-reported outcome measure (PROM)(13) in that both are "*a measurement based on a report that comes directly from the patient (i.e., study subject*)".(14) However, whereas a PROM captures "*the status of a patient's health condition without amendment or interpretation of the patient's response by a clinician or anyone else*",(14) a patient-reported *experience* measure (PREM) is a measure of a patient's perception of their personal experience of the healthcare they have received.(15) Two PREMs for evaluating patients' experiences of transactional and relational nursing have been developed and tested; for

For peer review only - http://bmjopen.bmj.com/site/about/guidelines.xhtml

transactional nursing, the Relational Aspects of Care Questionnaire – RACQ),(8, 16) and for relational nursing the Quality from the Patient's Perspective – QPP.(17, 18)

The combination of COVID-19 symptoms and high risk of transmission of the SARS-CoV-2 virus poses unique challenges for both transactional and relational nursing care. Previously, the 2003 SARS outbreak demonstrated that nurses' ability to provide compassionate fundamental care was compromised: "*The establishment/maintenance of therapeutic nurse-client relationship required additional time given the barriers of mask, gloves and gowns*".p4(19) "*Restrictions on visitors were difficult for staff because family members are usually involved in the social, psychological and, to some extent, physical care of patients*".p6(20) "*Interaction time decreased, and patients began to feel more abandoned*".p28(21)

During the development of our trial protocol described below, we found no international evidence-based guidelines for nursing hospitalised patients with SARS-CoV-2, the majority of whom are not invasively mechanically ventilated. This leads to the potential for variations in patient experience, treatment outcomes, care quality and costs, as reported by nurses who responded to our survey, also described below. Although individual nurses, teams and organisations reported rapidly adapting their procedures to nurse these patients, compared to usual nursing care, we as yet do not know how to optimise nursing care for the specific challenges of nursing COVID-19 patients, including mitigating the impact of wearing personal protective equipment, nor how effective these adaptations are in terms of these important outcomes.

Between July and October 2020, we developed a clinical nursing protocol. We undertook a rapid review(22) of published literature to identify the evidence for the effectiveness of and barriers to fundamental nursing care procedures in patients with SARS-CoV-2 virus or other conditions requiring isolation, in terms of overall patient experience, care quality, functional ability and treatment outcomes – PROSPERO registration number: CRD4202020091.(23) We also ran a UK-wide survey of nurses and non-registered care staff working with hospitalised patients with the SARS-CoV-2 virus who are not invasively ventilated to identify respondents' views on 'missed care',(24, 25) barriers to care, and innovation strategies respondents have adopted to meet the fundamental care needs of such patient. We obtained research governance approval from the Health Research Authority (IRAS project ID: 287288, protocol number 1920/31) and ethical approval from the University of Exeter research ethics committee (no: 20/07/256) for this survey. We then convened three consensus development panels(26-28) of nurses, and patients with experience of hospitalisation with the SARS-CoV-2 virus, to agree the content and design of the clinical nursing protocol. We will report the results of our systematic review and survey elsewhere.

For peer review only - http://bmjopen.bmj.com/site/about/guidelines.xhtml

In the COVID-NURSE trial, our objectives are:

a) to compare an evidence-based nursing protocol for patients with the SARS-CoV-2 virus against care as usual, in terms of patients' reported experience of transactional and relational nursing care, care quality, treatment outcomes and costs

b) to compare the effects of using the clinical protocol on nurses' moral distress

Methods: Participants, Interventions, and Outcomes

Design: we will undertake a cluster randomised controlled superiority trial(29, 30) with embedded iterative process(31) and economic evaluations(32) between 02/11/2020 and 18/10/2021). Our initial funded, approved and registered design was a rapid-cycle cluster randomised controlled trial(33) with three review 'waves'. Like adaptive trial designs, rapid cycle trials embed *a priori* opportunities into trial designs to enable review and minor adaptions to interventions between waves. We also planned to pair-match 18 trial sites according to two variables with a potential impact on outcomes: 1) ethnicity, where being of Black and Asian ethnicity increases the risk of COVID-19 infection compared to White individuals and where people of Asian ethnicity may be at higher risk of ITU admission and death (34) identified according to the latest census data for the local authority in which a hospital is located (Low (<68%) vs Medium (68% to 74%) vs High (>74%) non-BAME categories based around an overall mean of 71% non-BAME admissions; and 2) research intensity which is associated with improved hospital outcomes(35) identified according to two categories: research intensive vs not research intensive, where research intensive is defined as ≥0.1 unique recruiting studies in the 2019-2020 Q4 open data from NIHR divided by number of inpatient elective admissions in Jan-Mar 2020, and non-research intensive is <0.1.

Since submitting this protocol for peer review by this journal, experience enrolling wave one sites during October 2020-January 2021 taught us that our ideal situation is subject to considerable stress as sites adapt to a rapidly changing situation on the ground, facing significant clinical and research pressures. Although we successfully recruited three pairs (six sites) of sites matched as above for our planned first wave during October-December 2020, operational difficulties caused by a) the trial not being prioritised for NHS research infrastructure resources and b) the impact of the second wave of the SARS-COV-2 virus on nursing sickness levels, led to very significant delays to site opening. Our six wave one sites were unable to start data collection concurrently, delaying our planned wave one review. Further, we recruited several other sites for waves two and three for which we could not find a match on the variables listed above. These issues threatened to prevent us recruiting to time and target within the funded period (July 2020-April 2021). Therefore, in order for the trial to recruit sufficient sites and participants and for us to retain our ability to review and make minor

For peer review only - http://bmjopen.bmj.com/site/about/guidelines.xhtml

amendments to the intervention in response to process data on fidelity and acceptability, we amended the design to a simple cluster randomised trial, with an embedded iterative process evaluation. As a consequence, as part of the statistical analysis plan, we will also adjust for ethnicity of the patient sample at cluster level and, where patient outcomes are tested, at patient level rather than pre-randomisation. Finally, we revised our sample size calculations, see below and obtained additional funding to continue the trial for a further six months from the previous planned end date of 18th April 2021. We began data collection on 18th January 2021 and plan to continue until the end of August 2021.

Setting: we will recruit 14 UK district-general and teaching hospital NHS Trusts (clusters).

Participant eligibility criteria: patients who are not invasively ventilated, aged ≥18 years, currently hospitalised and being treated for infection with the SARS-CoV-2 virus, or recently discharged after such treatment and who have received nursing care for a minimum of 72 hours during their admission; registered nurses and nursing care workers working under the supervision of registered nurses caring for patients hospitalised and treated for infection with the SARS-CoV-2 virus. Participants must be able to give informed consent (see supplementary appendices). We will provide translation facilities for participants unable to understand and speak English. We will recruit participants specifically from wards allocated to the care of patients admitted for treatment of COVID-19 symptoms.

Interventions:

Experimental Intervention: care as usual plus the clinical protocol developed as described above. The protocol consists of four elements adapted from the methods used successfully in a previous cluster randomised controlled trial to significantly increase handwashing by nurses and care staff(36): 1) a guideline outlining strategies to overcome the barriers to care identified in our review, survey and consensus panels; 2) a staff education programme hosted on the Open University's 'FutureLearn' platform,(37) consisting of videos, written information and exercises to address barriers and nursing strategies; 3) reminders in the form of posters with key information on the same strategies to overcome barriers to care; 4) leadership from ward managers and senior nurses, to address the organisation of care, education for nursing teams and support for nurses caring for patients hospitalised with the SARS-CoV-2 virus, information on this delivered via the 'FutureLearn' platform.(37)

We will monitor clinical protocol fidelity and acceptability as part of our process evaluation and modify our guideline, educational and leadership strategies iteratively, making minor adjustments to

For peer review only - http://bmjopen.bmj.com/site/about/guidelines.xhtml

these elements according to findings from our process evaluation quantitative and qualitative data. We will embed any changes to the protocol in the subsequent experimental intervention sites.

Control: care as usual only. We will record staffing staff skill mix details alongside a description of clinical nursing procedures and organisation in place in the usual care clusters.

Outcomes: we will collect participant-level outcome data. Our co-primary outcomes will be patient-reported experience measures addressing transactional nursing care using the QPP(17, 18) and relational nursing care using the RACQ.(8, 16) Our secondary outcomes will include: measures of quality of care (pressure injuries, falls, medication errors) collected by the United Kingdom National Health Service Ward to Board dashboard(38); functional ability by the Barthel Index;(39, 40) treatment outcomes by the WHO Clinical Progression Scale;(41) depression by the PHQ-2,(42), anxiety by the GAD-2,(43), health utility by the EQ5D,(44) and nurse outcomes by the Measure of Moral Distress for Health Care Professionals.(45, 46)

Participant timeline: we will undertake the trial during 50 weeks of intervention and rolling data collection, with data collected from patient participants admitted for treatment of SARS-CoV-2 infection and staff caring for them.

Target sample size:

Using published formulae by Hayes & Moulton (16) we calculated our patient participant sample size based on an estimate of the minimum clinically important difference for the QPP of 0.2 and the typical within-unit standard deviation (0.6), supplied by the measure developers, and an ICC of 0.02. We have examined a number of potential scenarios taking into account different cluster sizes and different between-cluster standard deviation values informed by between-country differences on measures, estimated to provide 90% and 80% power. Based on conversations on feasibility with NHS clinical leaders, with a cluster size of 60 the trial would generate over 80% power with 6 clusters per arm and 90% power with 7 clusters in each arm. However, because power calculations in cluster trials with a small number of clusters can be especially sensitive to the approximation used for degrees of freedom, we used a range of alternative approaches (no small sample adjustment, Satterthwaite approximation, and Kenward-Rogers approximation; cluster-level linear regression) to explore the robustness of the sample size calculations. While these tended to estimate reduced power at each number of clusters compared to a formula-based power calculation, an estimate of 14 clusters (7 per arm) generated power of greater than 80% in every case. This number of clusters is fairly robust to number of patients per cluster. Even assuming under-recruitment leading to 50 patients per site, power is maintained at greater than 80%. Therefore, we will aim to recruit 840 patient participants. Although we have not powered the sample size on nurse participant outcomes,

For peer review only

in consultation with clinical colleagues in our investigator and advisory groups, we anticipate that around 50 registered nurses and healthcare support workers will be involved in the direct care of these patient participants leading to a recruitment target of 700 registered nurse and nursing care worker participants, including student nurses.

Recruitment: we will recruit cluster sites via the NHS chief nurse and clinical research networks (CRN). We will monitor available data from Public Health England on case and hospitalisation rates by NHS Trust in order to identify recruitment sites where there are sufficient potential participants. We will use these data to prepare sites for involvement. Clinical research nurses will recruit patient participants from admitted patients according to the eligibility criteria above.

Allocation: we will randomly allocate sites to the intervention and control groups by an unblinded statistician who has no role in site recruitment or data analysis to ensure allocation concealment; only the unblinded statistician will have access to the allocation list. Randomisation will use a static blocked list through the use of an externally administered, password-protected randomisation website independently developed and maintained by the UKCRC-registered University of Exeter Clinical Trials Unit.

Data collection: we will collect outcome data from patient participants after they have received nursing care for a minimum of 72 hours, either during their admission or a maximum of two weeks post discharge. All outcome measures will be applied to all participants equally and collected by clinical research nurses and other Good Clinical Practice(47) trained research staff, who will also obtain consent. It will not be possible to blind research nurses, clinicians or patient participants to allocation. Statistical analysts will be blind to group allocation when receiving data to analyse. We will collect outcome data either face to face or by video/audio technology according to the infection control procedures in place and participant preference. We will collect outcome data from nurses either face to face in their hospital, or over the phone, according to their preference. Site level *Ward to Board* data will be collected from routine hospital statistics.

Data management: participants will be identified by a unique study ID**.** Personal identifiable data including date of birth and NHS number, may be collected but will be stored separately to research data and will be destroyed as per applicable regulations when the project is concluded. Data will be managed by the UKCRC registered Exeter Clinical Trials Unit (ExeCTU) following GDPR and data protection guidelines and all relevant Clinical Trial Regulations. All data will be anonymised prior to publication. Data will be collected and stored electronically in accordance with the Data Protection Act 2018 and ICH GCP E6 R2. ExeCTU will use Redcap Cloud Electronic Data Capture System to collect all case report data. This system is validated to ISO27001 standards, backed up and maintained in

For peer review only - http://bmjopen.bmj.com/site/about/guidelines.xhtml

Europe. This system is fully compliant to GDPR regulations and managed by ExeCTU. Any additional study data will be stored and backed up on the secure ExeCTU servers, maintained by ExeCTU. Data will be cleaned and validated appropriately and a full Data Protection Impact Assessment (DPIA) will be undertaken along with the development of a comprehensive Data Management Plan (DMP) before the first participant is recruited.

Where data are disseminated (e.g. via report, presentation or publication), they will be anonymised. We will align all confidentiality and data handling with the Caldicott Principles.(48) Anonymised data will be stored indefinitely on a research data storage system provided by the University of Exeter called Open access Research Exeter (ORE) for archiving (http://www.exeter.ac.uk/research/openresearch/policies/ore/).

Statistical data analysis: we will analyse numerical data in Stata v.16.(49) Our primary analysis for patient and nurse outcomes will use a standard generalised linear mixed-effect model with appropriate small-sample adjustment to account for the small number of clusters, and adjusting for ethnicity of the patient sample at cluster level and, where patient outcomes are tested, at patient level as well. Sensitivity analyses for patient and nurse outcomes will use variance-weighted cluster-level summaries with adjustment for ethnicity based on 2011 census and hospital postcode and for research intensity. Analysis of hospital outcomes will use rate ratios. Secondly, and focusing on patient-level outcomes, we will estimate intervention effectiveness in an exploratory analysis. This analysis will use generalised linear mixed-effects models to model time trends. Analysis will include time from implementation, reflecting growing adaptation and expertise, as well as calendar time, reflecting the course of the SARS-CoV-2 epidemic. We will also undertake a planned sub-group analysis to compare the impact of patient-level ethnicity on our primary and secondary outcomes.

Missing data: data are collected from patients and nurses at one time point. As a result, missingness is likely to be at item-level within scales and at scale level. Where >50% of items in a scale are completed (including 'not applicable' or 'don't know' responses), a scale score will be generated by either taking the average of remaining items (all QPP subscales, RAC-Q-14, MMD-HP) or by rescaling sum scores to the full range (Barthel Index). This is an appropriate strategy when factor loadings are homogeneous and reliability for scales is good.(50) Observations from single-item or two-item scales (WHO Clinical Progression Scale, PHQ-2, GAD-2) will be dropped from the analysis.

Process evaluation: we will evaluate the impact of nurses' and nursing care workers' fidelity to the clinical protocol, intervention mechanisms and context of care delivery on outcomes using: a bespoke instrument for capturing fidelity to reflect the protocol components; the Culture of Care Barometer Questionnaire;(51) the NoMAD instrument for measuring implementation readiness and

For peer review only - http://bmjopen.bmj.com/site/about/guidelines.xhtml

acceptability;(52, 53) contextual data on patient acuity, staffing ratios, nurses/care staff education levels, seniority and year of qualification, and other contextual variables. We will conduct interim analyses of process evaluation data on fidelity to the protocol, and protocol utilisation to evaluate intervention component fidelity and implementation. We will subsequently amend the clinical protocol based on this data before embedding the new protocol in intervention sites.

We will conduct semi-structured qualitative interviews with a purposive sample of nurses and care staff, using a topic guide derived from the content of the clinical protocol and our analysis of quantitative data, to explore their views on the mechanisms, impact and acceptability of the clinical protocol. This method will enable us to investigate the meaning of participants' responses, both exploring views on our predefined topics of interest and eliciting more detail on any emerging themes.(54) Interviews will be conducted using telephone or online audio-conference methods, unless face to face interviews are risk assessed as being safe and are preferred by participants. With participants' consent (see supplementary materials), we will audio-record interviews and transcribe interviews verbatim. We will use NVivo10(55) to organise the data and analyse data using Framework analysis to allow for the combination of inductive and deductive approaches in our development of themes.(56) We will use joint displays(57) to integrate the qualitative data with the quantitative outcome data in a mixed methods analysis(58) to explore and explain variation in the outcome data. We will use this data to refine an intervention programme theory(59, 60) incorporating context, mechanisms and fidelity.(31)

Economic evaluation: we will conduct within trial cost effectiveness analysis by adopting the NHS perspective with the main outcome measure the Quality-adjusted life year (QALY) derived from the EQ-5D-5L.(32, 44) We will apply the NICE threshold of £20,000 per QALY. Cost data will include staff training, length of stay, including ICU costs and COVID-specific interventions from baseline to discharge using a bespoke hospital care use inventory. We will apply unit costs derived from national sources including Personal Social Services Research Unit (61) and NHS Reference Costs.(62) Non-parametric bootstrapping will be used to quantify uncertainty. We will present outputs as ICERs, cost effectiveness acceptability curves and expected net benefit.

Monitoring: day to day management of the trial and study will be by the chief-investigator, other co-investigators and a research manager. We will set up an independent combined Trial Steering and Data Monitoring Committee (TSC/DMC)(47) and a Patient and Public Involvement group.(63) We will undertake a risk assessment of the trial and produce a monitoring plan that will be commensurate with the risk identified to particularly ensure that we have input from people of colour, including from Black, Asian and other minority ethnic groups, who are at greater risk from infection with the SARS-CoV-2 virus.

For peer review only - http://bmjopen.bmj.com/site/about/guidelines.xhtml

Safety reports of serious adverse events (SAE)

Given this trial is not a clinical trial of an investigational medicinal product (CTIMP) the recording and reporting of non-serious adverse events (AEs) is not required(64) since, although likely to be common in the target population, they are unlikely to be related to either the intervention or trial participation. Likewise, by nature of the patient participants' clinical condition, we will expect some serious adverse events (SAEs), specifically death or prolongation of hospital stay, (64) but that these will be both expected and unlikely to be related to intervention or trial procedures. As all non-CTIMP research is required to adhere to the principles of Good Clinical Practice(47, 65) it was nonetheless agreed with the trial sponsor, research ethics committee and independent TSC/DMC to record and review each SAE by the CI on a case-by-case basis to determine if it is related to the trial intervention or procedures. This process is overseen by the TSC/DMC.

Patient and Public Involvement: The proposed study has been informed by ongoing collaborations between patients, clinicians and researchers, including in a previous NIHR-funded project (ESSENCE) on fundamental nursing care. A version of the lay summary was reviewed by six members of the 'Peninsula Patient Involvement Group', giving feedback on the broad idea of the research as well as specific points on the clarity of the summary. A patient co-applicant has been involved with the development of the proposal. We will ensure there is a patient voice in all discussions and decisions, and will ensure the integration of broader PPI activity in these decisions. We have worked with a wider group of patients with experience of hospitalisation for COVID-19 to inform the co-production of the nursing protocol, development of all patient facing materials, finalising the nursing protocol for rapid-cycle testing, adaptions to the nursing protocol after each cycle of testing, dissemination of findings, including the production of an online training module for nurses on the FutureLearn platform. The involvement of patients will be informed by what is possible within the ongoing pandemic, and to suit the situation of interested patients. Involvement will be flexible and responsive to their needs. We will adopt high standards of equality, diversity and inclusivity(66) throughout.

Ethical issues: we will conduct this trial in such a way as to protect the human rights and dignity of the participants, as reflected in the Helsinki Declaration.(67) We have obtained governance and ethical approval from the Health Research Authority National Research Ethics Service (IRAS project ID 288479; REC reference: 20/NE/0253). Participants will not receive any financial inducement to participate. We will invite eligible patients and nurses to participate in data collection and will only collect data from participants who individually consent. Participants may withdraw from the study at any time without prejudicing their treatment, care or employment. We will conform to Good Clinical Practice Guidelines, data protection and freedom of information acts. We registered the trial with

For peer review only - http://bmjopen.bmj.com/site/about/guidelines.xhtml

the ISRCTN registry prospectively on 9th September 2020, before our intended data collection start date and have registered protocol amendments when required.

Outputs and dissemination: we will disseminate our results through publication in peer reviewed scientific journals, our study website and other press and online media. Manuscript authors will be those considered to have made a substantive intellectual contribution to the study. The investigators and relevant authorities will have access to the trial dataset. Furthermore, we will store anonymised research data and outputs in the University of Exeter's Open Research Exeter repository (https://ore.exeter.ac.uk/repository/) in order to facilitate open access to, and the impact of, our research.

The main output from this study will be evidence on whether our COVID-specific clinical nursing care protocol delivers additional benefits in overall patient experience, care quality, functional ability and treatment outcomes, with cost-effectiveness estimates, for hospitalised patients with the SARS-CoV-2 virus not invasively ventilated, compared to care as usual in hospital wards. We will have a programme theory allowing us to potentially generalise any such benefits to environments like care homes, patients with other conditions requiring isolation and to global health systems. If effective, our guidelines, education materials and strategies will be made accessible via Health Education England, the Open University's FutureLearn(37) web platform, and through experienced NHS sites acting as training and dissemination hubs for other health Trusts, environments such as care homes, and global health systems.

Discussion

Our research aligns with the WHO COVID-19 R&D Roadmap priorities and research gaps,(68) viz: 'to determine optimal clinical practice strategies to improve the processes of care'. It includes 'rapid approaches to capture healthcare worker views (surveys, interviews)' and a rapid ethnograph[y] in healthcare'.(68) It will, therefore, provide guidance to nurses attempting to overcome significant barriers to providing fundamental transactional and relational care to patients; specifically those with the SARS-CoV-2 virus not invasively ventilated but with a generalisable programme theory suitable for adaptation to other care environments, other pandemics and other nations globally.

Authors' contributions: DR wrote the protocol with input from co-investigators HVRS, EC, JC, SC, CH, PL, HI-S, GJM-T, NR, AMRa, AMRu, MS, SJS, J, T-C, ST and SW. All other authors (RA, AB, SC, FD, LQ, HT, FW, RW, JB, HAH, KM, LM, NM and LR) reviewed the manuscript and made editorial suggestions. FD and NR are independent patient and public Involvement members of the team, GJM-T is the senior trial statistician and CH the trial economist. The trial team include nurses (DR, JC, SC, HI-S, GJM-T, AMRa, AMRu, MS, ST, LM, NM, LR), a physiotherapist (SJS), an occupational therapist (PL), a

For peer review only - http://bmjopen.bmj.com/site/about/guidelines.xhtml

dietician (SW), medical statisticians (GJM-T, SC, FCW), an information specialist (AB), systematic reviewers (JT-C, RA, RW), a health economist (CH), health scientists and clinical trialists (DAR, HVRS, LQ, HT, JB, HAH), patient and public involvement specialists (EC, FD, NR) and an administrator (MK).

Funding statement: 'This work is supported by National Institute for Health Research and UK Research and Innovation, administered by the MRC: grant number MR/V02776X/1. The Trial Sponsor is the University of Exeter, ref: 1920/ Research Ethics and Governance Office, Lafrowda House, St Germans Road, Exeter, Devon, EX4 6TL. Neither the funder nor sponsor will have any role or ultimate authority in study design; collection, management, analysis, and interpretation of data; writing of the report; and the decision to submit the report for publication.

Competing interests: the authors confirm that they have no conflict of interests to declare

For peer review only

1. Darzi A. Quality and the NHS next stage review. Lancet. 2008;371(9624):1563-4.
2. Vincent C, Amalberti R. Safer Healthcare: Strategies for the Real World. Cham (CH)2016.
3. Black N, Varaganum M, Hutchings A. Relationship between patient reported experience (PREMs) and patient reported outcomes (PROMs) in elective surgery. Bmj Qual Saf. 2014;23(7):534-42.
4. Doyle C, Lennox L, Bell D. A systematic review of evidence on the links between patient experience and clinical safety and effectiveness. BMJ Open. 2013;3(1).
5. Department of Health. Report of the Mid Staffordshire NHS Foundation Trust Public Enquiry. London: Department of Health; 2013.
6. @BorisJohnson. It is hard to find the words to express my debt to the NHS for saving my life. The efforts of millions of people across this country to stay home are worth it. Together we will overcome this challenge, as we have overcome so many challenges in the past. #StayHomeSaveLives [twitter post]12/04/2020 10/05/2020.
7. Aiken LH, Sloane DM, Bruyneel L, Van den Heede K, Griffiths P, Busse R, et al. Nurse staffing and education and hospital mortality in nine European countries: a retrospective observational study. Lancet. 2014;383(9931):1824-30.
8. Graham C, Kasbauer S, Cooper R, King J, Sizmur S, Jenkinson C, et al. An evaluation of a near real-time survey for improving patients' experiences of the relational aspects of care: a mixed-methods evaluation. Health Services and Delivery Research. Southampton (UK)2018.
9. Murrells T, Robert G, Adams M, Morrow E, Maben J. Measuring relational aspects of hospital care in England with the 'Patient Evaluation of Emotional Care during Hospitalisation' (PEECH) survey questionnaire. BMJ Open. 2013;3(1).
10. Aiken LH, Sloane D, Griffiths P, Rafferty AM, Bruyneel L, McHugh M, et al. Nursing skill mix in European hospitals: cross-sectional study of the association with mortality, patient ratings, and quality of care. Bmj Qual Saf. 2017;26(7):559-68.
11. Feo R, Conroy T, Jangland E, Muntlin Athlin A, Brovall M, Parr J, et al. Towards a standardised definition for fundamental care: A modified Delphi study. J Clin Nurs. 2018;27(11-12):2285-99.
12. Kitson A, Conroy T, Wengstrom Y, Profetto-McGrath J, Robertson-Malt S. Defining the fundamentals of care. Int J Nurs Pract. 2010;16(4):423-34.
13. Calvert MJ, Freemantle N. Use of health-related quality of life in prescribing research. Part 1: why evaluate health-related quality of life? J Clin Pharm Ther. 2003;28(6):513-21.
14. FDA-NIH Biomarker Working Group. BEST (Biomarkers, EndpointS, and other Tools) Resource Silver Spring and Bethesda (MD): Food and Drug Administration (US) and National Institutes of Health (US); 2016 [updated 2020. Available from: https://www.ncbi.nlm.nih.gov/books/NBK326791/.
15. Coulter A. Measuring what matters to patients. BMJ. 2017;356:j816.
16. Kelly L, Sizmur S, Kasbauer S, King J, Cooper R, Jenkinson C, et al. The Relational Aspects of Care Questionnaire: item reduction and scoring using inpatient and accident and emergency data in England. Patient Relat Outcome Meas. 2018;9:173-81.

For peer review only - http://bmjopen.bmj.com/site/about/guidelines.xhtml

17. Wilde Larsson B, Larsson G. Development of a short form of the Quality from the Patient's Perspective (QPP) questionnaire. J Clin Nurs. 2002;11(5):681-7.
18. Larsson B, Larsson G, Chantereau M, von Holstein K. International comparisons of patients' views on quality of care. Int J Health Care Qual Assur Inc Leadersh Health Serv. 2005;18(1):62-73.
19. Canadian Nurses Association. CNA Brief to the National Advisory Committee on SARS and Public Health: Lessons learned and recommendations. Ottawa, Canada: Canadian Nurses Association; 2003.
20. Baumann A, Blythe, J., Underwood, J., Dzuiba, J.,. Capacity, casualization, and continuity: the impact of SARS Toronto, Canada: University of Toronto, Faculty of Nursing and McMaster University, School of Nursing; 2003.
21. Registered Nurses Association of Ontario. SARS Unmasked: Celebrating Resilience, Exposing Vulnerability. A Report on the Nursing Experience with SARS in Ontario. Toronto: Registered Nurses Association of Ontario; 2003.
22. Dobbins M. Rapid review guidebook. Hamilton, ON: National Collaborating Centre for Methods and Tools; 2017.
23. Thompson-Coon J, Whear R, Abbott R, Bethel A, Cockcroft E, Cooper J, et al. Effectiveness of and barriers to fundamental nursing care procedures in patients with SARS-COV-2 virus: a rapid systematic review. 28/07/2020 ed: PROSPERO 2020 CRD42020200914; 2020.
24. Ball JE, Bruyneel L, Aiken LH, Sermeus W, Sloane DM, Rafferty AM, et al. Post-operative mortality, missed care and nurse staffing in nine countries: A cross-sectional study. Int J Nurs Stud. 2018;78:10-5.
25. Ball JE, Murrells T, Rafferty AM, Morrow E, Griffiths P. 'Care left undone' during nursing shifts: associations with workload and perceived quality of care. Bmj Qual Saf. 2014;23(2):116-25.
26. Halcomb E, Davidson P, Hardaker L. Using the consensus development conference method in healthcare research. Nurse Res. 2008;16(1):56-71.
27. Nair R, Aggarwal R, Khanna D. Methods of formal consensus in classification/diagnostic criteria and guideline development. Semin Arthritis Rheum. 2011;41(2):95-105.
28. Waggoner J, Carline JD, Durning SJ. Is There a Consensus on Consensus Methodology? Descriptions and Recommendations for Future Consensus Research. Acad Med. 2016;91(5):663-8.
29. Campbell MK, Elbourne DR, Altman DG, Consort Group. CONSORT statement: extension to cluster randomised trials. British Medical Journal. 2004;328(7441):702-8.
30. Hayes RJ, Moulton LH. Cluster Randomised Trials 2nd Edition. Florida: Chapman and Hall/CRC; 2017.
31. Moore GF, Audrey S, Barker M, Bond L, Bonell C, Hardeman W, et al. Process evaluation of complex interventions: Medical Research Council guidance. BMJ. 2015;350:h1258.
32. National Institute for Health and Care Excellence. Guide to the methods of technology appraisal. London: National Institute for Health and Care Excellence; 2013.
33. Johnson K, Gustafson D, Ewigman B, Provost L, Roper R. Using Rapid-Cycle Research to Reach Goals: Awareness, Assessment, Adaptation, Acceleration. AHRQ Publication No. 15-0036. Rockville (MD): Agency for Healthcare Research and Quality; 2015.

For peer review only

34. Sze S, Pan D, Nevill CR, Gray LJ, Martin CA, Nazareth J, et al. Ethnicity and clinical outcomes in COVID-19: A systematic review and meta-analysis. EClinicalMedicine. 2020;29:100630.
35. Downing A, Morris EJ, Corrigan N, Sebag-Montefiore D, Finan PJ, Thomas JD, et al. High hospital research participation and improved colorectal cancer survival outcomes: a population-based study. Gut. 2017;66(1):89-96.
36. Huis A, Schoonhoven L, Grol R, Donders R, Hulscher M, van Achterberg T. Impact of a team and leaders-directed strategy to improve nurses' adherence to hand hygiene guidelines: a cluster randomised trial. Int J Nurs Stud. 2013;50(4):464-74.
37. FutureLearn. London: The Open University and The SEEK Group; [Available from: https://www.futurelearn.com.
38. NHS Improvement. Guide to developing and implementing ward and unit accreditation programmes, IG 09/19. London: NHS Improvement; 2019.
39. Mahoney FI, Barthel DW. Functional Evaluation: The Barthel Index. Md State Med J. 1965;14:61-5.
40. Collin C, Wade DT, Davies S, Horne V. The Barthel ADL Index: a reliability study. Int Disabil Stud. 1988;10(2):61-3.
41. Marshall J, On Behalf Of The Who Working Group On The Clinical Characteristics Of Covid-19 Infection,. A Core Outcome Measure Set for Studies of SARS-CoV2/COVID-19 infection Toronto, Canada: University of Toronto; 2020 [Available from: http://www.comet-initiative.org/Studies/Details/1528.
42. Kroenke K, Spitzer RL, Williams JB. The Patient Health Questionnaire-2: validity of a two-item depression screener. Med Care. 2003;41(11):1284-92.
43. Kroenke K, Spitzer RL, Williams JB, Monahan PO, Lowe B. Anxiety disorders in primary care: prevalence, impairment, comorbidity, and detection. Ann Intern Med. 2007;146(5):317-25.
44. EuroQol Research Foundation. EQ-5D-5L User Guide. Available from: https://euroqol.org/publications/user-guides2019.
45. Epstein EG, Whitehead PB, Prompahakul C, Thacker LR, Hamric AB. Enhancing Understanding of Moral Distress: The Measure of Moral Distress for Health Care Professionals. AJOB Empir Bioeth. 2019;10(2):113-24.
46. Corley MC, Elswick RK, Gorman M, Clor T. Development and evaluation of a moral distress scale. J Adv Nurs. 2001;33(2):250-6.
47. Medical Research Council. MRC Guidelines for Good Clinical Practice in Clinical Trials. London: Medical Research Council; 1998.
48. The Caldicott Report. IHRIM. 1999;40(2):17-9.
49. Stata Corp. Stata Statistical Software: Release 16. College Station, Texas, USA: Stata Corp LLC; 2019.
50. Graham JW. Missing Data: Analysis and Design, Statistics for Social and Behavioural Sciences. New York: Springer; 2012.
51. Rafferty AM, Philippou J, Fitzpatrick JM, Pike G, Ball J. Development and testing of the 'Culture of Care Barometer' (CoCB) in healthcare organisations: a mixed methods study. BMJ Open. 2017;7(8):e016677.
52. Finch TL, Girling M, May CR, Mair FS, Murray E, Treweek S, et al. Improving the normalization of complex interventions: part 2 - validation of the NoMAD instrument for assessing implementation work based on normalization process theory (NPT). BMC Med Res Methodol. 2018;18(1):135.

For peer review only

53. Rapley T, Girling M, Mair FS, Murray E, Treweek S, McColl E, et al. Improving the normalization of complex interventions: part 1 - development of the NoMAD instrument for assessing implementation work based on normalization process theory (NPT). BMC Med Res Methodol. 2018;18(1):133.
54. Gale NK, Heath G, Cameron E, Rashid S, Redwood S. Using the framework method for the analysis of qualitative data in multi-disciplinary health research. BMC Med Res Methodol. 2013;13:117.
55. QSR International. NVivo 10 for windows 2017 [Available from: www.qsrinternational.com/products_nvivo.aspx. .
56. Ritchie J, Spencer L, W OC. Qualitative research practice: A guide for social science students and researchers. London: Sage; 2003.
57. Creswell JW, Plano-Clark VL. Designing and conducting mixed methods research. Thousand Oaks: Sage; 2007.
58. Richards DA, Bazeley P, Borglin G, Craig P, Emsley R, Frost J, et al. Integrating quantitative and qualitative data and findings when undertaking randomised controlled trials. BMJ Open. 2019;9(11):e032081.
59. Rogers P. Using programme theory to evaluate complicated and complex aspects of interventions. Evaluation. 2008;14(1):29-48.
60. Funnell S.C., Rogers P.J. Purposeful Program Theory. Effective Use of Theories of Change and Logic Models. San Francisco: Jossey-Bass/Wiley; 2011.
61. Curtis L, Burns A. Unit Costs of Health and Social Care 2019. University of Kent, Canterbury: Personal Social Services Research Unit; 2019.
62. Department of Health. NHS reference costs 2018-2019. London: Department of Health; 2019.
63. INVOLVE. Briefing notes for researchers: involving the public in NHS, public health and social care research. Eastleigh: INVOLVE; 2012.
64. Health Research Authority. Safety reporting 2020 [Available from: https://www.hra.nhs.uk/approvals-amendments/managing-your-approval/safety-reporting/.
65. Health Research Authority. UK policy framework for health and social care research v3.3. London: National Health Service; 2017.
66. National Institute for Health Research. Strengthening our commitment to equality, diversity, inclusion and patient and public involvement and engagement (PPIE) 2020 [updated 01/05/2020. Available from: https://www.nihr.ac.uk/documents/strengthening-our-commitment-to-equality-diversity-inclusion-and-patient-and-public-involvement-and-engagement-ppie/24697.
67. World Medical Association. World Medical Association Declaration of Helsinki Ethical Principles for Medical Research Involving Human Subjects. Journal of the American Medical Association. 2013;310(20):2191-4.
68. World Health Organisation. 2019 novel Coronavirus Global research and innovation forum: towards a research roadmap. Geneva: World Health Organisation; 2020.

For peer review only - http://bmjopen.bmj.com/site/about/guidelines.xhtml

UNIVERSITY OF EXETER

COLLEGE OF MEDICINE AND HEALTH

This protocol has regard for the HRA guidance and order of content

FULL/LONG TITLE OF THE STUDY

COVID-NURSE: development and evaluation of the effects of a COVID-specific fundamental nursing care protocol compared to care as usual on experience of care for non-invasively ventilated patients in hospital with the SARS-CoV-2 virus: protocol for a randomised controlled trial

SHORT STUDY TITLE / ACRONYM

COVID-NURSE: RCT for nursing protocol intervention

PROTOCOL VERSION NUMBER AND DATE

V5.0 05/02/2021

RESEARCH REFERENCE NUMBERS

**IRAS Number:**	288479
**SPONSORS Number:**	1920/31
**FUNDERS Number:**	COVO243

For peer review only

For peer review only - http://bmjopen.bmj.com/site/about/guidelines.xhtml

SIGNATURE PAGE

The undersigned confirm that the following protocol has been agreed and accepted and that the Chief Investigator agrees to conduct the study in compliance with the approved protocol and will adhere to the principles outlined in the Declaration of Helsinki, the Sponsor's SOPs, and other regulatory requirement.

I agree to ensure that the confidential information contained in this document will not be used for any other purpose other than the evaluation or conduct of the investigation without the prior written consent of the Sponsor

I also confirm that I will make the findings of the study publically available through publication or other dissemination tools without any unnecessary delay and that an honest accurate and transparent account of the study will be given; and that any discrepancies from the study as planned in this protocol will be explained.

For peer review only

For and on behalf of the Study Sponsor:

Signature: Date:

05/02/2021

Name (please print): Ms Pam Baxter

Position: Senior Research Governance Officer

Chief Investigator:

Signature: Date: 05/02/2021

Name: (please print):

David Richards

For peer review only - http://bmjopen.bmj.com/site/about/guidelines.xhtml

LIST of CONTENTS

GENERAL INFORMATION	Page No.
HRA PROTOCOL COMPLIANCE DECLARATION	i
TITLE PAGE	i
RESEARCH REFERENCE NUMBERS	i
SIGNATURE PAGE	ii
LIST OF CONTENTS	iii
KEY STUDY CONTACTS	iv
STUDY SUMMARY	ix
FUNDING	x
ROLE OF SPONSOR AND FUNDER	xi
ROLES & RESPONSIBILITIES OF STUDY STEERING GROUPS AND INDIVIDUALS	xii
STUDY FLOW CHART	xvi
SECTION	
1. BACKGROUND	1
2. RATIONALE	1
3. THEORETICAL FRAMEWORK	2
4. RESEARCH QUESTION/AIM(S)	3
5. STUDY DESIGN/METHODS	3
6. STUDY SETTING	7
7. SAMPLE AND RECRUITMENT	8
8. ETHICAL AND REGULATORY COMPLIANCE	11
9. DISSEMINATION POLICY	17
10. REFERENCES	18
11. APPENDICES	20

For peer review only

For peer review only - http://bmjopen.bmj.com/site/about/guidelines.xhtml

KEY STUDY CONTACTS

Chief Investigator	David A Richards, PhD, RN<br>Professor of Mental Health Services Research and NIHR Senior Investigator Emeritus<br>Head of Nursing<br>Room 3.01 E South Cloisters<br>University of Exeter<br>St Luke's Campus<br>Heavitree Road<br>Exeter EX1 2LU<br>United Kingdom<br>Office: +44 (0) 1392 724615<br>Mobile: +44 7930393456
Study Co-ordinator	Dr Jessica Bollen<br>Trial Manager and Postdoctoral Research Fellow<br>Exeter Clinical Trials Unit<br>University of Exeter Medical School<br>College House | St Luke's Campus | Exeter | EX1 2LU<br>Tel 01392 726049<br>j.bollen@exeter/ac.uk
Sponsor	University of Exeter<br>Sponsor's representative<br>Ms Pam Baxter<br>Senior Research Governance Officer<br>University of Exeter<br>Research Ethics and Governance Office<br>Lafrowda House<br>St Germans Road<br>Exeter<br>Devon<br>EX4 6TL<br>Email: p.r.baxter2@exeter.ac.uk<br>Tel: 01392 723588<br>Mobile: 07485042117

For peer review only - http://bmjopen.bmj.com/site/about/guidelines.xhtml

Funder(s)	Medical Research Council/UKRI<br>2nd Floor DPB<br>Polaris House<br>North Star Avenue, Swindon<br>SN2 1FL<br>simon.fisher@mrc.ukri.org
Key Protocol Contributors	Professor G.J. Melendez-Torres<br>University of Exeter<br>Professor of Clinical and Social Epidemiology Director<br>South Cloisters, University of Exeter, St Luke's Campus, Heavitree Road, Exeter EX1 2LU<br>G.J.Melendez-Torres@exeter.ac.uk<br><br>Professor Siobhan Creanor<br>University of Exeter<br>Director of Exeter CTU, Professor of Medical Statistics & Clinical Trials<br>College House, University of Exeter, St Luke's Campus, Heavitree Road, Exeter EX1 2LU<br>E.S.Creanor@exeter.ac.uk<br><br>Dr Lynne Quinn<br>Director Of Operations, CTU<br>College House, University of Exeter, St Luke's Campus, Heavitree Road, Exeter EX1 2LU<br>l.quinn@exeter.ac.uk<br><br>Dr Emma Cockcroft<br>University of Exeter<br>Research Fellow in Patient and Public involvement<br>South Cloisters, University of Exeter, St Luke's Campus, Heavitree Road, Exeter, UK EX1 2LU<br>E.J.Cockcroft@exeter.ac.uk<br>01392 722764

For peer review only

For peer review only - http://bmjopen.bmj.com/site/about/guidelines.xhtml

	Dr Joanne Cooper<br>University of Nottingham<br>Assistant Director of Nursing (Research, Innovation & Professional Regulation)<br>joanne.cooper@nottingham.ac.uk<br>3rd Floor, Trust Headquarters Nottingham City Hospital Hucknall Road Nottingham NG5 1PB<br>0115 96 91169 x76395<br><br>Dr Susanne Cruickshank<br>The Royal Marsden NHS Foundation Trust<br>Strategic Lead for Applied Health Research<br>susanne.cruickshank@rmh.nhs.uk<br>The Royal Marsden Downs Road, Sutton Surrey SM2 5PT<br>020 7352 8171<br><br>Professor Claire Hulme<br>University of Exeter<br>Director of Institute of Health Research and Professor of Health Economics,<br>C.T.Hulme@exeter.ac.uk<br>South Cloisters, University of Exeter, St Luke's Campus, Heavitree Road, Exeter, EX1 2LU<br>01392 72 2902<br><br>Dr Heather Iles-Smith<br>Leeds Teaching Hospitals NHS Trust<br>Head of Nursing Research and Innovation<br>heather.iles-smith@nhs.net<br>Research & Innovation Department, Research & Innovation Centre,<br>St James's University Hospital, Beckett Street, LS9 7TF<br>0113 206 0469

For peer review only

For peer review only - http://bmjopen.bmj.com/site/about/guidelines.xhtml

	Dr Philippa Logan<br>University of Nottingham<br>Professor of Rehabilitation Research<br>pip.logan@nottingham.ac.uk<br>Room B108a Medical School Queen's Medical Centre Nottingham NG7 2UH<br>0115 823 0235<br><br>Professor Anne Marie Rafferty<br>King's College London<br>Professor of Nursing Policy<br>anne_marie.rafferty@kcl.ac.uk<br>NIHR Health & Social Care Workforce Research Unit King's College London Strand London WC2R 2LS<br>020 7848 3012<br><br>Ms Faye Doris<br>Patient and Public Involvement Representative<br>fedoris1933@gmail.com<br>South Cloisters, University of Exeter,<br>St Luke's Campus,<br>Heavitree Road,<br>Exeter, UK<br>EX1 2LU<br>01392 722764<br><br>Dr Anne-Marie Russell<br>University of Exeter<br>Senior Lecturer, Academy of Nursing<br>a.russell@imperial.ac.uk<br>South Cloisters, University of Exeter, St Luke's Campus, Heavitree Road, Exeter, UK EX1 2LU

For peer review only

For peer review only - http://bmjopen.bmj.com/site/about/guidelines.xhtml

	01392 722764<br><br>Professor Maggie Shepherd<br>University of Exeter, RD&E Hospital<br>Honorary Clinical Professor; Lead Nurse for Research<br>M.H.Shepherd@exeter.ac.uk<br>Medical School Building, St Luke's Campus, Magdalen Road, Exeter EX1 2LU<br>01392 408261<br><br>Professor Sally Singh<br>University of Leicester<br>Head of Pulmonary and Cardiac Rehabilitation Departments, Professor of Pulmonary and Cardiac Rehabilitation<br>leslie.shortt@uhl-tr.nhs.uk<br>Head of Cardiac/Pulmonary Rehabilitation Glenfield Hospital Groby Road Leicester LE3 9QP<br>0116 2583181<br><br>Dr Holly Sugg<br>University of Exeter<br>Lecturer, Academy of Nursing<br>H.V.R.Sugg@exeter.ac.uk<br>South Cloisters, University of Exeter, St Luke's Campus, Heavitree Road, Exeter, UK EX1 2LU<br>01392727412<br><br>Professor Jo Thompson Coon<br>University of Exeter<br>Professor of Evidence Synthesis and Health Policy<br>J.Thompson-Coon@exeter.ac.uk<br>South Cloisters, University of Exeter, St Luke's Campus, Heavitree Road, Exeter, , UK EX1 2LU<br>01392 726074

For peer review only

For peer review only - http://bmjopen.bmj.com/site/about/guidelines.xhtml

	Ms Susannah Tooze<br>University of Exeter<br>Senior Lecturer, Academy of Nursing<br>S.J.Tooze@exeter.ac.uk<br>South Cloisters, University of Exeter, St Luke's Campus, Heavitree Road, Exeter, UK EX1 2LU<br>01392 72 6970<br><br>Mr Harry Tripp<br>University of Exeter<br>IT Project Manager/ Senior Data Manager<br>H.E.Tripp@exeter.ac.uk<br>College House, University of Exeter, St Luke's Campus, Heavitree Road, Exeter UKEX1 2LU<br>01392722924<br><br>Professor Stephen Wootton<br>University of Southampton<br>Associate Professor in Human Nutrition<br>saw@soton.ac.uk<br>SGH/CF/MP113 University of Southampton University Road Southampton SO17 1BJ<br>023) 8120 4199

For peer review only

STUDY SUMMARY

Study Title	COVID-NURSE: development and evaluation of the effects of a COVID-specific fundamental nursing care protocol compared to care as usual on experience of care for non-invasively ventilated patients in hospital with the SARS-CoV-2 virus: protocol for a randomised controlled trial
Internal ref. no. (or short title)	COVID-NURSE: RCT for nursing COVID patients
Study Design	Cluster randomised controlled superiority trial with embedded process and economic evaluations.

For peer review only - http://bmjopen.bmj.com/site/about/guidelines.xhtml

Study Participants	Patients who are not invasively ventilated, aged ≥18 years, currently hospitalised and treated for infection with the SARS-CoV-2 virus and who have received nursing care for a minimum of 72 hours during their current admission; registered nurses and nursing care workers caring for patients hospitalised and treated for infection with the SARS-CoV-2 virus.
Planned Size of Sample (if applicable)	840 patient participants; 700 nursing participants
Follow up duration (if applicable)	N/A
Planned Study Period	02/11/2020 – 31/03/2021
Research Question/Aim(s)	What is the effect of an evidence-based nursing protocol for patients with the SARS-CoV-2 virus, compared to care as usual, in terms of patients' reported experience of transactional and relational nursing care, care quality, treatment outcomes and costs.

FUNDING AND SUPPORT IN KIND

**FUNDER(S)**<br>(Names and contact details of ALL organisations providing funding and/or support in kind for this study)	**FINANCIAL AND NON FINANCIALSUPPORT GIVEN**
Medical Research Council/UKRI<br>2nd Floor DPB<br>Polaris House<br>North Star Avenue, Swindon<br>SN2 1FL<br>simon.fisher@mrc.ukri.org	Funding to the value of £430,470

Role of Sponsor

The study sponsor will ensure that the research team has access to resources and support to deliver the research as proposed and that responsibilities for management, monitoring and reporting of the research are in place prior to the study commencing. The sponsor will ensure that there is agreement on recording, reporting and reviewing significant developments as the research proceeds and approve any modifications to design, obtaining requisite regulatory authority approval.

For peer review only - http://bmjopen.bmj.com/site/about/guidelines.xhtml

For peer review only

The sponsor will assume responsibility for operating the management and monitoring systems of the research.

Prior to the study commencing the sponsor will be satisfied that:

- The research will respect the dignity, rights, safety and well-being of participants and the relationship with healthcare professionals.
- Where appropriate the research has been reviewed and approved by an NHS Research Ethics Committee and/or the Health Research Authority Approval Programme.
- The Chief Investigator, and other key researchers have the requisite expertise and have access needed to conduct the research successfully.
- The arrangements and resources proposed for the research will allow the collection of high quality, accurate data and the systems and resources will allow appropriate data analysis and data protection.
- Organisations and individuals involved in the research agree the division of responsibilities between them.
- Arrangements are in place for the sponsor and other stakeholder organisations to be alerted to significant developments during the study, whether in relation to the safety of individuals or scientific direction.
- There are arrangements for the conclusion of the study including appropriate plans for the dissemination of findings.

The sponsor plays no role in the design of this study, and will have no role in data analysis or interpretation, or writing up of findings of the study.

For peer review only

Role of Funder

The research funder has the responsibility to ensure that there is a proper use of the funds they control. The study is funded as part of the MRC and UKRI COVID Rapid Response Call. The Funder has reviewed the study plan of the research and established that the research is worthwhile, of high scientific quality and represents good value for money. The research funder has assessed the experience and expertise of the Chief Investigator, other key researchers on the programme and has deemed that there is appropriate infrastructure for the research to be carried out.

For peer review only - http://bmjopen.bmj.com/site/about/guidelines.xhtml

The funding review process provided feedback on the design of the study plan. The funder plays no further role in the design of this individual study and will have no role in data analysis or interpretation, or writing up of findings of the study. The funder will be sent all outputs prior to dissemination, but has no role in the decision to submit for publication.

Trial Steering Committee (TSC) and Data Management Committee (DMC)

We have convened a Trial Steering Committee (TSC) incorporating a Data Management Committee (DMC) to have oversight of the full study, reflecting the allocation of responsibilities set out in the UK Policy Framework for Health and Social Care Research. The charter for the TSC describes the membership, terms of reference, roles, responsibilities, authority, decision-making and relationships of the TSC for this trial, which will also undertake the role of a DMC, including the timing of meetings, methods of providing information to and from the TSC, frequency and format of meetings and relationships with other trial committees. Membership is entirely independent of the trial, and has been set up to represent all key elements required for robust and expert monitoring of the research. The broad aim of the TSC is to act as the oversight body for this trial on behalf of the Sponsor/Funder.

Specifically, the oversight committee will be expected to:

* provide expert oversight of the trial

* maintain confidentiality of all trial information that is not already in the public domain

* make decisions as to the future continuation (or otherwise) of the trial/s

* monitor recruitment rates, losses to follow up and encourage the trial management group (TMG) to develop strategies to deal with any recruitment problems

* review regular reports of the trial from the trials unit (sent on behalf of the TMG)

* assess the impact and relevance of any accumulating external evidence

* monitor completion of CRFs and comment on strategies from TMG to encourage satisfactory completion in the future

* monitor follow-up rates and review strategies from TMG to deal with problems

* censure sites that are deviating from the protocol

* approve any amendments to the protocol, where appropriate

For peer review only

For peer review only - http://bmjopen.bmj.com/site/about/guidelines.xhtml

* approve any proposals by the TMG concerning any change to the design of the trial, including additional sub-studies

* oversee the timely reporting of trial results

* approve / comment on the statistical analysis plan

* approve / comment on the publication policy

* approve early internal requests for release of data or subsets of data or samples including clinical data and stored biological samples

* assume the role of a DMC in assessing at intervals the progress of the RCT, the safety data, and the critical efficacy endpoints, and to recommend to the Sponsor whether to continue, modify, or stop the trial

* assess data quality, including completeness (and by so doing encourage collection of high-quality data)

* consider the ethical implications of any recommendations made by the TSC

* monitor planned sample size assumptions, preferably with regards to

(i) a priori assumptions about the control arm outcome and/or

(ii) emerging differences in clinically relevant subgroups including Black, Asian and other minority ethnic groups who are at greater risk of infection from the SARS-CoV-2 virus, rather than on emerging, unblinded differences between treatment groups, overall:

* suggest additional data analyses if necessary

* advise on protocol modifications proposed by investigators or sponsors (e.g. to inclusion criteria, trial endpoints, or sample size)

* monitor continuing appropriateness of patient-facing information

* monitor compliance with previous TSC recommendations.

Patient & Public Involvement Group

We have a standing Patient and Public Involvement Group for this trial, made up of 12 patients/careers with experience of being treated in hospital with COVID-19 or caring for a relative who has been hospitalised with COVID-19.

For peer review only - http://bmjopen.bmj.com/site/about/guidelines.xhtml

PROTOCOL CONTRIBUTORS

The co-investigators for the trial are David A. Richards, Holly V.R. Sugg, Emma Cockroft, Joanne Cooper, Susan Cruickshank, Claire Hulme, Phillipa Logan, Heather Iles-Smith, G.J. Melendez-Torres, Anne Marie Rafferty, Nigel Reed, Anne-Marie Russell, Maggie Shepherd, Sally J. Singh, Jo Thompson Coon, Susannah Tooze, Stephen Wootton, Rebecca Abbott, Alison Bethel, Siobhan Creanor, Lynne Quinn, Rebecca Whear, with full contact details listed in ‘Key Study Contacts’ section. Jessica Bollen is the study manager and Faye Doris is the patient and public involvement lead to replace Nigel Reed who has stepped down. David Richards wrote the protocol with input from HVRS, Emma Cockcroft, Jo Cooper, Susanne Cruikshank, Faye Doris, Claire Hulme, Philippa Logan, Heather Isles-Smith, G.J. Melendez-Torres, Nigel Reece, Anne Marie Rafferty, Anne-Marie Russell, Maggie Shepherd, Sally Singh, Jo Thompson-Coon, Susannah Tooze and Steve Wooton who reviewed the manuscript and made editorial suggestions. G.J. Melendez-Torres is the senior trial statistician and Claire Hulme the trial economist.

The UKCRC-registered University of Exeter Clinical Trials Unit will contribute to this protocol by will receiving, cleaning and processing trial data following GDPR and data protection guidelines and all relevant Clinical Trial Regulations.

Principal investigators at all sites will acknowledge and conform to the Agreement, and the roles and responsibilities to be undertaken as Principal Investigator at the Participating Site. Site Principal Investigators must be free to participate in the Study, and not be restricted by any third party obligations which might prevent or restrict their performance of the obligations as Principal Investigator. Site Principal Investigators must consider the facilities required for the Study, and be satisfied that the Participating Site can, and will continue to, make appropriate facilities available for the proper performance of the Study. Site Principal Investigators must conduct the Study at the Participating Site in accordance with the Protocol. Site Principal Investigators must also consent to the Sponsor(s), and to any relevant third party providing support, products and/or services to the Study, holding their name and other relevant details on an appropriate database for the purpose of communicating with them in relation to the Study. Finally, Site Principal Investigators must confirm that where they wish to delegate responsibilities to another member of the Study team, that individual will be appropriately qualified for the delegated role, will receive sufficient support and training to fulfil

For peer review only - http://bmjopen.bmj.com/site/about/guidelines.xhtml

that role and all delegated duties will be detailed in a delegation log, or as required by the Sponsor.

This work is supported by National Institute for Health Research and UK Research and Innovation, administered by the MRC: grant number MR/V02776X/1. The Trial Sponsor is the University of Exeter. Neither the funder nor sponsor will have any role or ultimate authority in study design; collection, management, analysis, and interpretation of data; writing of the report; and the decision to submit the report for publication.

The research funder has the responsibility to ensure that there is a proper use of the funds they control. The study is funded as part of the MRC and UKRI COVID Rapid Response Call. The Funder has reviewed the study plan of the research and established that the research is worthwhile, of high scientific quality and represents good value for money. The research funder has assessed the experience and expertise of the Chief Investigator, other key researchers on the programme and has deemed that there is appropriate infrastructure for the research to be carried out.

The funding review process provided feedback on the design of the study plan. The funder plays no further role in the design of this individual study and will have no role in data analysis or interpretation, or writing up of findings of the study. The funder will be sent all outputs prior to dissemination, but has no role in the decision to submit for publication.

The study has been informed by ongoing collaborations between patients, clinicians and researchers, including in a previous NIHR-funded project (ESSENCE) on fundamental nursing care. A version of the lay summary was reviewed by six members of the 'Peninsula Patient Involvement Group', giving feedback on the broad idea of the research as well as specific points on the clarity of the summary. A patient co-applicant has been involved with the development of the proposal. We will ensure there is a patient voice in all discussions and decisions, and will ensure the integration of broader PPI activity in these decisions. We have worked with a wider group of patients with experience of hospitalisation for COVID-19 to inform the co-production of the nursing protocol, development of all patient facing materials, finalising the nursing protocol, adaptions to the nursing protocol after process evaluation, dissemination of findings, including the production of the online training module for nurses. The involvement of patients will be informed by what is possible within the ongoing pandemic, and to suit the situation of interested patients. Involvement will be flexible and responsive to needs. We will adopt high standards of equality, diversity and inclusivity throughout.

For peer review only - http://bmjopen.bmj.com/site/about/guidelines.xhtml

For peer review only

IRAS 288479_COVID-NURSE: RCT for nursing protocol intervention v5.0

KEY WORDS: Nursing; Covid; fundamental care; patient experience;

STUDY FLOW

Patient participant flow chart

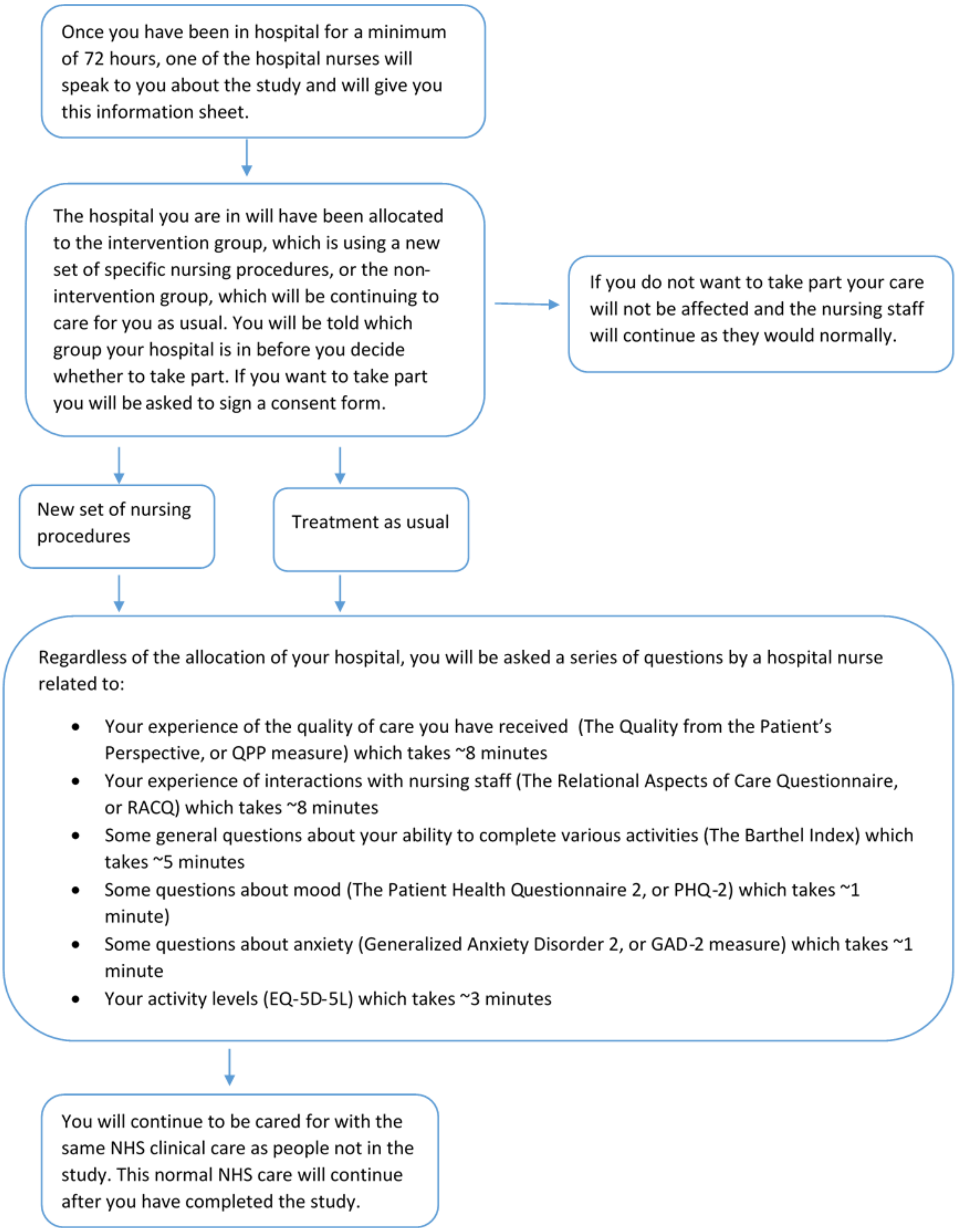

For peer review only - http://bmjopen.bmj.com/site/about/guidelines.xhtml

Simplified study Gantt chart

	Start	20/07/2020	24/08/2020	21/09/2020	19/10/2020	23/11/2020	21/12/2020	25/01/2021	22/02/2021	22/03/2021
	Finish	23/08/2020	20/09/2020	18/10/2020	22/11/2020	20/12/2020	24/01/2021	21/02/2021	21/03/2021	19/04/2021
	Months	1	2	3	4	5	6	7	8	9
Stage 1	Rapid Review	X	X							
	Survey	X	X							
	Protocol co-creation workshops			X						
	Ethics submission	X								
Stage 2	Site recruitment	X	X	X						
	Trial set up	X	X	X						
	Intervention training				X	X	X	X		
	Trial Site Data Collection				X	X	X	X	X	
	Process Evaluation						X	X	X	
	Protocol finalisation								X	
Post trial	Dissemination and implementation								X	X

For peer review only

For peer review only - http://bmjopen.bmj.com/site/about/guidelines.xhtml

STUDY PROTOCOL

COVID-NURSE: development and evaluation of the effects of a COVID-specific fundamental nursing care protocol compared to care as usual on experience of care for non-invasively ventilated patients in hospital with the SARS-CoV-2 virus: protocol for a randomised controlled trial

1 BACKGROUND

Patient experience of care is correlated with safety, clinical effectiveness, care quality, treatment outcomes, costs and reduced service use.(1-6) Nursing care is a key determinant of patient experience.(7, 8) Effective nursing care requires the establishment of compassionate nurse-patient relationships. This facilitates nurses' transactional behaviours to meet the fundamental care needs of patients such as nutrition, hydration, skin integrity, personal care, mobility, hygiene, breathing, elimination and mental wellbeing.(9, 10)

The combination of COVID-19 symptoms and infectiousness of the SARS-CoV-2 virus poses unique challenges for transactional and relational nursing care behaviours. The Canadian SARS outbreak demonstrated that nurses' ability to provide compassionate fundamental care was compromised. "The establishment/maintenance of therapeutic nurse-client relationship required additional time given the barriers of mask, gloves and gowns".p4(11). "Restrictions on visitors were difficult for staff because family members are usually involved in the social, psychological and, to some extent, physical care of patients".p6(12). "Interaction time decreased, and patients began to feel more abandoned".p28(13).

Currently, no evidence-based guidelines exist for nursing hospitalised patients with SARSCoV-2 not invasively ventilated, leading to variations in patient experience, care quality and costs. Our multi-professional team will co-create and evaluate an evidence-based nursing protocol for patients with the SARS-CoV-2 virus, in order to maximise patient experience, care quality, outcomes and costs. We will ensure that this is generalisable to environments such as care homes, patients with other conditions requiring isolation and global health systems. AHPs follow nursing guidelines so this research will be widely applicable.

2 RATIONALE

Investigation into evidence-based transactional and relational nursing care in a heavily compromised care environment is not an objective of current COVID research programmes, despite the relationship between nursing care, patient experience, treatment outcomes and costs. With services now being re-

For peer review only - http://bmjopen.bmj.com/site/about/guidelines.xhtml

opened under the NHS recovery plan, there will be increased pressure on nurses caring for patients with the SARS-CoV-2 virus.

There is only one COVID nursing study (CONNECT – on research priorities post-COVID) but no interventional studies investigating nursing care or patient experience. There are no registered COVID nursing care rapid Cochrane reviews.

Our research aligns with the WHO R&D Roadmap priorities and research gaps, viz: ‘to determine optimal clinical practice strategies to improve the processes of care’. It includes ‘rapid approaches to capture healthcare worker views (surveys, interviews)’ and a rapid ethnograph[y] in healthcare’. It is a short-term study reporting within 9 months, which will make a valuable contribution to the management of the COVID-19 outbreak, with the protocol generated to be made widely available. In recent discussions, the Chief Nursing Officer for England and Chief Nurse at HEE are "very supportive of such exploration into this important field of practice and research."

This research requires the real time pandemic context for delivery of the proposed study design and includes an evaluation of an innovation in health and social care delivery prompted by the pandemic, which can be generalised and actioned in the pandemic context. We have the required interdisciplinary expertise and experience and are building on existing relationships and projects(14, 15).

3 THEORETICAL FRAMEWORK

The theoretical framework is underpinned by the fundamental model of nursing care developed by Kitson and colleagues (9,10), alongside a programme theory and logic model developed directly through three phases of work which we have recently completed as part of this project:

1. Rapid systematic review identifying the evidence for the effectiveness of and barriers to fundamental nursing care procedures in patients with SARS-CoV-2 virus or other conditions requiring isolation, in terms of overall patient experience, care quality, functional ability and treatment outcomes.
2. Survey of innovations in current practice with 2779 respondents; UK-wide survey of nurses and nonregistered care staff, and AHPs working with hospitalised patients with the SARS-CoV-2 virus not invasively ventilated, purposively sampled by region/hospital/ward type to a) obtain existing clinical protocols and b) identify the specific barriers, and innovation strategies being adopted, to meet the fundamental care needs of such patients including their nutrition, hydration, skin integrity, personal care, mobility, hygiene, elimination, breathing, communication and wellbeing needs.
3. Co-creation: we convened three online consensus development panels(17), each including up to ten participants(18): patients (one panel), registered nurses and nonregistered care staff, AHP and medical colleagues (two panels). We followed best practice guidelines(19) to

For peer review only - http://bmjopen.bmj.com/site/about/guidelines.xhtml

facilitate the panels arriving at an agreement on the components of a fundamental nursing care guideline. We set consensus criteria a priori, and facilitated panels by providing them with synthesised review data, survey results and the draft programme theory and logic model to discuss and refine.

These activities have informed the programme theory and logic model which form the basis for our intervention nursing guidelines.

4 RESEARCH QUESTION/AIM(S)

What is the effect of an evidence-based nursing protocol for patients with the SARS-CoV-2 virus, compared to care as usual, in terms of patients' reported experience of transactional and relational nursing care, care quality, treatment outcomes and costs.

4.1 Objectives

To undertake a cluster randomised controlled superiority trial to evaluate an evidence-based nursing protocol for patients with the SARS-CoV-2 virus, compared to care as usual, in terms of patients' reported experience of transactional and relational nursing care, care quality, treatment outcomes and costs.

4.2 Outcome

To establish the effectiveness of an evidence-based nursing protocol for patients with the SARS-CoV-2 virus, compared to care as usual, in terms of patients' reported experience of transactional and relational nursing care, care quality, treatment outcomes and costs.

5 STUDY DESIGN and METHODS of DATA COLLECTION AND DATA ANALYIS

We will undertake a cluster randomised controlled superiority trial, with embedded process and economic evaluations.

We will aim to recruit 14 UK district-general and teaching hospital NHS Trusts (clusters), representing a spread of hospital type, size and ethnic diversity of admitted patients.

Eligible participants will be: patients who are not invasively ventilated, aged ≥18 years, currently hospitalised and being treated for infection with the SARS-CoV-2 virus, or recently discharged after such treatment, and who have received nursing care for a minimum of 72 hours during their admission; registered nurses and nursing care workers including student nurses caring for patients hospitalised and being treated for infection with the SARS-CoV-2 virus. Participants must be able to give informed consent. Translation facilities will be provided for participants unable to understand of speak English.

For peer review only - http://bmjopen.bmj.com/site/about/guidelines.xhtml

The control condition will be care as usual, meaning that control sites will continue to provide nursing care for patients according to their usual care regimes and protocols. The experimental intervention will be care as usual plus the intervention developed through a systematic review, survey and consensus groups of nurses and patients. The intervention consists of three elements:

1. A guideline document (see Appendix 4) highlighting the key barriers to care caused by the COVID-19 care setting and describing initiatives that nurses can use to overcome these barriers, supported by a two-to-three hour 'dip in and out' training programme focussing on explaining the menu of ways in which nurses can adapt their practices to overcome these barriers together with exercises to help nurses learn about these strategies;
2. Reminder notices placed in the COVID-19 clinical environments to help nurses remember these good ideas; and
3. Action from nurse leaders and managers to support nurses' own emotional wellbeing generally and to help implement these practice ideas specifically, supported by leadership and management training included in item 1 above.

The intervention is a menu of good practice suggestions to be used by nurses to overcome barriers to communication, organisation and fundamental nursing care interventions caused by nursing patients with COVID-19 in isolation rooms whilst wearing PPE. All suggestions are merely adaptations of approach, not novel approaches and enhance rather than replace usual nursing care practices.

During intervention implementation, by drawing on iterative process evaluation findings, we will modify our intervention accordingly and embed the new version of the intervention in subsequent experimental intervention sites. We will embed the intervention in intervention sites before data collection via staff education (using the Open University's 'FutureLearn' platform), reminders and leadership-directed strategies, as used successfully in a previous cluster randomised controlled trial to significantly increase handwashing by nurses and care staff. We will monitor intervention adherence and fidelity as part of the process evaluation, amending our educational and leadership strategies accordingly before subsequent implementation in sites.

We will undertake the trial during 2021, with data collected from patient participants admitted for treatment of SARS-CoV-2 infection and staff caring for them. Ideally, following our original three-wave rapid cycle design, the cluster sites would have participated in one of three cycles at six weekly intervals with each cycle running for six weeks. However, experience enrolling wave one sites during October 2020-January 2021 has taught us that this ideal situation is subject to considerable stress as sites adapt to a rapidly changing situation on the ground, facing significant clinical and research pressures. Therefore, we have redesigned the study to a simple cluster randomised trial with sites randomised without matched pairing. In order to gather and respond to important process evaluation

For peer review only - http://bmjopen.bmj.com/site/about/guidelines.xhtml

data, we will analyse process data on fidelity and adherence on a rolling basis to enable us to make any necessary adaptations to the nursing intervention.

Using published formulae by Hayes & Moulton (16) we calculated our sample size based on an estimate of the minimum clinically important difference for the QPP of 0.2 and the typical within-unit standard deviation (0.6), supplied by the measure developers, and an ICC of 0.02. We have examined a number of potential scenarios taking into account different cluster sizes and different between-cluster standard deviation values informed by between-country differences on measures, estimated to provide 90% and 80% power. Based on conversations on feasibility with NHS clinical leaders, with a cluster size of 60 the trial would generate over 80% power with 6 clusters per arm and 90% power with 7 clusters in each arm. However, because power calculations in cluster trials with a small number of clusters can be especially sensitive to the approximation used for degrees of freedom, we used a range of alternative approaches (no small sample adjustment, Satterthwaite approximation, and Kenward-Rogers approximation; cluster-level linear regression) to explore the robustness of the sample size calculations. While these tended to estimate reduced power at each number of clusters compared to a formula-based power calculation, an estimate of 14 clusters (7 per arm) generated power of greater than 80% in every case. This number of clusters is fairly robust to number of patients per cluster. Even assuming under-recruitment leading to 50 patients per site, power is maintained at greater than 80%.

We will recruit cluster sites via the NHS chief nurse and clinical research networks. We will also use other strategies to publicise the study such as social media, and word of mouth and email communication among colleagues, as well as promotion on the university and study webpages. During stage 1 of the study, we monitored available data from Public Health England on case and hospitalisation rates by nation, region and local authority in order to identify recruitment sites where there were sufficient potential participants. We use this data to prepare sites for involvement. In order to maximise participant numbers, we will recruit sites as near as possible in time to the randomisation point, when sufficient participants are likely to be available for our sample. Clinical research nurses or appropriately trained and qualified members of the sites research team will recruit patient participants from admitted patients according to the eligibility criteria above.

To ensure allocation concealment, randomisation will be performed by an unblinded statistician who has no role in site recruitment or data analysis; only the unblinded statistician will have access to the allocation list. Randomisation will use a static blocked list.

The co-primary outcomes are patient-reported experience measures addressing transactional nursing care from the Quality from the Patients Perspective (QPP) measure (20) and the Relational Aspects of Care (RACQ) measure (21).

For peer review only - http://bmjopen.bmj.com/site/about/guidelines.xhtml

Our secondary outcomes will be: three elements of ward based care quality collected by the United Kingdom National Health Service Ward to Board dashboard (22) – prevalence of level 3 and 4 pressure sores, frequency of patient falls, frequency of medication errors; functional ability by the Modified Barthel ADL Index (23), treatment outcomes by the WHO Clinical Progression Scale (24), depression by the PHQ-2 (25), anxiety by the GAD-2 (26), and nurse outcomes by the Measure of Moral Distress for Health Care Professionals (27).

We will collect outcome data from patient participants after they have received nursing care for a minimum of 72 hours. All outcome measures will be applied to all participants equally and collected by clinical research nurses or appropriately trained and qualified members research staff. It will not be possible to blind research nurses, clinicians or patient participants to allocation. Statistical analysts will be blind to group allocation. We will collect outcome data either face to face or by video/audio technology according to the infection control procedures in place and participant preference if the participant is still hospitalised, or by telephone if they been discharged. Some data for the participants will need to be collected from the patients notes; the WHO Clinical Progression Scale, which requires information regarding treatments depending on condition severity, and the health economic evaluation questionnaire for Hospital Care Use. Additionally the patients' height and weight may also be taken from their notes. We will collect outcome data from nurses either face to face in their hospital, or over the phone, according to their preference. Paper copies of the CRF can be supplied for nursing care staff if this is their preference. This will expedite data collection and enable night staff to more easily participate in the trial. Site level Ward to Board data will be collected from routine hospital statistics.

We will analyse numerical data in STATA v.16. Our primary analysis for patient and nurse outcomes will use a standard generalised linear mixed-effect model with appropriate small-sample adjustment to account for the small number of clusters, and adjusting for ethnicity of the patient sample at cluster level and, where patient outcomes are tested, at patient level as well. Sensitivity analyses for patient and nurse outcomes will use variance-weighted cluster-level summaries with adjustment for ethnicity based on 2011 census and hospital postcode and for research intensity. Analysis of hospital outcomes will use rate ratios. Secondly, and focusing on patient-level outcomes, we will estimate intervention effectiveness in an exploratory analysis. This analysis will use generalised linear mixed-effects models to model time trends. Analysis will include time from implementation, reflecting growing adaptation and expertise, as well as calendar time, reflecting the course of the SARS-CoV-2 epidemic. We will also undertake a planned sub-group analysis to compare the impact of patient-level ethnicity on our primary and secondary outcomes.

We will evaluate the impact of nurses' and care staffs' fidelity to the clinical intervention, intervention mechanisms and context of care delivery on outcomes using: a bespoke instrument for capturing fidelity developed alongside the intervention to reflect the intervention components; specific

For peer review only - http://bmjopen.bmj.com/site/about/guidelines.xhtml

mechanism instruments appropriate to our programme theory; the Culture of Care Barometer Questionnaire (28); NOMAD questionnaire (29); contextual data on patient acuity, staffing ratios, nurses/care staff education levels, seniority and year of qualification, overall site training records from the FutureLearn course site, anonymised and collated by a University of Exeter course Learning Manager', and other contextual programme theory derived variables. We will conduct rolling iterative analyses of process evaluation data on fidelity to the intervention, intervention mechanisms, and intervention utilisation to evaluate intervention component fidelity and implementation. We will subsequently amend the intervention based on this data before embedding the new protocol in intervention sites.

We will conduct semi-structured qualitative interviews with a purpose sample of nurses and care staff, using a topic guide derived from the content of the intervention and our analysis of quantitative data, to explore their views on the mechanisms, impact and acceptability of the intervention. This method will enable us to investigate the meaning of participants' responses, both exploring views on our predefined topics of interest and eliciting more detail on any emerging themes. Interviews will be conducted using telephone or online audio-conference methods, with participants' consent, we will audio-record interviews and transcribe interviews verbatim. We will use NVivo10 to organise the data and analyse data using Framework analysis to allow for the combination of inductive and deductive approaches in our development of themes. We will use joint displays to integrate the qualitative data with the quantitative outcome data in a mixed methods analysis to explore and explain variation in the outcome data.

We will conduct within trial cost effectiveness analysis by adopting the NHS perspective with the main outcome measure the Quality-adjusted life year (QALY) derived from the EQ-5D-5L applying the NICE threshold of £20,000 per QALY. Cost data will include length of stay, other unit costs derived from national sources including Personal Social Services Research Unit and NHS Reference Costs with non-parametric bootstrapping to quantify uncertainty. We will present outputs as ICERs, cost effectiveness acceptability curves and expected net benefit.

Day to day management of the trial and study will be by the chief-investigator, other co-investigators and a research manager. We will set up an independent combined Trial Steering and Data Management Committee (TSC/DMC) and a Patient and Public Involvement group. The TSC/DMC will review serious adverse event reports thought to be related to the intervention by the PI and/ or the CI. We will adopt high standards of equality, diversity and inclusivity throughout, to particularly ensure that we have input from people of colour, including from Black, Asian and other minority ethnic groups, who are at greater risk from infection with the SARS-CoV-2 virus.

For peer review only - http://bmjopen.bmj.com/site/about/guidelines.xhtml

The proposed study has been informed by ongoing collaborations between patients, clinicians and researchers, including in a previous NIHR-funded project (ESSENCE) on fundamental nursing care. A version of the lay summary was reviewed by six members of the 'Peninsula Patient Involvement Group', giving feedback on the broad idea of the research as well as specific points on the clarity of the summary. A patient co-applicant has been involved with the development of the proposal. We will ensure there is a patient voice in all discussions and decisions, and will ensure the integration of broader PPI activity in these decisions. We will work with a wider group of patients with experience of hospitalisation for COVID-19 to inform the co-production of the nursing protocol, development of all patient facing materials, finalising the nursing protocol, adaptions to the nursing protocol after process evaluation, dissemination of findings, including the production of an online training module for nurses on the future learn platform. The involvement of patients will be informed by what is possible within the ongoing pandemic, and to suit the situation of interested patients. Involvement will be flexible and responsive to needs.

We will conduct this trial in such a way as to protect the human rights and dignity of the participants, as reflected in the Declaration of Helsinki 2013. For the stage 1 survey, we obtained governance approval from the Health Research Authority (IRAS project ID: 287288, protocol number 1920/31) and ethical approval from the University of Exeter research ethics committee (no: 20/07/256). Approvals for stage 2 have been submitted and approved by the HRA UK Health Departments Research Ethics Committee (IRAS project ID 288479; REC reference: 20/LO/1043). Participants will not receive any financial inducement to participate. We will invite eligible patients and nurses to participate in data collection and will only analyse data from participants that individually consent. Participants may withdraw from the study at any time without prejudicing their treatment, care or employment. We will conform to Good Clinical Practice Guidelines, data protection and freedom of information acts. All participant and research data will be stored securely and anonymised wherever possible. All identifiable participant information will be stored separately to questionnaire data which will be coded by an ID number only. No published material will contain identifiable participant information.

Participant data will be recorded electronically and stored via REDCap v9.1, a browser-based, metadata-driven EDC software and workflow methodology for designing clinical and translational research databases. Any data from nursing staff collected on the hard copies of the CRF will be transcribed by site research staff into the electronic CRF (REDCap). The hard CRF copies will be considered source data and will be archived for 10 years at site and destroyed according to the sites SOP's at this time. All data is stored securely hosted in a secure cloud based infrastructure on servers in the European Economic Area (EEA) at a data centre in Frankfurt, Germany with a daily backup to servers hosted in Dublin, Ireland. REDCap Cloud complies with GDPR.

For peer review only - http://bmjopen.bmj.com/site/about/guidelines.xhtml

All communications with REDCap will be over an encrypted SSL connection. Access to REDCap for each user is via a unique username/password and roles are used to limit access to the minimum necessary for that role. Users accounts are managed by CTU. Personal data collected at participating sites will be stored in a GDPR compliant manner in-line with local guidelines. Personal data including participant's contact information may be transferred from participating sites to authorised members of the research team including study team members at the CTU. Transfer and storage of personal data will be minimised and will be done in a secure manner in compliance with the GDPR.

Access to confidential research data on NHS or University computers will be restricted to the study team using appropriate controls. The investigators and relevant authorities will have access to the trial dataset. Furthermore, we will store anonymised research data and outputs in the University of Exeter's Open Research Exeter repository (https://ore.exeter.ac.uk/repository/) in order to facilitate open access to, and the impact of, our research.

We will disseminate our results through publication in peer reviewed scientific journals, our study website and other press and online media. Manuscript authors will be those considered to have made a substantive intellectual contribution to the study. The investigators and relevant authorities will have access to the trial dataset. Furthermore, we will store anonymised research data and outputs in the University of Exeter's Open Research Exeter repository (https://ore.exeter.ac.uk/repository/) in order to facilitate open access to, and the impact of, our research.

6 STUDY SETTING

Data will be collected at study sites. As we are recruiting and allocating at the level of sites, potential participants will be already in place at the NHS trial sites or will have recently been discharged. Potential participants will be identified and asked to take part by the site research nurse or appropriately trained and qualified members of the research team based on their status as detailed in the inclusion criteria. After potential patient participants have been admitted to hospital for a minimum of 72 hours, if eligible to take part they will first be approached by a research nurse. This may either be in person at the site or by telephone if the participant has been discharged. At the site, the approaching nurse will ensure the patient is awake and able to discuss the trial, and will assess capacity to consent. If the patient is telephoned after discharge, and consent has not been received whilst they were in hospital, the nurse would ensure it was a suitable time for the potential participant to discuss the study, and ascertain if they would be willing to read the PIS. The PIS and consent forms would be sent to the patient if they agree to receive them with a follow up phone call to check if they were willing to participate and had any questions. If the patient agrees to participate verbally they will be asked to return a consent form in an included return envelope (and keep one for their records) and a time and date will be arranged for the patient to be called back. Once the consent form has been

For peer review only - http://bmjopen.bmj.com/site/about/guidelines.xhtml

For peer review only

received at the site, the planned phone call will take place and the data and contact details collected. If the consent form hasn't arrived before the planned call, the call will still be made to check to see if the patient is still willing to participate and if the consent form is on its way. A new date and time to call will be arranged if the patient is still wanting to participate. Nurse participants will be recruited through posters displayed on wards to raise awareness of the trial, and approaches by the research nurse or other appropriately trained and qualified individuals and once they have given consent to take part will provide their data to site research nurses or other appropriately trained and qualified individuals either by face to face or the paper copies of the CRF's.

This is a multicentre study. Sites must be acute hospitals with patients admitted for a minimum of 72 hours before recruitment. Patients should be non-ventilated, and nursed as if they have SARS-CoV-2 virus (i.e. they are eligible if SARS-CoV-2 virus is suspected or positively diagnosed).

Participants are recruited and assessed by research nurses or other appropriately trained and qualified individuals at each study site.

7 SAMPLE AND RECRUITMENT

7.1 Eligibility Criteria

7.1.1 Inclusion criteria

Patients are eligible if they are aged ≥18 years, currently hospitalised, or have been recently discharged, treated for infection with the SARS-CoV-2 virus and who have received hospital nursing care for a minimum of 72 hours without invasive ventilation during their current or recent admission.

Registered nurses, student nurses and nursing care workers caring for patients hospitalised and treated for infection with the SARS-CoV-2 virus. Participants must be able to give informed consent. Translation facilities for participants unable to understand and speak English will be provided.

7.1.2 Exclusion criteria

Ineligible participants are participants not able to give informed consent; patients who are currently invasively ventilated; patients aged <18 years; patients not currently hospitalised and treated for infection with the SARS-CoV-2 virus; patients who have not received nursing care for a minimum of 72 hours during their current or recent admission; and registered nurses and nursing care workers not caring for patients hospitalised and treated for infection with the SARS-CoV-2 virus.

7.2 Sampling

As we are recruiting and allocating at the level of sites, potential participants will be already in place at the NHS trial sites or will have been recently discharged. Potential participants will be identified and

For peer review only

For peer review only - http://bmjopen.bmj.com/site/about/guidelines.xhtml

asked to take part by the site research nurse or appropriately trained and qualified members of the sites research team based on their status as detailed in the inclusion criteria.

7.2.1 Size of sample

The total UK sample size is 1540; 840 patient participants and 700 nursing participants.

7.2.2 Justification of target sample size

Using published formulae by Hayes & Moulton (2017) we have calculated our sample size based on an estimate of the minimum clinically important difference for the QPP, and the typical within-unit standard deviation (0.6) supplied by the measure developers. We have examined a number of potential scenarios taking into account different cluster sizes and different between-cluster standard deviation values informed by between-country differences on measures, estimated to provide 90% and 80% power. We have selected 90% power and a mean cluster size of 60 participants based on conversations on feasibility with NHS clinical leaders. As such, we will need to recruit a total of nine clusters per allocated arm (18 in total) to detect a between-group standard deviation of 0.082 in the QPP with a two-sided significance level of 0.05. Were we to have a recruitment shortfall of 33% per cluster, a total of 10.2 clusters per arm would be required for 90% power. However, with the originally planned number of clusters, we will still attain 80% power. While our ambition is to recruit nine pairs of hospitals, this may not be possible given this rapidly changing situation. However, a trial with as few as six pairs, but with 80 patients per site and under our base-case estimate of coefficient of variation would still yield approximately 80% power. Under a lower coefficient of variation, 80% power will be achieved with six pairs and about 50 patients per arm.

7.3 Recruitment

As we are recruiting and allocating at the level of sites, potential participants will be already in place at the NHS trial sites or will have been recently discharged. Potential participants will be identified and asked to take part by the site research nurse or appropriately trained and qualified members of the sites research team based on their status as detailed in the inclusion criteria. Patient length of stay and status as having suspected or confirmed COVID, as well as ventilation status (non-invasive/invasive ventilation) will be identified for eligibility before patient participants are approached to take part in the trial.

7.3.1 Sample identification

After potential patient participants have been admitted to hospital for a minimum of 72 hours, if eligible to take part they will first be approached by a research nurse or appropriately trained and qualified individual at each individual site once allocation to the trial arm has taken place. This will take place in person if the participant is a current inpatient, or by telephone if they have been recently discharged.

For peer review only

For peer review only - http://bmjopen.bmj.com/site/about/guidelines.xhtml

The approaching nurse will ensure the patient is awake and able to discuss the trial, and will also assess capacity to consent. If the patient participant is telephoned after discharge, and consent has not been received whilst they were in hospital, the nurse would ensure it was a suitable time for the participant to discuss the study, and ascertain if they would be willing to read the PIS. The PIS and consent forms would be sent to the patient if they agree to receive them with a follow up phone call to check if they were willing to participate and had any questions. If the patient agrees to participate verbally they will be asked to return a consent form in an included return envelope (and keep one for their records) and a time and date will be arranged for the patient to be called back. Once the consent form has been received at the site, the planned phone call will take place and the data and contact details collected. If the consent form hasn't arrived before the planned call, the call will still be made to check to see if the patient is still willing to participate and if the consent form is on its way. A new date and time to call will be arranged if the patient is still wanting to participate.

Nurses will be identified for eligibility based on their status at the participating hospital as caring for patients with confirmed or suspected COVID. Nurse participants will be recruited through posters displayed on wards to raise awareness of the trial and approached from the research teams at site. These posters will be developed with input from patients and clinicians within our research team and the PPI group.

We will invite eligible patients and nurses to participate in data collection, via site research nurses or appropriately trained and qualified individual, and will only collect data from participants that individually consent. Paper copies of the CRF can be supplied for nursing care staff if this is their preference. This will expedite data collection and enable night staff to more easily participate in the trial. Participants may withdraw from the study at any time without prejudicing their treatment, care or employment. We will conform to Good Clinical Practice Guidelines, data protection and Freedom of Information acts. All participant and research data will be stored securely and anonymised wherever possible. All identifiable participant information will be stored separately to questionnaire data which will be coded by an ID number only. No published material will contain identifiable participant information. Information and data storage, and data use will be included in the participant information sheet (see Appendix 5 & 6).

Site research nurses or appropriately trained and qualified individuals will be used due to the ongoing pandemic situation, the need to minimise non-essential staff on wards, and the increased challenges faced with PPE use, travel and enhanced infection control procedures.

7.3.2 Consent

Capacity to consent will be assessed by the research nurse or appropriately trained and qualified individual when first approaching potential patient participants. We will not be recruiting patients who

For peer review only - http://bmjopen.bmj.com/site/about/guidelines.xhtml

lack capacity to consent. Patients interested in participating in the project will first be invited to read the participant information sheet. This will be done in the manner to best suit the patient and infection control procedures at site and if the patient has been discharged, the PIS will be sent in the post with consent forms. Therefore they can be given a paper copy of the form, have it presented electronically, with the aid of an iPad, or it can be read to them. Those who are interested in participating in the project will be invited to sign a consent form. Before deciding whether to take part, potential participants will be encouraged to ask questions, researchers will ensure that the participant fully understands the purpose of the research, risks associated with the intervention, obligations of participation and his/her right to withdraw at any time. Although verbal consent may be obtained by site research staff from discharged potential patient participants on the telephone, consent forms must be fully completed, signed and returned before patient data can be included in the trial databases.

Nurses wishing to participate in the study will also be asked to sign a consent form. Prior to this they will read the appropriate participant information sheet, have time to consider participation and any risks or benefits associated, have the opportunity to ask questions regarding the study and have them answered.

Participants will be offered and allowed as much time as they need to ask questions and consider taking part. If data is being collected from patient participants during their hospital stay, consent will be taken immediately prior to collecting outcome measures if participants are willing, due to the difficulties in donning/doffing PPE and increased resourcing pressures due to the fast moving Covid situation. Participant information sheets are available in Appendix 5 (patients), Appendix 6 (nurses). Consent forms are available in Appendix 7 (patients), Appendix 8 (nurses).

7.4 Patient safety and reporting

The recording and reporting of non-serious Adverse Events (AEs) in this study is not required.

Serious Adverse Events (SAEs), are defined as the following:

- Results in death
- Is life threatening; an event in which the patient was at risk if death at the time of the event
- Requires hospitalisation or prolongation of existing hospitalisation
- Results in persistent or significant disability or incapacity
- Consists of a congenital anomaly or birth defect
- Is otherwise considered medically significant by the investigator

The following are expected to occur in the COVID-NURSE trial and are not reportable as SAEs:

For peer review only

For peer review only - http://bmjopen.bmj.com/site/about/guidelines.xhtml

- A significant deterioration in clinical status due to COVID-19 infection requiring a change in medical treatment such as invasive ventilation
- Prolongation of hospital stay due to no suitable place once discharged from hospital

All other SAE's will be reported to Exeter CTU within 24 hours of the Principal Investigator becoming aware of the event. Fatal and life-threatening events experienced by participants while participating in the study are unlikely to be caused by participation in this trial

There are no SAEs which are considered expected for the trial intervention. Therefore if the SAE is considered related to the intervention it will be classed as a related unexpected serious adverse event 'RUSAE'. RUSAEs will be reported to the Sponsor and REC that granted the ethical approval within 15 days of ExeCTU becoming aware of the event. RUSAE's will also be reported to the TSC. Any fatal or life threatening RUSAE will be reported to the REC and sponsor within 7 days. Fatal and life-threatening events experienced by participants while participating in the study are unlikely yp be caused by participation in this trial.

8 ETHICAL AND REGULATORY CONSIDERATIONS

8.1 Assessment and management of risk

Currently, no international evidence-based guidelines exist for nursing hospitalised patients with SARS-CoV-2 not invasively ventilated, leading to potential variations in patient experience, treatment outcomes, care quality and costs. Although individual nurses, teams and organisations have rapidly adapted their procedures to nurse these patients, compared to usual nursing care, we do not know how to optimise nursing care for the specific challenges of nursing COVID-19 patients, nor how effective these adaptations are in terms of patient experience, care quality, treatment outcomes and costs.

In the COVID-NURSE trial, our objective is to undertake a randomised controlled superiority trial to evaluate an evidence-based nursing protocol for patients with the SARS-CoV-2 virus, compared to care as usual, in terms of patients' reported experience of transactional and relational nursing care, care quality, treatment outcomes and costs. This will have an embedded process evaluation during the trial, and an effectiveness and economic evaluation at the conclusion of the trial.

This design and research proposal has been developed with an expert group of co-investigators including patients, NIHR nursing advocates, NIHR AHP training advocates, PPI specialists, methodologists, researchers, trial experts, nurse educators, implementation and behavioural science specialists, process evaluators and health economists. The intervention is being developed from a nurse-focussed survey of care innovations (IRAS project ID 287288: Covid-Nurse: a staff survey of

For peer review only - http://bmjopen.bmj.com/site/about/guidelines.xhtml

meeting SARS-CoV-2 patients' fundamental nursing care needs), a systematic review of current evidence, and co-designed with groups of experts by experience with both nursing and patient groups.

The intervention arm will involve 'care as usual' plus the clinical protocol we developed as described above. After implementation, and drawing on process evaluation findings, we will modify our clinical protocol accordingly and embed the new version of the protocol in the subsequent experimental intervention sites. We will embed the protocol in intervention sites via staff education (using the Open University's 'FutureLearn' platform), reminder posters and leadership-directed strategies, as used successfully in a previous cluster randomised controlled trial to significantly increase handwashing by nurses and care staff. We will monitor intervention adherence and fidelity as part of the process evaluation, amending our educational and leadership strategies accordingly.

In order to maximise participant numbers, we will recruit sites as near as possible in time to the randomisation point, when sufficient participants are likely to be available for our sample. Clinical research nurses or appropriately trained and qualified members of the sites research team will recruit patient participants according to clear eligibility criteria.

Nurses and patients at each site will be individually asked to consent to individual outcome data being collected as part of the study. It will be made explicitly clear that patient participants are consenting to giving their individual outcome data, but they will be taking part in the study as we are recruiting at the level of the site, rather than at the level of the individual participant. If a patient is in an intervention site hospital, they will receive the intervention but have the choice to consent or not consent to their individual outcome data being collected.

Recruitment posters will be distributed at sites to give information about the study and to invite people to take part. Research nurses or appropriately trained and qualified individuals at each site will approach potential participants at each recruited site with a clear script to minimise coercion. Participant information sheets will be given to potential participants, tailored to their role (patient or nurse), and these will be available in different formats (online or paper) and language options appropriate to the local site. Recruitment material will make no therapeutic claims and inducement will be avoided. No personal data will be collected at this point until potential participants have consented to take part.

We are including patients who are not currently invasively ventilated, aged ≥18 years, currently hospitalised, or who have recently been discharged, and treated for infection with the SARS-CoV-2 virus and who have received nursing care for a minimum of 72 hours during their current admission; registered nurses and nursing care workers caring for patients hospitalised and treated for infection with the SARS-CoV-2 virus. Participants must be able to give informed consent. Translation facilities for participants unable to understand and speak English will be provided.

For peer review only

For peer review only - http://bmjopen.bmj.com/site/about/guidelines.xhtml

We have set up an independent combined Trial Steering and Data Management Committee and a Patient and Public Involvement group. We have adopted high standards of equality, diversity and inclusivity throughout, to particularly ensure that we have input from people of colour, including from Black, Asian and other minority ethnic groups, who are at greater risk from infection with the SARS-CoV-2 virus.

We will ensure that research nurses or appropriately trained and qualified members of the sites research team understand the consent process, have the ability to assess capability and capacity and understand the ethical principles underpinning informed consent.

Similarly, through clear instruction materials, we will ensure that potential participants understand the purpose and nature of the research, what the research involves, its benefits and lack of benefits, risks and burdens, and the alternatives to taking part. We will ensure through instruction materials that potential participants have the ability to retain the information long enough to make an effective decision, can make a free choice, and are capable of making this particular decision at the time it needs to be made.

This study will tell us what really works for patients in terms of their experiences of care. The study will be complete with results available to share by November 2021. We will publish the results from the clinical trial on the study website and using other media such as professional journals, academic journals and through the press and online media. We will make our results are freely available so that nurses throughout the UK and globally can use our protocol for COVID-19 patients.

We will ensure that our results are totally anonymised and will not identify anyone who has taken part in the trial. Data will be collected and stored in accordance with the Data Protection Act 2018. Personally identifiable data will be stored separately to research data (e.g. completed questionnaires). Where data is disseminated (e.g. via report, presentation or publication), it will be anonymised. We will align all confidentiality and data handling with the Caldicott Principles ethical framework.

Participants will be identified by a unique study ID. Personal identifiable data will be collected only if absolutely required and will be stored separately to research data and will be destroyed as per applicable regulations when the project is concluded. Data will be managed by the UKCRC registered Exeter Clinical Trials Unit (ExeCTU) following GDPR and all relevant Clinical Trial Regulations. All data will be anonymised prior to publication. Data will be collected and stored in accordance with the Data Protection Act 2018 and ICH GCP E6 R2. ExeCTU use the Redcap Cloud Electronic Data Capture System to collect all case report data which includes data obtained from the patients' medical records. This system is validated to ISO27001 standards, backed up and maintained in Europe. This system is fully compliant to GDPR regulations and managed by ExeCTU. Any additional study data will be stored and backed up on the secure ExeCTU servers, maintained by ExeCTU. Only Research

For peer review only - http://bmjopen.bmj.com/site/about/guidelines.xhtml

nurses or suitably qualified and trained individuals will access the patients' notes to gather the required information for the COVID-NURSE trial. Data collected at site on paper such as participant contact information and consent forms (conforming to local policies on infection control), will be stored and archived at site. Contact detail information forms held at sites will be destroyed at the end of the study, once all participants have been notified of the results and the CI or delegated individual instructs sites to destroy the forms. Consent forms and any paper CRF's completed by nurses will be archived for 10 years at site and destroyed according to the sites SOP's at this time. Data will be cleaned and validated appropriately and a full Data Protection Impact Assessment (DPIA) will be undertaken along with a comprehensive Data Management Plan (DMP) before the first patient is recruited.

Where data are disseminated (e.g. via report, presentation or publication), they will be anonymised. We will align all confidentiality and data handling with the Caldicott Principles ethical framework. Anonymised data will be stored indefinitely on a research data storage system provided by the University of Exeter called Open access Research Exeter (ORE) for archiving (http://www.exeter.ac.uk/research/openresearch/policies/ore/).

All results will be shared as per our protocols. Specifically, participants will be informed of the study findings via our website, which they will be directed towards in the PIS

8.2 Research Ethics Committee (REC) and other Regulatory review & reports

As part of our submission to the HRA (IRAS 288479), have sought concurrent REC review under the fast-tracked Covid research procedures and have received a favourable opinion from the HRA REC review process for the study protocol, informed consent forms and appendices.

Substantial amendments that require review by NHS REC will not be implemented until that review is in place and other mechanisms are in place to implement at site. All correspondence with the REC will be retained. The Chief Investigator will produce reports as required by the REC, and the Chief Investigator will notify the REC of the end of the study. As the study takes place over 9 months, we will follow REC and HRA guidance on final reports and closure notifications at the end of the study period.

Regulatory Review & Compliance

Before any site can enrol patients into the study, the Chief Investigator or designee will ensure that appropriate approvals from participating NHS organisations are in place and comply with the relevant guidance.

For any amendment to the study, the Chief Investigator or designee, in agreement with the sponsor will submit information to the appropriate body in order for them to issue approval for the amendment.

For peer review only - http://bmjopen.bmj.com/site/about/guidelines.xhtml

The Chief Investigator or designee will work with sites (R&D departments at NHS sites as well as the study delivery team) so they can put the necessary arrangements in place to implement the amendment to confirm their support for the study as amended.

Amendments

If the sponsor wishes to make a substantial amendment to the REC application or the supporting documents, the sponsor will submit a valid notice of amendment to the REC for consideration within regulatory timescales. The sponsor will decide whether an amendment is substantial or non-substantial for the purposes of submission to the REC.

Amendments will also be notified to the national coordinating function of the UK country where the lead NHS R&D office is based and communicated to the participating organisations (R&D office and local research team) departments of participating sites to assess whether the amendment affects the NHS permission for that site. Some amendments may be considered to be non-substantial for the purposes of REC but still need to be notified to NHS R&D (e.g. a change to the funding arrangements).

Amendments will be made in accordance with the HRA procedures clearly outlined in the IRAS submission process. The Chief Investigator will be responsible, in consultation with the Sponsor, for whether the protocol needs to be amended. The decision on whether amendments are substantial or non-substantial are the responsibility of the Sponsor.

Amendment history will be tracked and recorded in detail using the Exeter CTU standard operating procedures, in order to maintain record fidelity and ensure the latest version of the protocol is in use.

8.3 Peer review

The original funded application was reviewed as part of the UKRI/NIHR application process, including standard peer review, generating feedback to which applicants responded before a final decision to fund was received. The relevant protocols were reviewed and agreed by members of the research team, including within the Chief Investigator's home institution, in accordance with Exeter CTU standard operating procedures.

8.4 Patient & Public Involvement

This study has been informed by ongoing collaborations between patients, clinicians and researchers, including in a previous NIHR-funded project (ESSENCE) on fundamental nursing care. A version of the lay summary in the original funding submission was reviewed by six members of the 'Peninsula Patient Involvement Group', giving feedback on the broad idea of the research as well as specific points on the clarity of the summary. A patient co-applicant has been involved with the development and submission of the proposal.

For peer review only

For peer review only - http://bmjopen.bmj.com/site/about/guidelines.xhtml

We have a standing Patient and Public Involvement Group for this trial, made up of 12 patients/careers with experience of being treated in hospital with COVID-19 or caring for a relative who has. This group has to date informed:

- the research protocol, including methods for approaching and sharing information with potential participants;
- co-produced the nursing protocol and training which will be used in this research;
- Members of the PPI group have commented and improved on earlier drafts of the Participant Information Sheets and consent forms.

We continue to meet with and involve this group throughout the project, with further plans to:

- discuss possible adaptions to the protocol after process evaluation.
- discuss the dissemination of findings and long term implementation of the findings.

8.5 Protocol compliance

Accidental protocol deviations can happen at any time. They will be adequately documented on the relevant forms according to the ExeCTU SOPs and reported to the Chief Investigator and Sponsor immediately. Deviations from the protocol which are found to frequently recur are not acceptable, will require immediate action and could potentially be classified as a serious breach.

8.6 Data protection and patient confidentiality

We will conduct this trial in such a way as to protect the human rights and dignity of the participants, as reflected in the Declaration of Helsinki 2013. For the stage 1 survey, we obtained governance approval from the Health Research Authority (IRAS project ID: 287288, protocol number 1920/31) and ethical approval from the University of Exeter research ethics committee (no: 20/07/256). Approvals for stage 2 were submitted to the HRA UK Health Departments Research Ethics Committee (IRAS project ID 288479; REC reference: 20/LO/1043) to obtain HRA Approval which was granted on 11th November 2020.

Participants will not receive any financial inducement to participate. We will invite eligible patients and nurses to participate in data collection and will only analyse data from participants that individually consent. The research nurses or suitability qualified and trained individuals accessing medical notes for the aspects of data outlined above will do so using the Caldicott principals and only collecting what is strictly necessary for the COVUID-NURSE trial. The data will be entered straight onto the REDCap electronic data system, keeping the data as securely as possible. Being NHS research staff they will

For peer review only - http://bmjopen.bmj.com/site/about/guidelines.xhtml

be aware of their responsibilities and comply within the law. Participants may withdraw from the study at any time without prejudicing their treatment, care or employment.

We will conform to Good Clinical Practice Guidelines, data protection and freedom of information acts. All participant and research data will be stored securely and anonymised wherever possible. All identifiable participant information will be stored separately to questionnaire data which will be coded by an ID number only. No published material will contain identifiable participant information. Information and data storage, and data use will be include in the participant information sheet (see Appendices 5 & 6).

8.7 Indemnity

1. Arrangements made for insurance and/or indemnity to meet the potential legal liability of the sponsor for harm to participants arising from the management of the research:

   The University of Exeter will sponsor this research and provide insurance to meet potential legal liability for harm to participants arising from the management of the research.

2. Arrangements made for insurance and/ or indemnity to meet the potential legal liability of the sponsor or employer for harm to participants arising from the design of the research:

   The University of Exeter will sponsor this research and provide insurance to meet potential legal liability for harm to participants arising from the design of the research.

3. Arrangements made for insurance and/ or indemnity to meet the potential legal liability of investigators/collaborators arising from harm to participants in the conduct of the research:

   NHS staff will be recruited as participants at NHS sites and will be covered by their own professional indemnity. Non-NHS insurance/indemnity will be provided through the University of Exeter: The University of Exeter will sponsor this research and provide insurance to meet potential legal liability for harm to participants arising from the conduct of the research. See note in Appendix 9.

The sponsor has not made arrangements for payment of compensation in the event of harm to the research participants where no legal liability arises.

Ipads are to be provided to sites for the purposes of the study, but there will be no arrangements made for insurance and/ or indemnity to meet the potential legal liability arising in relation to the equipment (e.g. loss, damage, maintenance responsibilities for the equipment itself, harm to participants or site staff arising from the use of the equipment) as these risks are deemed to be low.

For peer review only - http://bmjopen.bmj.com/site/about/guidelines.xhtml

8.8 Access to the final study dataset

The Exeter CTU will have access to the final study dataset. The TSC will arbitrate formal requests to review the final study dataset from anyone outside the study team, although it is not expected that individual sites will be allowed to view this in order to ensure that the overall results are not disclosed by an individual study site prior to the main publication. It is envisaged that that dataset may be used for secondary analysis in line with NIHR advice, and this is included as a point for consent within the participant consent forms (see Appendices 6 and 7).

9 DISSEMINIATION POLICY

9.1 Dissemination policy

The University of Exeter owns the data arising from the study. On completion of the study, the data will be analysed and tabulated and a Final Study Report prepared. The full study report will be accessed through the study website as appropriate, and participating investigators will have rights to publish study data with full permission of the Chief Investigator. Results will be communicated through peer reviewed journal publications, conference presentations and communications on the sponsor's website.

Participants will be given a link to our study website in the PIS, which will be updated with all the latest trial documentation and advice, including contact details, Participant Information Sheets and sources of support for further information. When we have results, we will publish these in peer reviewed scientific journals and will communicate our findings through conference presentations and our institutional and study website.

9.2 Authorship eligibility guidelines and any intended use of professional writers

All investigators and coinvestigators will be eligible for authorship on final publications, and all authorship decisions will strictly adhere to ICJME guidance.

10 REFERENCES

1. Darzi A. Quality and the NHS next stage review. Lancet. 2008;371(9624):1563-4.

2. Vincent C, Amalberti R. Safer Healthcare: Strategies for the Real World. Cham (CH)2016.

3. Black N, Varaganum M, Hutchings A. Relationship between patient reported experience (PREMs) and patient reported outcomes (PROMs) in elective surgery. BMJ quality & safety. 2014;23(7):534-42.

4. Doyle C, Lennox L, Bell D. A systematic review of evidence on the links between patient experience and clinical safety and effectiveness. BMJ Open. 2013;3(1).

For peer review only - http://bmjopen.bmj.com/site/about/guidelines.xhtml

5. Department of Health. Report of the Mid Staffordshire NHS Foundation Trust Public Enquiry. London: Department of Health; 2013.

6. @BorisJohnson. It is hard to find the words to express my debt to the NHS for saving my life. The efforts of millions of people across this country to stay home are worth it. Together we will overcome this challenge, as we have overcome so many challenges in the past. #StayHomeSaveLives [twitter post]12/04/2020 10/05/2020.

7. Graham C, Kasbauer S, Cooper R, King J, Sizmur S, Jenkinson C, et al. An evaluation of a near real-time survey for improving patients' experiences of the relational aspects of care: a mixed-methods evaluation. Health Services and Delivery Research. Southampton (UK) 2018.

8. Murrells T, Robert G, Adams M, Morrow E, Maben J. Measuring relational aspects of hospital care in England with the 'Patient Evaluation of Emotional Care during Hospitalisation' (PEECH) survey questionnaire. BMJ Open. 2013;3(1).

9. Feo R, Conroy T, Jangland E, Muntlin Athlin A, Brovall M, Parr J, et al. Towards a standardised definition for fundamental care: A modified Delphi study. J Clin Nurs. 2018;27(11-12):2285-99.

10. Kitson A, Conroy T, Wengstrom Y, Profetto-McGrath J, Robertson-Malt S. Defining the fundamentals of care. International journal of nursing practice. 2010;16(4):423-34.

11. Canadian Nurses Association. CNA Brief to the National Advisory Committee on SARS and Public Health: Lessons learned and recommendations. Ottawa, Canada: Canadian Nurses Association; 2003.

12. Baumann A, Blythe, J., Underwood, J., Dzuiba, J.,. Capacity, casualization, and continuity: the impact of SARS Toronto, Canada: University of Toronto, Faculty of Nursing and McMaster University, School of Nursing; 2003.

13. Registered Nurses Association of Ontario. SARS Unmasked: Celebrating Resilience, Exposing Vulnerability. A Report on the Nursing Experience with SARS in Ontario. Toronto: Registered Nurses Association of Ontario; 2003.

14. Pentecost C, Frost J, Sugg HVR, Hilli A, Goodwin VA, Richards DA. Patients' and nurses' experiences of fundamental nursing care: A systematic review and qualitative synthesis. J Clin Nurs. 2020;29(11-12):1858-82.

15. Richards DA, Hilli A, Pentecost C, Goodwin VA, Frost J. Fundamental nursing care: A systematic review of the evidence on the effect of nursing care interventions for nutrition, elimination, mobility and hygiene. Journal of Clinical Nursing. 2018;27(11-12):2179-88.

16. Hayes RJ, Moulton LH. Cluster Randomised Trials 2nd Edition. Florida: Chapman and Hall/CRC; 2017.

17. Halcomb E, Davidson P,Hardaker L. Using the consensus development conference method in healthcare research. Nurse Res. 2008;16(1):56-71.

18. Nair R, Aggarwal R, Khanna D. Methods of formal consensus in classification/diagnostic criteria and guideline development. Semin Arthritis Rheum. 2011;41(2):95-105.

19. Waggoner J, Carline JD, Durning SJ. Is There a Consensus on Consensus Methodology? Descriptions and Recommendations for Future Consensus Research. Acad Med. 2016;91(5):663-8.

For peer review only

For peer review only - http://bmjopen.bmj.com/site/about/guidelines.xhtml

20. Wilde Larsson B, Larsson G. Development of a short form of the Quality from the Patient's Perspective (QPP) questionnaire. J Clin Nurs. 2002;11(5):681-7.

21. Graham C, Kasbauer S, Cooper R, King J, Sizmur S, Jenkinson C, et al. An evaluation of a near real-time survey for improving patients' experiences of the relational aspects of care: a mixed-methods evaluation. Health Services and Delivery Research. Southampton (UK)2018.

22. NHS Improvement. Guide to developing and implementing ward and unit accreditation programmes, IG 09/19. London: NHS Improvement; 2019.

23. Collin C, Wade DT, Davies S, Horne V. The Barthel ADL Index: a reliability study. Int Disabil Stud. 1988;10(2):61-3. doi: 10.3109/09638288809164103. PMID: 3403500.

24. Marshall J, On Behalf Of The Who Working Group On The Clinical Characteristics Of Covid-19 Infection,. A Core Outcome Measure Set for Studies of SARS-CoV2/COVID-19

25. Kroenke K, Spitzer RL, Williams JB. The Patient Health Questionnaire-2: validity of a two-item depression screener. Med Care. 2003;41(11):1284-92.

26. Kroenke K, Spitzer RL, Williams JB, Monahan PO, Lowe B. Anxiety disorders in primary care: prevalence, impairment, comorbidity, and detection. Ann Intern Med. 2007;146(5):317-25.

27. Epstein EG, Whitehead PB, Prompahakul C, Thacker LR, Hamric AB. Enhancing Understanding of Moral Distress: The Measure of Moral Distress for Health Care Professionals. AJOB Empir Bioeth. 2019;10(2):113-24.

28. Rafferty AM, Philippou J, Fitzpatrick JM, Pike G, Ball J. Development and testing of the 'Culture of Care Barometer' (CoCB) in healthcare organisations: a mixed methods study. BMJ Open. 2017;7(8):e016677.

29. Finch, T.L., Girling, M., May, C.R., Mair, F.S., Murray, E., Treweek, S., Steen, I.N., McColl, E.M., Dickinson, C., Rapley, T. (2015). NoMad: Implementation measure based on Normalization Process Theory. [Measurement instrument]. Retrieved from http://www.normalizationprocess.org

For peer review only

For peer review only - http://bmjopen.bmj.com/site/about/guidelines.xhtml

11. APPENDICIES

11.1 Appendix 1- Required documentation

All required documentation from local sites prior to initiation:

- CVs of the research team
- Certificate evidence of qualification
- Site Level Agreement

11.2 Appendix 2.1 – Schedule of Procedures (patient)

Procedures	Visits (insert visit numbers as appropriate)		
	**Screening**	**Baseline**	**Discharge**
Informed consent	x		
Demographics			x
Medical history			x
Patient Reported Outcome measures – QPP, RAC-Q, Barthel Index, PHQ-2			x
EQ5D			x
WHO Clinical Progression			x

Appendix 2 – Schedule of Procedures (nurse)

Procedures	Visits (insert visit numbers as appropriate)		
	**Screening**	**Baseline**	**Week 6**
Informed consent	x		
Demographics			x
Nurse Reported Outcome measures – MMC-HP, Culture of Care Barometer			x

11.3 Appendix 3 – Amendment History

Amendment No.	Protocol version no.	Date issued	Author(s) of changes	Details of changes made
1	2	07/12/2020	ExeCTU	Update and inclusion of new Trial Managers details

For peer review only

For peer review only - http://bmjopen.bmj.com/site/about/guidelines.xhtml

				Clarification and correction of various aspects of the trial:<br>Inclusion of student nurses, provision of materials for people who do not speak English, use of strategies such as social media and websites to raise awareness of study, use of other suitability qualified individuals to collect study data and consent rather than just Research Nurses, update of the randomisation system that will be used to randomise the sites, removal of reference of face to face interviews<br>Inclusion of the primary and secondary outcomes, and clarifying process evaluation measures<br>Access to medical records<br>Inclusion of safety reporting process, and clarification of what the TSC will receive<br>Update and clarification of how data will be stored<br>Removal of repetitive text<br>Clarification of materials provided to participant and how materials are provided<br>Updated text of what ethical approvals have been granted<br>References of measures added into reference list
3	3	20/01/2021	ExeCTU	Due to issues encountered with recruitment, a more flexible approach will be utilised to assess the process data<br>Flexibility regarding the number of sites and pairs needed has been built in<br>Information regarding the online training and FutureLearn has been added<br>Few minor typos corrected
4	4	03/02/2021	ExeCTU	The process for research nurses to collect data has been expanded to include contacting the patient after they have been discharged, and

For peer review only - http://bmjopen.bmj.com/site/about/guidelines.xhtml

				introducing the study over the telephone. PIS and consent forms could then be sent to the patients, if not collected in hospital, and once the site has received a returned consent form data will be collected and entered onto the ipad over the telephone<br><br>The availability of paper CRF's for the nurses has been made possible to expedite the data collected and enable the study to be more easily accessed by night staff. The data from the paper CRF's will be entered onto REDCap by either the research nurses, or other suitably trained or qualified members of the research team at site.
5	5	12/02/2021	ExeCTU	Experience enrolling wave one sites during October 2020-January 2021 has taught us that a sequential three wave rapid cycle cluster randomised controlled trial design with pair matched clusters is not feasible as sites face significant clinical and research pressures caused by the pandemic. As recommended by the independent Trial Steering Committee (TSC), we have therefore amended the study design to a simple cluster randomised controlled trial without pair matching or sequential waves, with a recruitment target of seven clusters per trial arm (14 sites) rather than nine (18 sites) which still gives us 90% power. We have also amended the analysis strategy to take into account these changes.

List details of all protocol amendments here whenever a new version of the protocol is produced.

For peer review only - http://bmjopen.bmj.com/site/about/guidelines.xhtml

Protocol amendments must be submitted to the Sponsor for approval prior to submission to the REC.

11.4 Appendix 4 Clinical Guideline

11.5 Appendix 5 Participant Information Sheet (patient)

See attachment in file.

11.6 Appendix 6 Participant Information Sheet (nurse)

See attachment in file.

11.7 Appendix 7 Consent form (patient)

See attachment in file.

11.8 Appendix 8 Consent form (nurse)

See attachment in file.

11.9 Appendix 9 Sponsor insurance

See attachment in file.

For peer review only

For peer review only - http://bmjopen.bmj.com/site/about/guidelines.xhtml

STANDARD PROTOCOL ITEMS: RECOMMENDATIONS FOR INTERVENTIONAL TRIALS

SPIRIT 2013 Checklist: Recommended items to address in a clinical trial protocol and related documents*

Section/item	Item No	Description
**Administrative information**		
Title	1	Descriptive title identifying the study design, population, interventions, and, if applicable, trial acronym P1
Trial registration	2a	Trial identifier and registry name. If not yet registered, name of intended registry P3
	2b	All items from the World Health Organization Trial Registration Data Set Yes
Protocol version	3	Date and version identifier P3
Funding	4	Sources and types of financial, material, and other support P3
Roles and responsibilities	5a	Names, affiliations, and roles of protocol contributors P14
	5b	Name and contact information for the trial sponsor P14
	5c	Role of study sponsor and funders, if any, in study design; collection, management, analysis, and interpretation of data; writing of the report; and the decision to submit the report for publication, including whether they will have ultimate authority over any of these activities P14
	5d	Composition, roles, and responsibilities of the coordinating centre, steering committee, endpoint adjudication committee, data management team, and other individuals or groups overseeing the trial, if applicable (see Item 21a for data monitoring committee) PP12-14
**Introduction**		
Background and rationale	6a	Description of research question and justification for undertaking the trial, including summary of relevant studies (published and unpublished) examining benefits and harms for each intervention P4-6
	6b	Explanation for choice of comparators P8
Objectives	7	Specific objectives or hypotheses P6

For peer review only

Trial design	8	Description of trial design including type of trial (eg, parallel group, crossover, factorial, single group), allocation ratio, and framework (eg, superiority, equivalence, noninferiority, exploratory) P6

Methods: Participants, interventions, and outcomes

Study setting	9	Description of study settings (eg, community clinic, academic hospital) and list of countries where data will be collected. Reference to where list of study sites can be obtained P6-7
Eligibility criteria	10	Inclusion and exclusion criteria for participants. If applicable, eligibility criteria for study centres and individuals who will perform the interventions (eg, surgeons, psychotherapists) P7
Interventions	11a	Interventions for each group with sufficient detail to allow replication, including how and when they will be administered P7-8
	11b	Criteria for discontinuing or modifying allocated interventions for a given trial participant (eg, drug dose change in response to harms, participant request, or improving/worsening disease) P12
	11c	Strategies to improve adherence to intervention protocols, and any procedures for monitoring adherence (eg, drug tablet return, laboratory tests) P12
	11d	Relevant concomitant care and interventions that are permitted or prohibited during the trial No restrictions (P8)
Outcomes	12	Primary, secondary, and other outcomes, including the specific measurement variable (eg, systolic blood pressure), analysis metric (eg, change from baseline, final value, time to event), method of aggregation (eg, median, proportion), and time point for each outcome. Explanation of the clinical relevance of chosen efficacy and harm outcomes is strongly recommended P8
Participant timeline	13	Time schedule of enrolment, interventions (including any run-ins and washouts), assessments, and visits for participants. A schematic diagram is highly recommended (see Figure) P8
Sample size	14	Estimated number of participants needed to achieve study objectives and how it was determined, including clinical and statistical assumptions supporting any sample size calculations P8-9
Recruitment	15	Strategies for achieving adequate participant enrolment to reach target sample size P9

Methods: Assignment of interventions (for controlled trials)

Allocation:

For peer review only - http://bmjopen.bmj.com/site/about/guidelines.xhtml

Sequence generation	16a	Method of generating the allocation sequence (eg, computer-generated random numbers), and list of any factors for stratification. To reduce predictability of a random sequence, details of any planned restriction (eg, blocking) should be provided in a separate document that is unavailable to those who enrol participants or assign interventions P9
Allocation concealment mechanism	16b	Mechanism of implementing the allocation sequence (eg, central telephone; sequentially numbered, opaque, sealed envelopes), describing any steps to conceal the sequence until interventions are assigned P9
Implementation	16c	Who will generate the allocation sequence, who will enrol participants, and who will assign participants to interventions P9
Blinding (masking)	17a	Who will be blinded after assignment to interventions (eg, trial participants, care providers, outcome assessors, data analysts), and how P9-10
	17b	If blinded, circumstances under which unblinding is permissible, and procedure for revealing a participant's allocated intervention during the trial P9-10
**Methods: Data collection, management, and analysis**		
Data collection methods	18a	Plans for assessment and collection of outcome, baseline, and other trial data, including any related processes to promote data quality (eg, duplicate measurements, training of assessors) and a description of study instruments (eg, questionnaires, laboratory tests) along with their reliability and validity, if known. Reference to where data collection forms can be found, if not in the protocol P9-10
	18b	Plans to promote participant retention and complete follow-up, including list of any outcome data to be collected for participants who discontinue or deviate from intervention protocols P9-11
Data management	19	Plans for data entry, coding, security, and storage, including any related processes to promote data quality (eg, double data entry; range checks for data values). Reference to where details of data management procedures can be found, if not in the protocol P10-11
Statistical methods	20a	Statistical methods for analysing primary and secondary outcomes. Reference to where other details of the statistical analysis plan can be found, if not in the protocol P10-11
	20b	Methods for any additional analyses (eg, subgroup and adjusted analyses) P10-11
	20c	Definition of analysis population relating to protocol non-adherence (eg, as randomised analysis), and any statistical methods to handle missing data (eg, multiple imputation) P11

For peer review only - http://bmjopen.bmj.com/site/about/guidelines.xhtml

Methods: Monitoring

Data monitoring	21a	Composition of data monitoring committee (DMC); summary of its role and reporting structure; statement of whether it is independent from the sponsor and competing interests; and reference to where further details about its charter can be found, if not in the protocol. Alternatively, an explanation of why a DMC is not needed P11
	21b	Description of any interim analyses and stopping guidelines, including who will have access to these interim results and make the final decision to terminate the trial P12
Harms	22	Plans for collecting, assessing, reporting, and managing solicited and spontaneously reported adverse events and other unintended effects of trial interventions or trial conduct P12
Auditing	23	Frequency and procedures for auditing trial conduct, if any, and whether the process will be independent from investigators and the sponsor P12

Ethics and dissemination

Research ethics approval	24	Plans for seeking research ethics committee/institutional review board (REC/IRB) approval P13
Protocol amendments	25	Plans for communicating important protocol modifications (eg, changes to eligibility criteria, outcomes, analyses) to relevant parties (eg, investigators, REC/IRBs, trial participants, trial registries, journals, regulators) n/a
Consent or assent	26a	Who will obtain informed consent or assent from potential trial participants or authorised surrogates, and how (see Item 32) P9
	26b	Additional consent provisions for collection and use of participant data and biological specimens in ancillary studies, if applicable n/a
Confidentiality	27	How personal information about potential and enrolled participants will be collected, shared, and maintained in order to protect confidentiality before, during, and after the trial P10, PP12-13
Declaration of interests	28	Financial and other competing interests for principal investigators for the overall trial and each study site P14
Access to data	29	Statement of who will have access to the final trial dataset, and disclosure of contractual agreements that limit such access for investigators P13-14
Ancillary and post-trial care	30	Provisions, if any, for ancillary and post-trial care, and for compensation to those who suffer harm from trial participation n/a

For peer review only - http://bmjopen.bmj.com/site/about/guidelines.xhtml

Dissemination policy	31a	Plans for investigators and sponsor to communicate trial results to participants, healthcare professionals, the public, and other relevant groups (eg, via publication, reporting in results databases, or other data sharing arrangements), including any publication restrictions PP13-14
	31b	Authorship eligibility guidelines and any intended use of professional writers P14
	31c	Plans, if any, for granting public access to the full protocol, participant-level dataset, and statistical code P13
**Appendices**		
Informed consent materials	32	Model consent form and other related documentation given to participants and authorised surrogates as appendix
Biological specimens	33	Plans for collection, laboratory evaluation, and storage of biological specimens for genetic or molecular analysis in the current trial and for future use in ancillary studies, if applicable n/a

*It is strongly recommended that this checklist be read in conjunction with the SPIRIT 2013 Explanation & Elaboration for important clarification on the items. Amendments to the protocol should be tracked and dated. The SPIRIT checklist is copyrighted by the SPIRIT Group under the Creative Commons "Attribution-NonCommercial-NoDerivs 3.0 Unported" license.

For peer review only

BMJ Open

COVID-NURSE: evaluation of a fundamental nursing care protocol compared to care as usual on experience of care for non-invasively ventilated patients in hospital with the SARS-CoV-2 virus: protocol for a cluster randomised controlled trial.

Journal:	*BMJ Open*
Manuscript ID	bmjopen-2020-046436.R2
Article Type:	Protocol
Date Submitted by the Author:	20-Apr-2021

Complete List of Authors:	Richards, David; University of Exeter, Institute for Health Research; Western Norway University of Applied Sciences Department of Health and Caring Sciences<br>Rose Sugg, Holly; University of Exeter, Institute for Health Research<br>Cockcroft, Emma; University of Exeter, Institute for Health Research<br>Cooper, Joanne; Nottingham University Hospitals NHS Trust, Corporate; University of Nottingham, School of Health Sciences<br>Cruickshank, Susanne; Royal Marsden NHS Foundation Trust<br>Doris, Faye; University of Exeter, Institute for Health Research<br>Hulme, Claire; University of Exeter, Health Economics Group<br>Logan, Phillipa; University of Nottingham, Community Health Sciences<br>Iles-Smith, Heather; University of Salford<br>Melendez-Torres, G.J; University of Exeter,<br>Rafferty, Anne Marie; King's College London, Florence Nightingale School of Nursing and Midwifery<br>Reed, Nigel; University of Exeter, Institute for Health Research<br>Russell, Anne-Marie; University of Exeter, School of Nursing<br>Shepherd, Maggie; University of Exeter, NIHR Clinical Research Facility<br>Singh, Sally; Leicester Royal Infirmary, Cardiac/Pulmonary Rehabilitation<br>Thompson Coon, Jo; University of Exeter, NIHR CLAHRC South West Peninsula<br>Tooze, Susannah; University of Exeter, Academy of Nursing<br>Wootton, Stephen; University of Southampton, Insitute of Human Nutrition<br>Abbott, Rebecca; University of Exeter, NIHR CLAHRC South West Peninsula<br>Bethel, Alison; University of Exeter, PenARC<br>Creanor, Siobhan; University of Exeter<br>Quinn, Lynne; University of Exeter, Clinical Trials Unit<br>Tripp, Harry; University of Exeter, Clinical Trials Unit<br>Warren, Fiona; University of Exeter, Institute of Health Research<br>Whear, Rebecca; University of Exeter<br>Bollen, Jessica; University of Exeter, Clinical Trials Unit<br>Hunt, Harriet; University of Exeter Medical School, Exeter Medical School<br>Kent, Merryn; University of Exeter, Institute for Health Research<br>Morgan, Leila; Royal Devon and Exeter NHS Foundation Trust<br>Morley, Naomi; University of Exeter,

	Romanczuk, Lidia; University of Exeter, Institute for Health Research
**Primary Subject Heading**:	Nursing
Secondary Subject Heading:	Health services research, Infectious diseases, Respiratory medicine
Keywords:	COVID-19, INFECTIOUS DISEASES, Protocols & guidelines < HEALTH SERVICES ADMINISTRATION & MANAGEMENT, Clinical trials < THERAPEUTICS

SCHOLARONE™ Manuscripts

I, the Submitting Author has the right to grant and does grant on behalf of all authors of the Work (as defined in the below author licence), an exclusive licence and/or a non-exclusive licence for contributions from authors who are: i) UK Crown employees; ii) where BMJ has agreed a CC-BY licence shall apply, and/or iii) in accordance with the terms applicable for US Federal Government officers or employees acting as part of their official duties; on a worldwide, perpetual, irrevocable, royalty-free basis to BMJ Publishing Group Ltd ("BMJ") its licensees and where the relevant Journal is co-owned by BMJ to the co-owners of the Journal, to publish the Work in this journal and any other BMJ products and to exploit all rights, as set out in our licence.

The Submitting Author accepts and understands that any supply made under these terms is made by BMJ to the Submitting Author unless you are acting as an employee on behalf of your employer or a postgraduate student of an affiliated institution which is paying any applicable article publishing charge ("APC") for Open Access articles. Where the Submitting Author wishes to make the Work available on an Open Access basis (and intends to pay the relevant APC), the terms of reuse of such Open Access shall be governed by a Creative Commons licence – details of these licences and which Creative Commons licence will apply to this Work are set out in our licence referred to above.

Other than as permitted in any relevant BMJ Author's Self Archiving Policies, I confirm this Work has not been accepted for publication elsewhere, is not being considered for publication elsewhere and does not duplicate material already published. I confirm all authors consent to publication of this Work and authorise the granting of this licence.

For peer review only

COVID-NURSE: evaluation of a fundamental nursing care protocol compared to care as usual on experience of care for non-invasively ventilated patients in hospital with the SARS-CoV-2 virus: protocol for a cluster randomised controlled trial

David A. Richards, Holly V.R. Sugg, Emma Cockcroft, Joanne Cooper, Susanne Cruickshank, Faye Doris, Claire Hulme, Phillipa Logan, Heather Iles-Smith, G.J. Melendez-Torres, Anne Marie Rafferty, Nigel Reed, Anne-Marie Russell, Maggie Shepherd, Sally J. Singh, Jo Thompson Coon, Susannah Tooze, Stephen Wootton, Rebecca Abbott, Alison Bethel, Siobhan Creanor, Lynne Quinn, Harry Tripp, Fiona C. Warren, Rebecca Whear, Jessica Bollen, Harriet A. Hunt, Merryn Kent, Leila Morgan, Naomi Morley, Lidia Romanczuk

Corresponding author

David A. Richards
University of Exeter
St Luke's Campus
Heavitree Road
Exeter EX1 2LU
United Kingdom

d.a.richards@exeter.ac.uk

David A. Richards, University of Exeter, Exeter, UK; and Western Norway University of Applied Sciences, Bergen, Norway

Holly V. R. Sugg, University of Exeter, Exeter. UK

Emma Cockcroft, University of Exeter, Exeter, UK

Joanne Cooper, Nottingham University Hospitals NHS Trust, Nottingham, UK

Susanne Cruickshank, Royal Marsden NHS Foundation Trust, London, UK

Faye Doris, University of Exeter, Exeter, UK

Claire Hulme, University of Exeter, Exeter, UK

Phillipa Logan, University of Nottingham, Nottingham, UK

Heather Iles-Smith, University of Salford, Salford, UK

G.J. Melendez-Torres, University of Exeter, Exeter, UK

Anne Marie Rafferty, King's College, London, UK

Nigel Reed, University of Exeter, Exeter, UK

Anne-Marie Russell, University of Exeter, Exeter, UK

Maggie Shepherd, University of Exeter, Exeter, UK

For peer review only

For peer review only - http://bmjopen.bmj.com/site/about/guidelines.xhtml

Sally J. Singh, University Hospitals of Leicester NHS Trust, Leicester, UK

Jo Thompson Coon, University of Exeter, Exeter, UK

Susannah Tooze, University of Exeter, Exeter, UK

Stephen Wotton, University of Southampton, Southampton. UK

Rebecca Abbott, University of Exeter, Exeter, UK

Alison Bethel, University of Exeter, Exeter, UK

Siobhan Creanor, University of Exeter, Exeter, UK

Lynne Quinn, University of Exeter, Exeter, UK

Harry Tripp, University of Exeter, Exeter, UK

Fiona C. Warren, University of Exeter, Exeter, UK

Rebecca Whear, University of Exeter, Exeter, UK

Jessica Bollen, University of Exeter, Exeter, UK

Harriet A. Hunt, University of Exeter, Exeter, UK

Merryn Kent, University of Exeter, Exeter, UK

Leila Morgan, Royal Devon and Exeter NHS Foundation Trust, Exeter, UK

Naomi Morley, University of Exeter, Exeter, UK

Lidia Romanczuk, University of Exeter, Exeter, UK

Keywords: Nursing, Covid-19, Infectious Diseases, Protocols and Guidelines (Health Services Administration & Management), Clinical Trials (Therapeutics)

Word count 4670

For peer review only

COVID-NURSE: evaluation of a fundamental nursing care protocol compared to care as usual on experience of care for non-invasively ventilated patients in hospital with the SARS-CoV-2 virus: protocol for a cluster randomised controlled trial

Protocol Version 5.0 05-02-2021

Funder: NIHR/UKRI/MRC

Study Grant Ref: MR/V02776X/1

Trial Registration: ISRCTN13177364

Ethics approval: IRAS ID 288479; REC reference: 20/NE/0253

Abstract:

Introduction; Patient experience of nursing care is correlated with safety, clinical effectiveness, care quality, treatment outcomes and service use. Effective nursing care includes actions to develop nurse-patient relationships and deliver physical and psychosocial care to patients. The high risk of transmission of the SARS-CoV-2 virus compromises nursing care. No evidence-based nursing guidelines exist for patients infected with SARS-CoV-2, leading to potential variations in patient experience, outcomes, quality and costs.

Methods and analysis; we aim to recruit 840 in-patient participants treated for infection with the SARS-CoV-2 virus from 14 UK hospitals, to a cluster randomised controlled trial, with embedded process and economic evaluations, of care as usual plus a fundamental nursing care protocol addressing specific areas of physical, relational and psychosocial nursing care where potential variation may occur, compared to care as usual. Our co-primary outcomes are patient-reported experience (Quality from the Patients' Perspective; Relational Aspects of Care Questionnaire); secondary outcomes include care quality (pressure injuries, falls, medication errors); functional ability (Barthell Index); treatment outcomes (WHO Clinical Progression Scale); depression (PHQ-2), anxiety (GAD-2), health utility (EQ5D), and nurse-reported outcomes (Measure of Moral Distress for Health Care Professionals). For our primary analysis we will use a standard generalised linear mixed-effect model adjusting for ethnicity of the patient sample and research intensity at cluster level. We will also undertake a planned sub-group analysis to compare the impact of patient-level ethnicity on our primary and secondary outcomes and will undertake process and economic evaluations.

For peer review only - http://bmjopen.bmj.com/site/about/guidelines.xhtml

Ethics and dissemination; research governance and ethical approvals are from the UK National Health Service Health Research Authority Research Ethics Service. Dissemination will be open access through peer reviewed scientific journals, study website, press and online media, including free online training materials on the Open University's FutureLearn web platform.

Strengths and limitations of this study

- This study is the first randomised controlled trial of a fundamental care clinical nursing protocol for patients infected with the SARS-CoV-2 virus
- The intervention relates specifically to patients with the SARS-CoV-2 virus admitted to inpatient wards who are not invasively ventilated
- The intervention programme theory will enable generalisation of our findings to other environments such as care homes, patients with other conditions requiring isolation and global health systems
- The trial co-primary outcomes require patient participants to have capacity to consent and report their experience of care, and therefore we will be unable to collect and report data from patients who lack this capacity
- In common with other cluster randomised controlled trials of behaviour change interventions we are unable to blind nurses, participants or data collectors to trial arm allocation

Background

Patient experience of nursing care is correlated with safety, clinical effectiveness, care quality, treatment outcomes including mortality and overall service use.(1-7) Nursing care is a key determinant of patient experience(8, 9) and satisfaction.(10) Effective nursing care includes the establishment of compassionate nurse-patient relationships known as 'relational nursing care' in order to facilitate the physical and psychosocial care work undertaken by nurses to meet the fundamental care needs of patients such as nutrition, hydration, skin integrity, personal care, mobility, hygiene, breathing, elimination and mental wellbeing.(11, 12)

Measuring patient experience is analogous to collecting a patient-reported outcome measure (PROM)(13) in that both are "*a measurement based on a report that comes directly from the patient (i.e., study subject*)".(14) However, whereas a PROM captures "*the status of a patient's health condition without amendment or interpretation of the patient's response by a clinician or anyone else*",(14) a patient-reported *experience* measure (PREM) is a measure of a patient's perception of their personal experience of the healthcare they have received.(15) Two PREMs for evaluating

For peer review only

patients' experiences of nursing have been developed and tested; the Relational Aspects of Care Questionnaire – RACQ),(8, 16) and the Quality from the Patient's Perspective – QPP.(17, 18)

The combination of COVID-19 symptoms and high risk of transmission of the SARS-CoV-2 virus poses unique challenges for physical, psychosocial and relational nursing care. Previously, the 2003 SARS outbreak demonstrated that nurses' ability to provide compassionate fundamental care was compromised: "*The establishment/maintenance of therapeutic nurse-client relationship required additional time given the barriers of mask, gloves and gowns*".p4(19) "*Restrictions on visitors were difficult for staff because family members are usually involved in the social, psychological and, to some extent, physical care of patients*".p6(20) "*Interaction time decreased, and patients began to feel more abandoned*".p28(21)

During the development of our trial protocol described below, we found no international evidence-based guidelines for nursing hospitalised patients with the SARS-CoV-2 virus, the majority of whom are not invasively mechanically ventilated. This leads to the potential for variations in patient experience, treatment outcomes, care quality and costs, as reported by nurses who responded to our survey, also described below. Although individual nurses, teams and organisations reported rapidly adapting their procedures to nurse these patients, compared to usual nursing care, we as yet do not know how to optimise nursing care for the specific challenges of nursing COVID-19 patients, including mitigating the impact of wearing personal protective equipment, nor how effective these adaptations are in terms of these important outcomes.

Between July and October 2020, we developed a clinical nursing protocol. We undertook a rapid review(22) of published literature to identify the evidence for the effectiveness of, and barriers to, fundamental nursing care procedures in patients with SARS-CoV-2 virus or other conditions requiring isolation, in terms of overall patient experience, care quality, functional ability and treatment outcomes – PROSPERO registration number: CRD4202020091.(23) We also ran a UK-wide survey of nurses and non-registered care staff working with hospitalised patients with the SARS-CoV-2 virus who were not invasively ventilated to identify respondents' views on 'missed care',(24, 25) barriers to care, and innovation strategies respondents had adopted to meet the fundamental care needs of such patient. We obtained research governance approval from the Health Research Authority (IRAS project ID: 287288, protocol number 1920/31) and ethical approval from the University of Exeter research ethics committee (no: 20/07/256) for this survey. We then convened three consensus development panels(26-28) of nurses, and patients with experience of hospitalisation with the SARS-CoV-2 virus, to agree the content and design of the clinical nursing protocol. We will report the results of our systematic review and survey elsewhere.

For peer review only - http://bmjopen.bmj.com/site/about/guidelines.xhtml

In the COVID-NURSE trial, our objectives are:

a) to compare care as usual plus an evidence-based nursing protocol for patients with the SARS-CoV-2 virus against care as usual, in terms of patients' reported experience of transactional and relational nursing care, care quality, treatment outcomes and costs

b) to compare the effects of using the clinical protocol on nurses' moral distress

Methods: Participants, Interventions, and Outcomes

Design: we will undertake a cluster randomised controlled superiority trial(29, 30) with embedded iterative process(31) and economic evaluations(32) between 02/11/2020 and 18/10/2021). Our initial funded, approved and registered design was a rapid-cycle cluster randomised controlled trial(33) with three review 'waves'. Like adaptive trial designs, rapid cycle trials embed *a priori* opportunities into trial designs to enable review and minor adaptions to interventions between waves. We also planned to pair-match 18 trial sites according to two variables with a potential impact on outcomes: 1) ethnicity, where being of Black and Asian ethnicity (BAME) increases the risk of SARS-COV-2 infection compared to White individuals and where people of Asian ethnicity may be at higher risk of ITU admission and death(34) and 2) research intensity which is associated with improved hospital outcomes.(35) We planned to classify ethnicity according to the latest census data for the local authority in which a hospital is located (Low (<68%) vs Medium (68% to 74%) vs High (>74%) non-BAME categories based around an overall mean of 71% non-BAME admissions. We planned to classify research intensity according to two categories: research intensive vs not research intensive, where research intensive is defined as ≥0.1 unique recruiting studies in the 2019-2020 Q4 open data from NIHR divided by number of inpatient elective admissions in Jan-Mar 2020, and non-research intensive is <0.1.

Since submitting this protocol for peer review by this journal, experience enrolling wave one sites during October 2020-January 2021 taught us that our ideal situation is subject to considerable stress as sites adapt to a rapidly changing situation on the ground, facing significant clinical and research pressures. Although we successfully recruited three pairs (six sites) of sites matched as above for our planned first wave during October-December 2020, operational difficulties caused by a) the trial not being prioritised for NHS research infrastructure resources and b) the impact of the second wave of the SARS-COV-2 virus on nursing sickness levels, led to very significant delays to site opening. Our six wave one sites were unable to start data collection concurrently, delaying our planned wave one review. Further, we recruited several other sites for waves two and three for which we could not find a match on the variables listed above. These issues threatened to prevent us recruiting to time and target within the funded period (July 2020-April 2021). Therefore, in order for the trial to recruit

For peer review only - http://bmjopen.bmj.com/site/about/guidelines.xhtml

sufficient sites and participants and for us to retain our ability to review and make minor amendments to the intervention in response to process data on fidelity and acceptability, we amended the design to a simple cluster randomised trial, with an embedded iterative process evaluation. As a consequence, as part of the statistical analysis plan, we will also adjust for ethnicity of the patient sample at cluster level and, where patient outcomes are tested, at patient level rather than pre-randomisation. Finally, we revised our sample size calculations, see below and obtained additional funding to continue the trial for a further six months from the previous planned end date of 18th April 2021. We began data collection on 18th January 2021 and plan to continue until the end of August 2021.

Setting: we will recruit 14 UK district-general and teaching hospital NHS Trusts (clusters). Participants will be recruited from one or more wards at each site where patients are being treated for infection with the SARS-COV-2 virus.

Participant eligibility criteria: patients who are not invasively ventilated, aged ≥18 years, currently hospitalised and being treated for infection with the SARS-CoV-2 virus, or recently discharged after such treatment and who have received nursing care for a minimum of 72 hours during their admission; registered nurses and nursing care workers working under the supervision of registered nurses caring for patients hospitalised and treated for infection with the SARS-CoV-2 virus. Participants must be able to give informed consent (appendices 1 and 2). We will provide translation facilities for participants unable to understand and speak English. We will recruit participants specifically from wards allocated to the care of patients admitted for treatment of COVID-19 symptoms.

Interventions:

Experimental Intervention: care as usual plus the clinical protocol developed as described above.

In order to preserve intervention blinding across trial control sites, we have not included the full clinical guideline here. To do so would potentially unblind nurses working in control cluster sites. Although it is an ethical principle to make a full intervention protocol available for peer and reader scrutiny, there is a competing and equal ethical principle to preserve control cluster integrity and prevent contamination of control sites. This ensures that all participants in a trial contribute their data to a study that is neither compromised nor subject to significant potential biases. Trials with compromised blinding are often rejected in systematic reviews or lead to uncertain conclusions. Below is a summary, therefore. However, we commit to making all written and online materials available to the scientific and clinical community free of charge at the conclusion of the trial, to include a free to access Massive Online Open Course supporting the intervention. In the interim, we

For peer review only - http://bmjopen.bmj.com/site/about/guidelines.xhtml

will refuse no reasonable request to view our guideline, having assured ourselves that a request will not compromise intervention blinding.

The protocol consists of four elements adapted from the methods used successfully in a previous cluster randomised controlled trial to significantly increase handwashing by nurses and care staff(36): a guideline, trigger reminder posters, staff education programme and leadership from ward managers and senior nurses.

The clinical guideline consists of 26 potential strategies that can be used by nurses to address barriers to physical, relational and psychosocial nursing care identified in our survey and systematic review. In our survey (37), eight barriers were ranked within the top five in at least one component of the three physical, relational and psychosocial care areas. These were: wearing Personal Protective Equipment (PPE), the severity of patients' conditions, inability to take items in and out of isolation rooms without donning and doffing PPE, lack of time to spend with patients, lack of presence from specialised services e.g. physiotherapists, lack of knowledge about COVID-19, insufficient stock, and reluctance to spend time with patients for fear of catching the SARS-CoV-2 virus.

From this data, we grouped the guideline strategies thematically into actions which address: a) communication with patients, with patients' significant others, between patients and their significant others, between nurses, and between nurses and other members of the care team; b) the organisation of fundamental nursing care activities; c) addressing the values of patients and their significant others; d) delivering specific fundamental nursing care interventions; e) identifying and responding to the mental health and wellbeing needs of patients' and their significant others.

The trigger posters contain key information on these same strategies to remind nurses to utilise them. The online educational resource includes video testimony from patients, carers, nurses and scientists about these same care activities and is hosted on the Open University's 'FutureLearn' platform,(38). Finally, the guideline includes advice to managers on organising care teams, educating and supporting members of their nursing teams, information on this also delivered via the 'FutureLearn' platform.(38)

We will monitor clinical protocol fidelity and acceptability as part of our process evaluation and modify our guideline, educational and leadership strategies iteratively, making minor adjustments to these elements according to findings from our process evaluation quantitative and qualitative data. We will embed any changes to the protocol in the subsequent experimental intervention sites.

Control: care as usual only. We will record staffing staff skill mix details alongside a description of clinical nursing procedures and organisation in place in the usual care clusters.

For peer review only - http://bmjopen.bmj.com/site/about/guidelines.xhtml

Outcomes: we will collect participant-level outcome data. Our co-primary outcomes will be patient-reported experience measures addressing transactional nursing care using the QPP(17, 18) and relational nursing care using the RACQ.(8, 16) Our secondary outcomes will include: measures of quality of care (pressure injuries, falls, medication errors) collected by the United Kingdom National Health Service Ward to Board dashboard(39); functional ability by the Barthel Index;(40, 41) treatment outcomes by the WHO Clinical Progression Scale;(42) depression by the PHQ-2,(43), anxiety by the GAD-2,(44), health utility by the EQ5D,(45) and nurse outcomes by the Measure of Moral Distress for Health Care Professionals.(46, 47)

Participant timeline: we will undertake the trial during 50 weeks of intervention and rolling data collection, with data collected from patient participants admitted for treatment of SARS-CoV-2 infection and staff caring for them.

Target sample size:

Using published formulae by Hayes and Moulton (16) we calculated our patient participant sample size based on an estimate of the minimum clinically important difference for the QPP of 0.2 and the typical within-unit standard deviation (0.6), supplied by the measure developers, and an ICC of 0.02. We have examined a number of potential scenarios, taking into account different cluster sizes and different between-cluster standard deviation values informed by between-country differences on measures, estimated to provide 90% and 80% power. Based on conversations on feasibility with NHS clinical leaders, with a cluster size of 60 the trial would generate over 80% power with 6 clusters per arm and 90% power with 7 clusters in each arm. However, because power calculations in cluster trials with a small number of clusters can be especially sensitive to the approximation used for degrees of freedom, we used a range of alternative approaches (no small sample adjustment, Satterthwaite approximation, and Kenward-Rogers approximation; cluster-level linear regression) to explore the robustness of the sample size calculations. While these tended to estimate reduced power at each number of clusters compared to a formula-based power calculation, an estimate of 14 clusters (7 per arm) generated power of greater than 80% in every case. This number of clusters is fairly robust to number of patients per cluster. Even assuming under-recruitment leading to 50 patients per site, power is maintained at greater than 80%. Therefore, we will aim to recruit 840 patient participants. Although we have not powered the sample size on nurse participant outcomes, in consultation with clinical colleagues in our investigator and advisory groups, we anticipate that around 50 registered nurses and healthcare support workers will be involved in the direct care of these patient participants leading to a recruitment target of 700 registered nurse and nursing care worker participants, including student nurses.

For peer review only - http://bmjopen.bmj.com/site/about/guidelines.xhtml

Recruitment: we will recruit cluster sites via the NHS chief nurse and clinical research networks (CRN). We will monitor available data from Public Health England on case and hospitalisation rates by NHS Trust in order to identify recruitment sites where there are sufficient potential participants. We will use these data to prepare sites for involvement. Clinical research nurses will recruit patient participants from admitted patients according to the eligibility criteria above.

Allocation: we will randomly allocate sites to the intervention and control groups by an unblinded statistician who has no role in site recruitment or data analysis to ensure allocation concealment; only the unblinded statistician will have access to the allocation list. Randomisation will use a static blocked list through the use of an externally administered, password-protected randomisation website independently developed and maintained by the UKCRC-registered University of Exeter Clinical Trials Unit.

Data collection: we will collect outcome data from patient participants after they have received nursing care for a minimum of 72 hours, either during their admission or a maximum of two weeks post discharge. All outcome measures will be applied to all participants equally and collected by clinical research nurses and other Good Clinical Practice(48) trained research staff, who will also obtain consent. It will not be possible to blind research nurses, clinicians or patient participants to allocation, although control group cluster sites will be blind to the intervention as described earlier. Statistical analysts will be blind to group allocation when receiving data to analyse. We will collect outcome data either face to face, by video/audio technology or by post following discharge according to the infection control procedures in place and participant preference. We will collect outcome data from nurses either face to face in their hospital, or over the phone, according to their preference. Site level *Ward to Board* data will be collected from routine hospital statistics.

Data management: participants will be identified by a unique study ID**.** Personal identifiable data including date of birth and NHS number, may be collected but will be stored separately to research data and will be destroyed as per applicable regulations when the project is concluded. Data will be managed by the UKCRC registered Exeter Clinical Trials Unit (ExeCTU) following GDPR and data protection guidelines and all relevant Clinical Trial Regulations. All data will be anonymised prior to publication. Data will be collected and stored electronically in accordance with the Data Protection Act 2018 and ICH GCP E6 R2. ExeCTU will use Redcap Cloud Electronic Data Capture System to collect all case report data. This system is validated to ISO27001 standards, backed up and maintained in Europe. This system is fully compliant to GDPR regulations and managed by ExeCTU. Any additional study data will be stored and backed up on the secure ExeCTU servers, maintained by ExeCTU. Data will be cleaned and validated appropriately and a full Data Protection Impact Assessment (DPIA) will

For peer review only

be undertaken along with the development of a comprehensive Data Management Plan (DMP) before the first participant is recruited.

Where data are disseminated (e.g. via report, presentation or publication), they will be anonymised. We will align all confidentiality and data handling with the Caldicott Principles.(49) Anonymised data will be stored indefinitely on a research data storage system provided by the University of Exeter called Open access Research Exeter (ORE) for archiving (http://www.exeter.ac.uk/research/openresearch/policies/ore/).

Statistical data analysis: we will analyse numerical data in Stata v.16.(50) Our primary analysis for patient and nurse outcomes will use a standard generalised linear mixed-effect model with appropriate small-sample adjustment to account for the small number of clusters, and adjusting for ethnicity of the patient sample at cluster level and, where patient outcomes are tested, at patient level as well. Sensitivity analyses for patient and nurse outcomes will use variance-weighted cluster-level summaries with adjustment for ethnicity based on 2011 census and hospital postcode and for research intensity. Analysis of hospital outcomes will use rate ratios. Secondly, and focusing on patient-level outcomes, we will estimate intervention effectiveness in an exploratory analysis. This analysis will use generalised linear mixed-effects models to model time trends. Analysis will include time from implementation, reflecting growing adaptation and expertise, as well as calendar time, reflecting the course of the COVID-19 epidemic. We will also undertake a planned sub-group analysis to compare the impact of patient-level ethnicity on our primary and secondary outcomes.

Missing data: data are collected from patients and nurses at one time point. As a result, missingness is likely to be at item-level within scales and at scale level. Where >50% of items in a scale are completed (including 'not applicable' or 'don't know' responses), a scale score will be generated by either taking the average of remaining items (all QPP subscales, RAC-Q-14, MMD-HP) or by rescaling sum scores to the full range (Barthel Index). This is an appropriate strategy when factor loadings are homogeneous and reliability for scales is good.(51) Observations from single-item or two-item scales (WHO Clinical Progression Scale, PHQ-2, GAD-2) will be dropped from the analysis.

Process evaluation: we will evaluate the impact of nurses' and nursing care workers' fidelity to the clinical protocol, intervention mechanisms and context of care delivery on outcomes using: a bespoke instrument for capturing fidelity to reflect the protocol components; the Culture of Care Barometer Questionnaire;(52) the NoMAD instrument for measuring implementation readiness and acceptability;(53, 54) contextual data on patient acuity, staffing ratios, nurses/care staff education levels, seniority and year of qualification, and other contextual variables. We will conduct interim analyses of process evaluation data on fidelity to the protocol, and protocol utilisation to evaluate

For peer review only - http://bmjopen.bmj.com/site/about/guidelines.xhtml

intervention component fidelity and implementation. We will subsequently amend the clinical protocol based on this data before embedding the new protocol in intervention sites.

We will conduct semi-structured qualitative interviews with a purposive sample of nurses and care staff, using a topic guide derived from the content of the clinical protocol and our analysis of quantitative data, to explore their views on the mechanisms, impact and acceptability of the clinical protocol. This method will enable us to investigate the meaning of participants' responses, both exploring views on our predefined topics of interest and eliciting more detail on any emerging themes.(55) Interviews will be conducted using telephone or online audio-conference methods, unless face to face interviews are risk assessed as being safe and are preferred by participants. With participants' consent (appendices 1 and 2), we will audio-record interviews and transcribe interviews verbatim. We will use NVivo10(56) to organise the data and analyse data using Framework analysis to allow for the combination of inductive and deductive approaches in our development of themes.(57) We will use joint displays(58) to integrate the qualitative data with the quantitative outcome data in a mixed methods analysis(59) to explore and explain variation in the outcome data. We will use this data to refine an intervention programme theory(60, 61) incorporating context, mechanisms and fidelity.(31)

Economic evaluation: we will conduct within trial cost effectiveness analysis by adopting the NHS perspective with the main outcome measure the Quality-adjusted life year (QALY) derived from the EQ-5D-5L.(32, 45) We will apply the NICE threshold of £20,000 per QALY. Cost data will include staff training, length of stay, including ICU costs and COVID-19-specific interventions from baseline to discharge using a bespoke hospital care use inventory. We will apply unit costs derived from national sources including Personal Social Services Research Unit (62) and NHS Reference Costs.(63) Non-parametric bootstrapping will be used to quantify uncertainty. We will present outputs as ICERs, cost effectiveness acceptability curves and expected net benefit.

Monitoring: day to day management of the trial and study will be by the chief-investigator, other co-investigators and a research manager. We will set up an independent combined Trial Steering and Data Monitoring Committee (TSC/DMC)(48) and a Patient and Public Involvement group.(64) We will undertake a risk assessment of the trial and produce a monitoring plan that will be commensurate with the risk identified to particularly ensure that we have input from people of colour, including from Black, Asian and other minority ethnic groups, who are at greater risk from infection with the SARS-CoV-2 virus.

Safety reports of serious adverse events (SAE)

For peer review only - http://bmjopen.bmj.com/site/about/guidelines.xhtml

Given this trial is not a clinical trial of an investigational medicinal product (CTIMP) the recording and reporting of non-serious adverse events (AEs) is not required(65) since, although likely to be common in the target population, they are unlikely to be related to either the intervention or trial participation. Likewise, by nature of the patient participants' clinical condition, we will expect some serious adverse events (SAEs), specifically death or prolongation of hospital stay, (65) but that these will be both expected and unlikely to be related to intervention or trial procedures. As all non-CTIMP research is required to adhere to the principles of Good Clinical Practice(48, 66) it was nonetheless agreed with the trial sponsor, research ethics committee (Health Research Authority, North East - Newcastle & North Tyneside 2 Research Ethics Committee**,** reference: 20/NE/0253) and independent TSC/DMC to record and review each SAE by the CI on a case-by-case basis to determine if it is related to the trial intervention or procedures. This process is overseen by the TSC/DMC.

Patient and Public Involvement: The proposed study has been informed by ongoing collaborations between patients, clinicians and researchers, including in a previous NIHR-funded project (ESSENCE) on fundamental nursing care.(67) A version of the lay summary was reviewed by six members of the 'Peninsula Patient Involvement Group', giving feedback on the broad idea of the research as well as specific points on the clarity of the summary. A patient co-applicant has been involved with the development of the proposal. We will ensure there is a patient voice in all discussions and decisions, and will ensure the integration of broader PPI activity in these decisions. We have worked with a wider group of patients with experience of hospitalisation for COVID-19 symptoms to inform the co-production of the nursing protocol, development of all patient facing materials, finalising the nursing protocol for rapid-cycle testing, adaptions to the nursing protocol after each cycle of testing, dissemination of findings, including the production of an online training module for nurses on the FutureLearn platform. The involvement of patients will be informed by what is possible within the ongoing pandemic, and to suit the situation of interested patients. Involvement will be flexible and responsive to their needs. We will adopt high standards of equality, diversity and inclusivity(68) throughout.

Ethical issues: we will conduct this trial in such a way as to protect the human rights and dignity of the participants, as reflected in the Helsinki Declaration.(69) We have obtained governance and ethical approval from the Health Research Authority National Research Ethics Service (IRAS project ID 288479; REC reference: 20/NE/0253). Participants will not receive any financial inducement to participate. We will invite eligible patients and nurses to participate in data collection and will only collect data from participants who individually consent. Participants may withdraw from the study at any time without prejudicing their treatment, care or employment. We will conform to Good Clinical Practice Guidelines, data protection and freedom of information acts. We registered the trial with

For peer review only - http://bmjopen.bmj.com/site/about/guidelines.xhtml

the ISRCTN registry prospectively on 9th September 2020, before our intended data collection start date and have registered protocol amendments when required.

Outputs and dissemination: we will disseminate our results through publication in peer reviewed scientific journals, our study website and other press and online media. Manuscript authors will be those considered to have made a substantive intellectual contribution to the study. The investigators and relevant authorities will have access to the trial dataset. Furthermore, we will store anonymised research data and outputs in the University of Exeter's Open Research Exeter repository (https://ore.exeter.ac.uk/repository/) in order to facilitate open access to, and the impact of, our research.

The main output from this study will be evidence on whether our COVID-19-specific clinical nursing care protocol delivers additional benefits in overall patient experience, care quality, functional ability and treatment outcomes, with cost-effectiveness estimates, for hospitalised patients with the SARS-CoV-2 virus not invasively ventilated, compared to care as usual in hospital wards. We will have a programme theory allowing us to potentially generalise any such benefits to environments like care homes, patients with other conditions requiring isolation and to global health systems. If effective, our guidelines, education materials and strategies will be made accessible via Health Education England, the Open University's FutureLearn(38) web platform, and through experienced NHS sites acting as training and dissemination hubs for other health Trusts, environments such as care homes, and global health systems.

Discussion

The strengths of this study include that it is the first randomised controlled trial of a fundamental care clinical nursing protocol for patients infected with the SARS-CoV-2 virus and the intervention relates specifically to these patients admitted to inpatient wards who are not invasively ventilated. Our intervention programme theory will enable generalisation of our findings to other environments such as care homes, patients with other conditions requiring isolation and global health systems. In terms of limitations, the trial co-primary outcomes require patient participants to have capacity to consent and report their experience of care, and therefore we will be unable to collect and report data from patients who lack this capacity. In common with other cluster randomised controlled trials of behaviour change interventions we are unable to blind nurses, participants or data collectors to trial arm allocation.

Our research aligns with the WHO COVID-19 R&D Roadmap priorities and research gaps,(70) viz: 'to determine optimal clinical practice strategies to improve the processes of care'. It includes 'rapid approaches to capture healthcare worker views (surveys, interviews)' and a rapid ethnograph[y] in

For peer review only - http://bmjopen.bmj.com/site/about/guidelines.xhtml

healthcare'.(70) It will, therefore, provide guidance to nurses attempting to overcome significant barriers to providing fundamental transactional and relational care to patients; specifically those with the SARS-CoV-2 virus not invasively ventilated but with a generalisable programme theory suitable for adaptation to other care environments, other pandemics and other nations globally.

Authors' contributions: DR wrote the protocol with input from co-investigators HVRS, EC, JC, SC, CH, PL, HI-S, GJM-T, NR, AMRa, AMRu, MS, SJS, J, T-C, ST and SW. All other authors (RA, AB, SC, FD, LQ, HT, FW, RW, JB, HAH, KM, LM, NM and LR) reviewed the manuscript and made editorial suggestions. FD and NR are independent patient and public Involvement members of the team, GJM-T is the senior trial statistician and CH the trial economist. The trial team include nurses (DR, JC, SC, HI-S, GJM-T, AMRa, AMRu, MS, ST, LM, NM, LR), a physiotherapist (SJS), an occupational therapist (PL), a dietician (SW), medical statisticians (GJM-T, SC, FCW), an information specialist (AB), systematic reviewers (JT-C, RA, RW), a health economist (CH), health scientists and clinical trialists (DAR, HVRS, LQ, HT, JB, HAH), patient and public involvement specialists (EC, FD, NR) and an administrator (MK).

Funding statement: 'This work is supported by National Institute for Health Research and UK Research and Innovation, administered by the MRC: grant number MR/V02776X/1. The Trial Sponsor is the University of Exeter, ref: 1920/ Research Ethics and Governance Office, Lafrowda House, St Germans Road, Exeter, Devon, EX4 6TL. Neither the funder nor sponsor will have any role or ultimate authority in study design; collection, management, analysis, and interpretation of data; writing of the report; and the decision to submit the report for publication.

Competing interests: the authors confirm that they have no conflict of interests to declare

References

1. Darzi A. Quality and the NHS next stage review. Lancet. 2008;371(9624):1563-4.
2. Vincent C, Amalberti R. Safer Healthcare: Strategies for the Real World. Cham (CH): Springer Open; 2016.
3. Black N, Varaganum M, Hutchings A. Relationship between patient reported experience (PREMs) and patient reported outcomes (PROMs) in elective surgery. BMJ Qual Saf. 2014;23(7):534-42.
4. Doyle C, Lennox L, Bell D. A systematic review of evidence on the links between patient experience and clinical safety and effectiveness. BMJ Open. 2013;3(1).
5. Department of Health. Report of the Mid Staffordshire NHS Foundation Trust Public Enquiry. London: Department of Health; 2013.
6. @BorisJohnson. It is hard to find the words to express my debt to the NHS for saving my life. The efforts of millions of people across this country to stay home are worth it. Together we will overcome this challenge, as we have overcome so many challenges in the past. #StayHomeSaveLives [twitter post]12/04/2020 10/05/2020.

For peer review only - http://bmjopen.bmj.com/site/about/guidelines.xhtml

7. Aiken LH, Sloane DM, Bruyneel L, Van den Heede K, Griffiths P, Busse R, et al. Nurse staffing and education and hospital mortality in nine European countries: a retrospective observational study. Lancet. 2014;383(9931):1824-30.
8. Graham C, Kasbauer S, Cooper R, King J, Sizmur S, Jenkinson C, et al. An evaluation of a near real-time survey for improving patients' experiences of the relational aspects of care: a mixed-methods evaluation. Health Services and Delivery Research. Southampton (UK)2018.
9. Murrells T, Robert G, Adams M, Morrow E, Maben J. Measuring relational aspects of hospital care in England with the 'Patient Evaluation of Emotional Care during Hospitalisation' (PEECH) survey questionnaire. BMJ Open. 2013;3(1).
10. Aiken LH, Sloane D, Griffiths P, Rafferty AM, Bruyneel L, McHugh M, et al. Nursing skill mix in European hospitals: cross-sectional study of the association with mortality, patient ratings, and quality of care. Bmj Qual Saf. 2017;26(7):559-68.
11. Feo R, Conroy T, Jangland E, Muntlin Athlin A, Brovall M, Parr J, et al. Towards a standardised definition for fundamental care: A modified Delphi study. J Clin Nurs. 2018;27(11-12):2285-99.
12. Kitson A, Conroy T, Wengstrom Y, Profetto-McGrath J, Robertson-Malt S. Defining the fundamentals of care. Int J Nurs Pract. 2010;16(4):423-34.
13. Calvert MJ, Freemantle N. Use of health-related quality of life in prescribing research. Part 1: why evaluate health-related quality of life? J Clin Pharm Ther. 2003;28(6):513-21.
14. FDA-NIH Biomarker Working Group. BEST (Biomarkers, EndpointS, and other Tools) Resource Silver Spring and Bethesda (MD): Food and Drug Administration (US) and National Institutes of Health (US); 2016 [updated 2020. Available from: https://www.ncbi.nlm.nih.gov/books/NBK326791/.
15. Coulter A. Measuring what matters to patients. BMJ. 2017;356:j816.
16. Kelly L, Sizmur S, Kasbauer S, King J, Cooper R, Jenkinson C, et al. The Relational Aspects of Care Questionnaire: item reduction and scoring using inpatient and accident and emergency data in England. Patient Relat Outcome Meas. 2018;9:173-81.
17. Wilde Larsson B, Larsson G. Development of a short form of the Quality from the Patient's Perspective (QPP) questionnaire. J Clin Nurs. 2002;11(5):681-7.
18. Larsson BW, Larsson G, Chantereau MW, von Holstein KS. International comparisons of patients' views on quality of care. Int J Health Care Qual Assur Inc Leadersh Health Serv. 2005;18(1):62-73.
19. Canadian Nurses Association. CNA Brief to the National Advisory Committee on SARS and Public Health: Lessons learned and recommendations. Ottawa, Canada: Canadian Nurses Association; 2003.
20. Baumann A, Blythe J, Underwood J, Dzuiba J. Capacity, casualization, and continuity: the impact of SARS Toronto, Canada: University of Toronto, Faculty of Nursing and McMaster University, School of Nursing; 2003.
21. Registered Nurses Association of Ontario. SARS Unmasked: Celebrating Resilience, Exposing Vulnerability. A Report on the Nursing Experience with SARS in Ontario. Toronto: Registered Nurses Association of Ontario; 2003.
22. Dobbins M. Rapid review guidebook. Hamilton, ON: National Collaborating Centre for Methods and Tools; 2017.
23. Thompson-Coon J, Whear R, Abbott R, Bethel A, Cockcroft E, Cooper J, et al. Effectiveness of and barriers to fundamental nursing care procedures in patients with SARS-

For peer review only

COV-2 virus: a rapid systematic review. 28/07/2020 ed: PROSPERO 2020 CRD42020200914; 2020.
24. Ball JE, Bruyneel L, Aiken LH, Sermeus W, Sloane DM, Rafferty AM, et al. Post-operative mortality, missed care and nurse staffing in nine countries: A cross-sectional study. Int J Nurs Stud. 2018;78:10-5.
25. Ball JE, Murrells T, Rafferty AM, Morrow E, Griffiths P. 'Care left undone' during nursing shifts: associations with workload and perceived quality of care. BMJ Qual Saf. 2014;23(2):116-25.
26. Halcomb E, Davidson P, Hardaker L. Using the consensus development conference method in healthcare research. Nurse Res. 2008;16(1):56-71.
27. Nair R, Aggarwal R, Khanna D. Methods of formal consensus in classification/diagnostic criteria and guideline development. Semin Arthritis Rheum. 2011;41(2):95-105.
28. Waggoner J, Carline JD, Durning SJ. Is There a Consensus on Consensus Methodology? Descriptions and Recommendations for Future Consensus Research. Acad Med. 2016;91(5):663-8.
29. Campbell MK, Elbourne DR, Altman DG, Grp C. CONSORT statement: extension to cluster randomised trials. British Medical Journal. 2004;328(7441):702-8.
30. Hayes RJ, Moulton LH. Cluster Randomised Trials 2nd Edition. Florida: Chapman and Hall/CRC; 2017.
31. Moore GF, Audrey S, Barker M, Bond L, Bonell C, Hardeman W, et al. Process evaluation of complex interventions: Medical Research Council guidance. BMJ. 2015;350:h1258.
32. National Institute for Health and Care Excellence. Guide to the methods of technology appraisal. London: National Institute for Health and Care Excellence; 2013.
33. Johnson K, Gustafson D, Ewigman B, Provost L, Roper R. Using Rapid-Cycle Research to Reach Goals: Awareness, Assessment, Adaptation, Acceleration. AHRQ Publication No. 15-0036. Rockville (MD): Agency for Healthcare Research and Quality; 2015.
34. Sze S, Pan D, Nevill CR, Gray LJ, Martin CA, Nazareth J, et al. Ethnicity and clinical outcomes in COVID-19: A systematic review and meta-analysis. EClinicalMedicine. 2020;29:100630.
35. Downing A, Morris EJ, Corrigan N, Sebag-Montefiore D, Finan PJ, Thomas JD, et al. High hospital research participation and improved colorectal cancer survival outcomes: a population-based study. Gut. 2017;66(1):89-96.
36. Huis A, Schoonhoven L, Grol R, Donders R, Hulscher M, van Achterberg T. Impact of a team and leaders-directed strategy to improve nurses' adherence to hand hygiene guidelines: a cluster randomised trial. Int J Nurs Stud. 2013;50(4):464-74.
37. Sugg H, Russell A, Morgan L, Iles-Smith L, Richards D, Morley N, et al. Fundamental nursing care in patients with the SARS-CoV-2 virus: results from the 'COVID-NURSE' mixed methods survey into nurses' experiences of missed care and barriers to care. BMC Nursing in press.
38. FutureLearn. London: The Open University and The SEEK Group; 2021 [Available from: https://www.futurelearn.com.
39. NHS Improvement. Guide to developing and implementing ward and unit accreditation programmes, IG 09/19. London: NHS Improvement; 2019.
40. Mahoney FI, Barthel DW. Functional Evaluation: The Barthel Index. Md State Med J. 1965;14:61-5.

For peer review only

41. Collin C, Wade DT, Davies S, Horne V. The Barthel ADL Index: a reliability study. Int Disabil Stud. 1988;10(2):61-3.
42. Marshall J, On Behalf Of The Who Working Group On The Clinical Characteristics Of Covid-19 Infection,. A Core Outcome Measure Set for Studies of SARS-CoV2/COVID-19 infection Toronto, Canada: University of Toronto; 2020 [Available from: http://www.comet-initiative.org/Studies/Details/1528.
43. Kroenke K, Spitzer RL, Williams JB. The Patient Health Questionnaire-2: validity of a two-item depression screener. Med Care. 2003;41(11):1284-92.
44. Kroenke K, Spitzer RL, Williams JB, Monahan PO, Lowe B. Anxiety disorders in primary care: prevalence, impairment, comorbidity, and detection. Ann Intern Med. 2007;146(5):317-25.
45. EuroQol Research Foundation. EQ-5D-5L User Guide. Available from: https://euroqol.org/publications/user-guides2019.
46. Epstein EG, Whitehead PB, Prompahakul C, Thacker LR, Hamric AB. Enhancing Understanding of Moral Distress: The Measure of Moral Distress for Health Care Professionals. AJOB Empir Bioeth. 2019;10(2):113-24.
47. Corley MC, Elswick RK, Gorman M, Clor T. Development and evaluation of a moral distress scale. J Adv Nurs. 2001;33(2):250-6.
48. Medical Research Council. MRC Guidelines for Good Clinical Practice in Clinical Trials. London: Medical Research Council; 1998.
49. Department of Health. The Caldicott Committee Report on the Review of Patient-Identifiable Information. London: Department of Health; 1997.
50. Stata Corp. Stata version 16. Texas, USA: Stata Corp; 2021.
51. Graham JW. Missing Data: Analysis and Design, Statistics for Social and Behavioural Sciences. New York: Springer; 2012.
52. Rafferty AM, Philippou J, Fitzpatrick JM, Pike G, Ball J. Development and testing of the 'Culture of Care Barometer' (CoCB) in healthcare organisations: a mixed methods study. BMJ Open. 2017;7(8):e016677.
53. Finch TL, Girling M, May CR, Mair FS, Murray E, Treweek S, et al. Improving the normalization of complex interventions: part 2 - validation of the NoMAD instrument for assessing implementation work based on normalization process theory (NPT). BMC Med Res Methodol. 2018;18(1):135.
54. Rapley T, Girling M, Mair FS, Murray E, Treweek S, McColl E, et al. Improving the normalization of complex interventions: part 1 - development of the NoMAD instrument for assessing implementation work based on normalization process theory (NPT). BMC Med Res Methodol. 2018;18(1):133.
55. Gale NK, Heath G, Cameron E, Rashid S, Redwood S. Using the framework method for the analysis of qualitative data in multi-disciplinary health research. BMC Med Res Methodol. 2013;13:117.
56. QSR International. NVivo 10 for windows 2017 [Available from: www.qsrinternational.com/products_nvivo.aspx. .
57. Ritchie J, Spencer L, W OC. Qualitative research practice: A guide for social science students and researchers. London: Sage; 2003.
58. Creswell JW, Plano-Clark VL. Designing and conducting mixed methods research. Thousand Oaks: Sage; 2007.

For peer review only

59. Richards DA, Bazeley P, Borglin G, Craig P, Emsley R, Frost J, et al. Integrating quantitative and qualitative data and findings when undertaking randomised controlled trials. BMJ Open. 2019;9(11):e032081.
60. Rogers P. Using programme theory to evaluate complicated and complex aspects of interventions. Evaluation. 2008;14(1):29-48.
61. Funnell S.C., Rogers P.J. Purposeful Program Theory. Effective Use of Theories of Change and Logic Models. San Francisco: Jossey-Bass/Wiley; 2011.
62. Curtis L, Burns A. Unit Costs of Health and Social Care 2019. University of Kent, Canterbury: Personal Social Services Research Unit; 2019.
63. Department of Health. NHS reference costs 2018-2019. London: Department of Health; 2019.
64. INVOLVE. Briefing notes for researchers: involving the public in NHS, public health and social care research. Eastleigh: INVOLVE; 2012.
65. Health Research Authority. Safety reporting 2020 [Available from: https://www.hra.nhs.uk/approvals-amendments/managing-your-approval/safety-reporting/.
66. Health Research Authority. UK policy framework for health and social care research v3.3. London: National Health Service; 2017.
67. Richards DA, Hilli A, Pentecost C, Goodwin VA, Frost J. Fundamental nursing care: A systematic review of the evidence on the effect of nursing care interventions for nutrition, elimination, mobility and hygiene. Journal of Clinical Nursing. 2018;27(11-12):2179-88.
68. National Institute for Health Research. Strengthening our commitment to equality, diversity, inclusion and patient and public involvement and engagement (PPIE) 2020 [updated 01/05/2020. Available from: https://www.nihr.ac.uk/documents/strengthening-our-commitment-to-equality-diversity-inclusion-and-patient-and-public-involvement-and-engagement-ppie/24697.
69. World Medical Association. World Medical Association Declaration of Helsinki Ethical Principles for Medical Research Involving Human Subjects. Journal of the American Medical Association. 2013;310(20):2191-4.
70. World Health Organisation. 2019 novel Coronavirus Global research and innovation forum: towards a research roadmap. Geneva: World Health Organisation; 2020.

For peer review only

COLLEGE OF MEDICINE AND HEALTH

Research title: COVID-NURSE: evaluation of the effects of a COVID-specific fundamental nursing care protocol compared to care as usual on experience of care for non-invasively ventilated patients in hospital with the SARS-CoV-2 virus: a randomised controlled trial.

CONSENT FORM
FOR PATIENT PARTICIPANTS

Participant Identification Number: ☐☐☐☐☐☐☐☐☐

Name of Researcher: ______________________

Please initial box

1. I confirm that I have read the information sheet dated [2021-01-25] version no [5] for the above project. I have had the opportunity to consider the information, ask questions and have had these answered satisfactorily. ☐

2. I understand that my participation is voluntary and that I am free to withdraw at any time without giving any reason and without my legal rights being affected. ☐

3. I understand that relevant sections of my medical record and the data collected during the study may be looked at by members of the research team, individuals from the University of Exeter, Exeter Clinical Trials Unit or the Health Research Authority, where it is relevant to my taking part in this research.
I give permission for these individuals to have access to my records. ☐

4. I understand that data I provide for the study will be stored on servers all of which are hosted within the Europe Economic Area ☐

5. I understand that taking part involves questionnaire responses to be used for the purposes of research ☐

6. I understand that taking part involves inclusion in an archive for a period of up to 10 years ☐

7. I understand that taking part involves anonymized data being shared with other researchers for use in future research projects; ☐

8. I understand that taking part involves anonymized data being used in reports published in academic publication, project website and media publication; ☐

9. I understand that taking part involves anonymized data being used in public engagement activities ☐

For peer review only - http://bmjopen.bmj.com/site/about/guidelines.xhtml

Please initial box

10. I understand that ward level data will be collected for the trial and may be looked at by individuals from the University of Exeter, the NHS Trust and from regulatory authorities where it is relevant to my taking part in the research. I give permission for these individuals to have access to my records. ☐

11. I understand the research team would like my contact details do they can Let me know the results of the study **[optional item]** ☐

12. I understand that there may be circumstances in which confidential information may need to be disclosed if information shared suggests a risk to myself or others. ☐

13. I agree to take part in the above project. ☐

.... (Printed name of participant)	………………………….. (Signature of participant)	.... (Date)
.... (Printed name of researcher taking consent)	………………………….. (Signature of researcher)	.... (Date)

When completed: 1 copy for participant; 1 copy for researcher/project file

For peer review only - http://bmjopen.bmj.com/site/about/guidelines.xhtml

UNIVERSITY OF EXETER

COLLEGE OF MEDICINE AND HEALTH

Research title: COVID-NURSE: evaluation of the effects of a COVID-specific fundamental nursing care protocol compared to care as usual on experience of care for non-invasively ventilated patients in hospital with the SARS-CoV-2 virus: a randomised controlled trial.

CONSENT FORM
FOR NURSE PARTICIPANTS

Participant Identification Number: ☐☐☐☐☐☐☐☐☐

Name of Researcher: ______________________________

Please initial box

1. I confirm that I have read the information sheet dated [2021- 01-13] version no [4] for the above project. I have had the opportunity to consider the information, ask questions and have had these answered satisfactorily. ☐

2. understand that my participation in providing my data is voluntary and that I am free to withdraw at any time without giving any reason and without my legal rights being affected. ☐

3. I understand that I may also be asked to take part in a short online interview with follow up questions, which will be recorded for later transcription, at the end of the 6 week trial period by a researcher. ☐

4. If interviewed, the transcription will be anonymized to protect participant confidentiality. ☐

5. I understand that relevant sections of the data collected during the study may be looked at by members of the research team, individuals from the University of Exeter, Exeter Clinical Trials Unit or regulatory authorities, where it is relevant to my taking part in this research.
I give permission for these individuals to have access to my data. ☐

6. I understand that data I provide for the study will be stored on servers all of which are hosted within the Europe Economic Area ☐

7. I understand that taking part involves questionnaire responses to be used for the purposes of research ☐

8. I understand that taking part involves inclusion in an archive for a period of up to 10 years. Data will be stored securely at the University of Exeter. ☐

For peer review only

For peer review only - http://bmjopen.bmj.com/site/about/guidelines.xhtml

9. I understand that taking part involves anonymized data being shared with other researchers for use in future research projects; ☐

10. I understand that if my Trust has been allocated to the experimental intervention condition I will be asked to undertake some online training on the FutureLearn platform. If I choose to undertake this training, I will consent to the General Data Protection Regulation privacy policies of FutureLearn, which I can read on the FutureLearn course website. I understand that a specific course 'Learning Manager' at the University of Exeter may support and interact with me about the course. I understand that the Learning Manager will remove my email, name and any other personally identifiable information from my course records, before collating and sending anonymised records of training to be analysed by the research team. I understand that no records of my training will be seen by a researcher at my hospital. ☐

11. I understand the research team would like my contact details so they may contact me to participate in an interview and let me know the results of the study **[optional item]** ☐

12. I understand that taking part involves anonymized data being used in reports published in academic publication, project website and media publication; ☐

13. I understand that taking part involves anonymized data being used in public engagement activities. ☐

14. I understand that there may be circumstances in which confidential information may need to be disclosed if information shared suggests a risk to myself or others. ☐

15. I agree to take part in the above project. ☐

....	…………………………..	....
(Printed name of participant)	(Signature of participant)	(Date)
....	…………………………..	....
(Printed name of researcher taking consent)	(Signature of researcher)	(Date)

When completed: 1 copy for participant; 1 copy for researcher/project file

For peer review only - http://bmjopen.bmj.com/site/about/guidelines.xhtml
